# Inter-rater agreement of a newborn calf lung ultrasound scoring system

**DOI:** 10.1093/jvimsj/aalag067

**Published:** 2026-04-21

**Authors:** Ana C A Abreu, Sébastien Buczinski, Gabriela S Fregolon, Jobson F P Cajueiro, Viviani Gomes

**Affiliations:** Department of Internal Medicine, College of Veterinary Medicine and Animal Science, University of São Paulo, São Paulo 05508-270, Brazil; Département Des Sciences Cliniques, Faculté de Médecine Vétérinaire, Université de Montréal, Saint- Hyacinthe J2S 2M2, Québec, Canada; Department of Internal Medicine, College of Veterinary Medicine and Animal Science, University of São Paulo, São Paulo 05508-270, Brazil; Department of Internal Medicine, College of Veterinary Medicine and Animal Science, University of São Paulo, São Paulo 05508-270, Brazil; Department of Internal Medicine, College of Veterinary Medicine and Animal Science, University of São Paulo, São Paulo 05508-270, Brazil

**Keywords:** observer agreement, neonate calves, point-of-care, diagnostic imaging, lung clearance, perinate calf

## Abstract

**Background:**

Lung ultrasonography (LUS) is a reliable and noninvasive tool for detecting pulmonary abnormalities in neonatal calves. Although scoring systems for clinical decision-making are well established in human neonatology, equivalent validated systems for newborn calves are lacking for the perinatal period (0-48 h).

**Hypothesis/Objectives:**

Assess inter-observer agreement among veterinarians regarding a proposed lung ultrasound scoring system for newborn calves adapted from human neonatology score to detect pulmonary problems soon after birth.

**Animals:**

Fifty thoracic ultrasound items (25 images and 25 videos) from calves born in commercial Brazilian dairy herds were evaluated by 14 veterinarians with skills in calf LUS from Brazil, Canada, France, and the United States.

**Methods:**

After a 5-min instructional video, raters scored each item (0-4) based on lung aeration and consolidation patterns and graded media quality and diagnostic confidence on a 1-5 Likert scale. Agreement was assessed using raw percentage agreement (PA), Gwet’s AC2, Krippendorff’s alpha (α), and weighted Fleiss’ kappa (κ).

**Results:**

Inter-rater reliability for the 0-4 LUS score was very good: PA = 0.957 (95% confidence interval [CI], 0.940-0.973), AC2 = 0.845 (95% CI, 0.781-0.909), α = 0.839 (95% CI, 0.827-0.852), and κ = 0.849 (95% CI, 0.736-0.903). Median quality and diagnostic confidence were 4 (interquartile range [IQR] = 4-5). When restricted to high-confidence loops (scores 4-5), agreement increased slightly (PA = 0.960; AC2 = 0.880; α = 0.860).

**Conclusions and clinical importance:**

The high inter-observer agreement confirmed the reliability of the newborn calf LUS score. This score therefore potentially could be further validated to follow lung changes after delivery in neonatal calves.

## Introduction

Cattle morbidity is concentrated within the first 6 months of life (66%), with two-thirds of deaths occurring during the neonatal period (first 28 days after birth). The perinatal period, encompassing the critical first 24-48 h after delivery, accounts for most life-threatening complications.^1–3^ Neonatal mortality affects approximately 2%-7% of dairy calves,^1^^,^^4^ with perinatal deaths ranging from 1.3% to 11.2% across countries and peaks reaching 20.6%.^5^ A recent large-scale study reported perinatal mortality rates varying from 0% to 38.1% (mean, 7.6%),^6^ highlighting the substantial influence of early-life management on calf survival. These figures emphasize the urgent need for standardized early diagnostic strategies applicable within hours after birth.

The primary cause of perinatal mortality is the dystocia–asphyxia complex,^7^ which leads to tissue hypoxia and subsequent multi-organ failure, a condition that may be further aggravated by immunological immaturity and failure of passive transfer (FPT) secondary to cerebral anoxia.^8^ Pulmonary fluid clearance, essential for transitioning lungs from a fluid-filled intrauterine state to an air-filled postnatal organ, relies on phenotypic shift of the pulmonary epithelium from secretory to absorptive function.^9^ Impaired clearance likely contributes to early-onset pneumonia, because residual fluid compromises alveolar aeration and favors bacterial colonization.^10^ Additionally, peripartum hypoxia, meconium aspiration, and misuse of oroesophageal feeders can precipitate aspiration pneumonia, whereas septicemia may cause embolic pneumonia within the first week.^10^^,^^11^ Experimental murine models demonstrate that retained fluid or mucus, combined with impaired alveolar macrophage function, markedly increases susceptibility to severe respiratory infections, resulting in pneumonia in up to 51% of cases.^12^

Knowledge of perinatal pulmonary physiology in cattle has advanced by use of imaging methods including radiography and electrical impedance tomography (EIT), which enables real-time assessment of regional lung aeration and fluid clearance.^13^ In ovine models, EIT effectively monitors lung volume changes with sensitivity comparable to ultrasonography for detecting aeration dynamics.^14^ Despite high diagnostic resolution, EIT remains technically demanding and economically impractical for routine field use, necessitating more accessible modalities such as ultrasonography.

In human neonatology, point-of-care lung ultrasonography (LUS) has gained prominence as a safe, portable, radiation-free modality highly sensitive for monitoring alveolar aeration and pulmonary fluid clearance immediately after birth. Scoring systems have been implemented in human neonates and applied in lamb models studying preterm complications and asphyxia-related respiratory adaptation,^15^ and providing structured frameworks for thoracic assessment. Lung ultrasound enables real-time visualization of reverberation artifacts (A-lines), interstitial fluid indicators (B-lines), consolidations, and other pathological patterns, whereas systematic scoring improves diagnostic accuracy and reproducibility.^16^^,^^17^ Across different models,^18–24^ the underlying concept remains consistent: each thoracic zone receives a score (typically 0-3 or 0-4) summed across the thorax, with receiver operator characteristic curve-derived cut-offs guiding clinical decisions. The typical aeration trajectory progresses from a cloudy interstitial pattern dominated by B-lines to fully aerated lungs characterized by A-lines.

In calves, LUS has proven diagnostic accuracy for bronchopneumonia^25^ and good inter-rater agreement to detect consolidation,^26^ but its application during early life remains limited to detecting consolidation in early-onset bronchopneumonia and aspiration pneumonia associated with dystocia, asphyxia, and delayed fluid clearance.^27^^,^^28^ Beyond compromising oxygenation, dystocia may indirectly impair suckling and swallowing reflexes, decreasing colostrum intake and increasing FPT and aspiration pneumonia risk.^28–31^ Birth trauma also can cause undiagnosed rib fractures that restrict thoracic expansion, further contributing to respiratory impairment.^31^^,^^32^

Standardizing LUS scoring systems during the perinatal period (0-48 h) is essential to distinguish physiological from pathological changes, enable timely interventions, and promote judicious antimicrobial use. However, inter-rater agreement assessment is essential before implementing any new scoring system. We aimed to adapt a neonatal LUS scoring system for newborn calves derived from a previously proposed model^19^ and to assess inter-observer agreement among 14 veterinarians with variable experience. We hypothesized that the system would have high agreement among raters experienced in thoracic ultrasonography for detecting bronchopneumonia in calves, representing the first step toward using ultrasonography to assess perinatal cardiopulmonary adaptation to extrauterine life.

## Materials and methods

### Development of an ultrasonographic lung scoring system for perinatal calves

**Figure 1:**
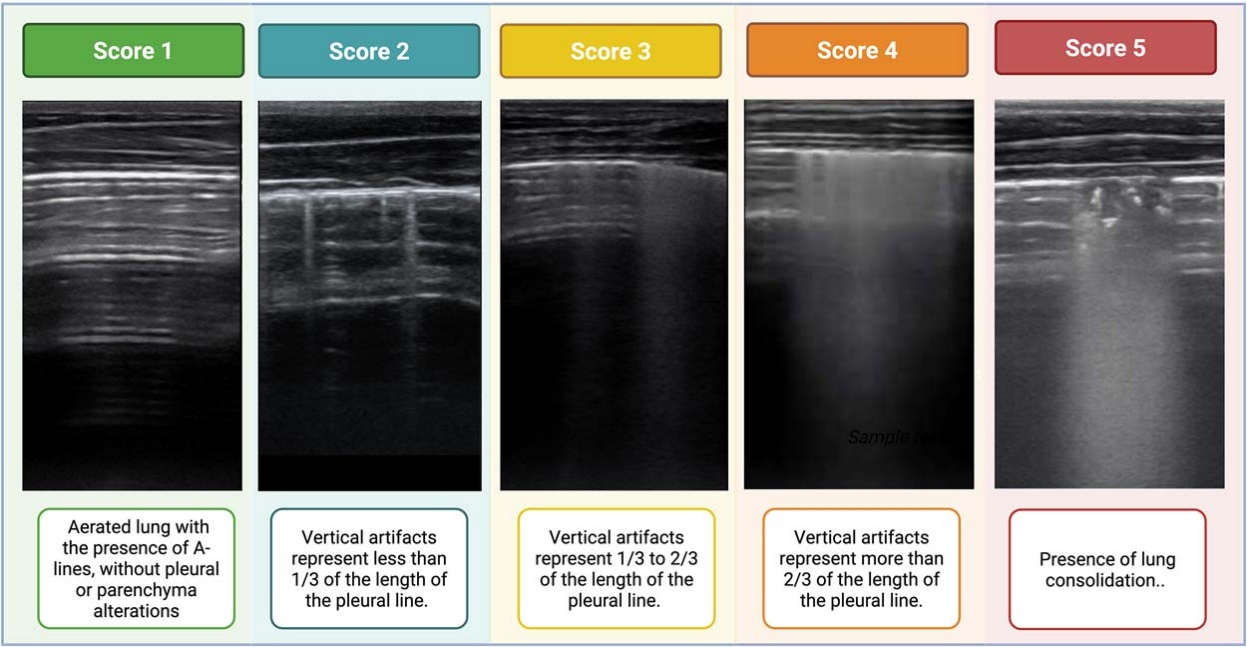
Representative ultrasonographic images illustrating the Newborn Calf Lung Ultrasound Score (NCLUS) (0–4). The images illustrate progressive degrees of pulmonary involvement, ranging from normal aeration to lung consolidation. Score 0: aerated lung with A-lines, without pleural or parenchymal alterations. Score 1: vertical artifacts occupying < 1/3 of the pleural line. Score 2: vertical artifacts occupying 1/3–2/3 of the pleural line. Score 3: vertical artifacts occupying > 2/3 of the pleural line. Score 4: presence of lung consolidation.

The Newborn Calf Lung Ultrasound Score (NCLUS) was adapted from a previous study.^19^ The original system included four categories describing progressive loss of lung aeration: score 0 corresponded to a normally aerated lung characterized by A-lines and a smooth pleural line; score 1 was defined by multiple well-spaced B-lines consistent with mild interstitial syndrome; score 2 represented confluent B-lines (“white lung”) without evidence of consolidation; and score 3 indicated subpleural consolidation with air bronchograms (≥ 0.5-1 cm). In our study, the intermediate B-line patterns (scores 1 and 2 in the previous model^19^) were refined to capture a more detailed gradation of interstitial involvement. The proportion of the pleural surface occupied by vertical artifacts (B-lines) in the maximal ultrasonographic frame was quantified and divided into three subcategories: less than one-third (<1/3), between one-third and two-thirds (1/3-2/3), and more than two-thirds (>2/3) of the pleural length. This refinement allowed the distinction between mild, moderate, and severe interstitial patterns while maintaining the same criteria for consolidation as in the original classification.^19^ The final NCLUS therefore consisted of five categories (0-4): scores 0-3 represented increasing severity of interstitial syndrome based on the relative extent of B-lines, and score 4 corresponded to lung consolidation which can either be associated with atelectasia or lung infection (Figure 1). This modified structure was designed to enhance the sensitivity of the scoring system for detecting intermediate aeration states in perinatal calves while preserving compatibility with neonatal lung ultrasound frameworks used in humans. For cine loops, the scoring was based on the most severe ultrasonographic pattern observed throughout the video sequence.

**Figure 2:**
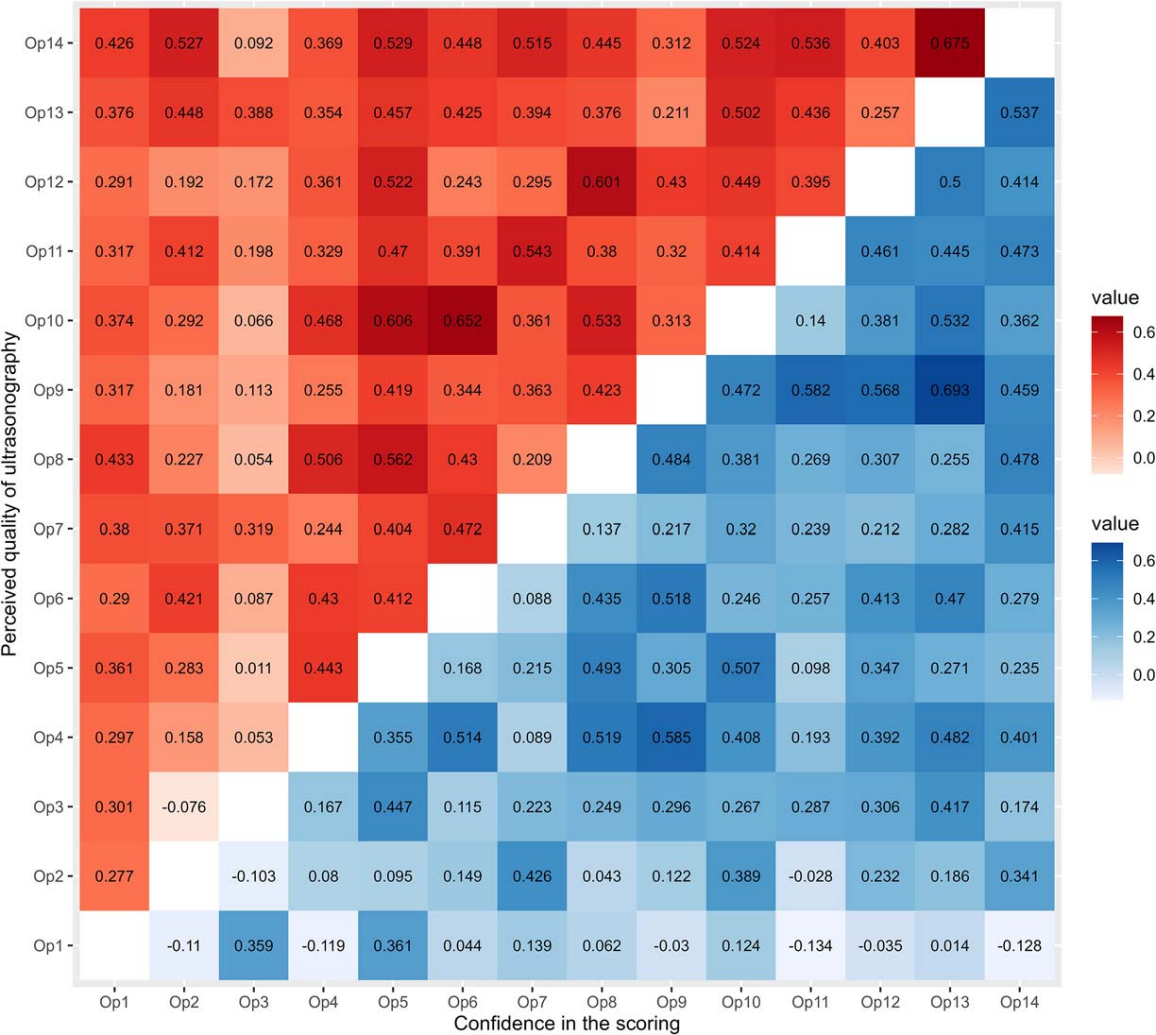
Heatmap representing the perceived media quality and diagnostic confidence correlations (Spearman ρ) between raters. The lower diagonal matrix represents the pairwise correlations of the 14 operators' confidence (1–5 Likert scale) in their score assignment. The upper diagonal matrix represents the pairwise correlations of the same operators' perception of media quality (1–5 Likert scale) when interpreting the 49 lung ultrasound items.

### Acquisition of ultrasonographic images and videos from neonatal calves

The repository used in our study included 1575 images and videos collected under two research projects approved by the Ethics committee University of São Paulo (CEUA 8547220523 and 5689310119). Ultrasound examinations were performed between 2020 and 2024 on commercial dairy farms in Brazil. The study included male and female Holstein suckling calves housed individually or in group pens and managed according to each farm’s standard health protocols.

Thoracic ultrasonography was conducted under manual restraint, with calves in sternal recumbency or standing, without sedation. Isopropyl alcohol (70%) was applied on unclipped skin as the transducing agent. A portable Mindray M5 Vet scanner equipped with a 38 mm linear rectal probe (Mindray 7L4s, 3.5-13 MHz) was used, operating at 6.2 MHz. Each hemithorax was systematically scanned from the 2nd to the 10th intercostal space, keeping the transducer parallel to the rib axis and recording 5-7 s cine loops per window. Single images were saved as PNG and videos as AVI, and then uploaded to cloud storage and an external hard drive.

The dataset was built by the first author (A.C.A.) and revised by the coauthors (V.G. and S.B.) before being sent to the raters. Fifty ultrasonographic items were included to represent the five lung aeration categories (0-4), with five static images and five video loops selected for each score category, ensuring diagnostic quality and a balanced category distribution. Selection criteria included image clarity, completeness of the pleural field, and visibility of key ultrasonographic artifacts (A-lines, B-lines, and consolidation). The chosen loops were intentionally selected after an initial overview to avoid overrepresentation of either normal or pathological patterns and to enable consistent analysis.

All loops were anonymized, trimmed to a uniform duration, and randomized. The 50 sequences then were randomly shuffled, converted into PNG still images and short video clips, and embedded in an online questionnaire so that each evaluator received the same set in a randomized order. Loops subsequently were classified according to the predefined NCLUS 0-4 scale described in section Development of ultrasonographic lung scoring system for perinatal calves.

### Rater training and rating procedure

Before rating, veterinarians completed a brief online training (20-30 min) with annotated examples for each NCLUS category and a short calibration set with feedback. Raters were blinded to farm, animal identity, timepoint, and to one another’s assessments.

Each veterinarian independently scored all 50 loops using the predefined NCLUS scale (0-4) described in section Development of ultrasonographic lung scoring system for perinatal calves. One categorical score per loop was recorded. Each intercostal space (ICS) was scored 0-4 (aerated lung to consolidation).

### Questionnaire design

An electronic questionnaire was created in parallel with the image selection process and consisted of two sections. The first section collected demographic and educational data from the raters: sex, year of graduation, proportion of working time devoted to ultrasonography in cattle, and predominant type of postgraduate training (clinically oriented, such as residency, or research oriented, such as MSc or PhD). A small tutorial was built to quickly present the NCLUS classification system. The second section presented 50 thoracic ultrasound items in random order. For each sequence, participants assigned one of the 5 lung aeration scores (0-4), rated their confidence in that decision on a 5-point Likert scale from 1 (not confident) to 5 (very confident). and used the same scale to rate image quality. A free-text field allowed specific comments on individual images. The online questionnaire (Google Forms) was built by the first author and reviewed by VG and SB.

Fourteen raters were recruited to provide a small but representative sample of veterinarians interested in bovine medicine, bovine respiratory disease, and LUS. They were drawn from Brazil, Canada, United States, France, and Italy on the basis of previous collaborations with the corresponding author and previous knowledge on LUS used for bronchopneumonia diagnosis in calves. All raters received standardized instructions through a tutorial available in English and Portuguese, which included representative examples of each score (0-4), descriptions of typical ultrasonographic artifacts (A-lines, B-lines, consolidations), and guidance on completing the questionnaire. The tutorial was provided in both video and PDF formats and is available as Supplementary File 2.

### Sample size and statistical analyses

#### Sample size justification

All analyses were performed using the open-access R software.^33^ Sample size was determined based on different power simulations using the power sample size (*pwrss*) package. Different plausible scenarios were constructed to assess the difference between theoretical Fleiss κ value (from 0 to 1, by 0.05 increment) and lower bound of its 95% confidence interval (CI) based on various numbers of raters (from 2 to 16, by 2 increment) for 25, 50, 75, and 100 rated objects. For all scenarios with at least 8 raters, this difference was <0.1 and judged clinically acceptable. The final chosen scenario included 50 assessments, comprising 25 images and 25 videos, evaluated by more than 10 different reviewers, excluding the authors ACAA, SB, and VG who participated in the media selection process (Supplementary File 1). Fourteen raters were invited to complete the assessment, accounting for possible absence of response or invalid questionnaires.

**Figure 3:**
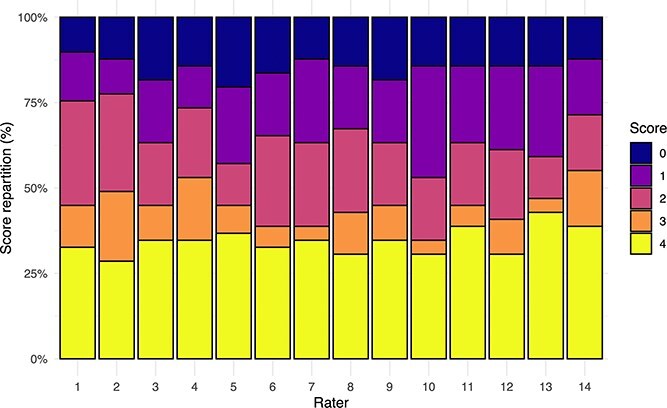
Distribution of lung ultrasound scores (0-4) assigned by each evaluator. Stacked bar plots represent the relative frequency (%) of each score per rater. The predominance of consistent color patterns across raters indicates high interobserver agreement and the robustness of the scoring system for use in neonatal calf lung assessment.

#### Statistics analyses

Descriptive statistics (median and interquartile range [IQR]) were computed for evaluator characteristics, image quality scores, and diagnostic confidence ratings. Analyses exploring the relationship between image quality and diagnostic confidence were performed using non-parametric Spearman correlation. Inter-rater perception of image quality versus diagnostic confidence was explored for all possible rater pairs using a non-parametric Spearman-ρ correlogram. The strength of correlation was interpreted based on a previous study^34^ using negligible (ρ < 0.10), weak (0.10-0.39), moderate (0.40-0.69), strong (0.70-0.89), and very strong (≥ 0.90).

Agreement was evaluated using the *irrCAC* and *irr* R packages.^35^ Inter-observer agreement was evaluated using four complementary coefficients: raw percentage agreement (PA), Gwet’s agreement coefficient type 2 (AC2), Krippendorff’s α (α), and weighted Fleiss’ κ (κ). These indices were selected in the absence of a gold standard reference for inter-rater agreement determination. Weighted versions of the chance-adjusted coefficients were applied to account for the ordinal nature of the scoring scale. The selection and interpretation of agreement coefficients followed standard methodological references.^36^^,^^37^ Ninety-five percent CI for PA and AC2 were calculated using normal variance approximation, whereas those for α and κ were obtained from 2000 bootstrap replicates. The strength of agreement was interpreted according to Altman’s benchmarks^38^: poor (<0.20), fair (0.21-0.40), moderate (0.41-0.60), good (0.61-0.80), and very good (0.81-1.00).

The rater-based prevalence of each score category also was recorded. Differences among raters were assessed with a χ-squared test, using *P* < .05 as the threshold for statistical significance.

## Results

The 14 contacted veterinarians participated in the evaluation and scoring of media files. The median year of graduation was 2007 (IQR, 1999-2010; range, 1988-2018). Most raters graduated from Brazilian universities (*n* = 9), followed by French (*n* = 3), Italian (*n* = 1), and American (*n* = 1) veterinary schools. Their current professional locations were Brazil (*n* = 9), France (*n* = 2), Canada (*n* = 1), Italy (*n* = 1), and the United States (*n* = 1). Eight of 14 raters were female. All raters had postgraduate training including clinical internship (*n* = 1) or residency (*n* = 6), board certification (American College of Veterinary Internal Medicine [ACVIM], *n* = 2; European College of Bovine Health Management [ECBHM], *n* = 1) or research qualification with a Master's (*n* = 2) and PhD (*n* = 10) degrees.

Of the 50 ultrasonographic items (25 static images and 25 video loops) submitted for evaluation, one image (corresponding to a normal aeration/A-line) was not recorded in the online form because of a submission error and therefore was excluded from the final dataset. Consequently, a total of 49 items (25 loops and 24 images) were included in the analysis.

All calculated indicators were between good and very good for chance-adjusted agreement and very good for percentage of raw agreement (Table 1). The percentage of raw agreement was 0.96 (95% CI, 0.94-0.97), whereas the weighted Fleiss’ κ was 0.85 (95% CI, 0.74-0.90) and the Krippendorff’s α was 0.84 (95% CI, 0.83-0.85). Similarly, the Gwet’s AC2 was 0.85 (95% CI, 0.78-0.91).

**Table 1:** Agreement indicators among 14 veterinarians scoring online lung ultrasound items obtained from neonatal calvves.

**Indicator Estimate 95%**	**Estimate**	**95% CI**
**PA**	0.957	0.940–0.973
**AC2**	0.845	0.781–0.909
***K*_Fleiss_ (weighted)**	0.849	0.736–0.903
**Krippendorff's α**	0.839	0.827–0.852

Displayed values include raw percentage of agreement (PA), weighted Fleiss’ κ (κ), Krippendorff’s α (α), and Gwet’s agreement coefficient type 2 (AC2), each with their respective 95% confidence intervals.

The correlations among the different raters for perceived recording quality and diagnostic confidence are presented in Figure 2. Considerable individual variation was evident for both variables, as reflected by the dispersion of Spearman coefficients. The median (IQR) Spearman’s ρ was 0.14 (0.05-0.30) for image quality and 0.15 (0-0.24) for confidence, values that in most evaluator combinations correspond to weak correlations.

The distribution of lung ultrasound scores (0-4) assigned by each rater is shown in Figure 3. Despite slight numerical differences between some raters and particular score proportions, the global χ-squared test was not significant (*P* = .75), which was compatible with an absence of significant differences between and within the raters.

Restricting the analysis to images rated with high diagnostic confidence (Likert scores 4-5) produced few changes in all chance-adjusted coefficients (AC2 = 0.88; α = 0.86). Collectively, these findings demonstrated good to very good agreement after chance adjustment and confirmed the robustness of the scoring system for being potentially used for assessment of calves just after birth for clinical and research applications.

## Discussion

We found very good agreement beyond chance among veterinarians evaluating the same LUS images in calves using an adapted version of the neonatal lung scoring assessment used by pediatricians for babies.^19^ The validation of scoring systems requires, as an essential step, the assessment of their inter-rater reliability, because reproducibility is a prerequisite for ensuring applicability in both research and clinical practice.^39^^,^^40^ Although the use of ultrasonography has been expanding in bovine medicine, its application in neonatal calves remains limited and poorly standardized. Therefore, we chose as a first step to assess inter-observer agreement as a fundamental step toward consolidating the scoring technique in this context.

A recent systematic review in human medicine identified 32 different pulmonary ultrasound scores, but only 13 (40.6%) had undergone formal validation.^41^ Most of these studies focused on criterion validity, correlating ultrasound scores with oxygenation parameters and clinical outcomes, and only a few also evaluated reliability using inter-rater reproducibility analyses, typically performed by two experienced raters in blinded settings. According to a previous study,^42^ the validation of clinical scoring systems involves sequential testing of both reliability (the consistency of measurements across raters or time), and validity (the extent to which the tool truly measures the construct it was designed to assess). Among the existing models, the previously proposed scoring system^19^ remains one of the most comprehensively validated, demonstrating strong criterion validity by its correlation with oxygenation indices and predictive value for surfactant treatment in preterm infants. These observations emphasize the need for stepwise development and validation of scoring systems, starting from reliability assessment and moving toward formal validation linking ultrasound findings with physiological indicators of respiratory function.

In our study, after a brief 5-min tutorial explaining the scoring system, we assessed inter-rater reliability across five lung ultrasound scores (0-4), ranging from normally aerated lung to extensive consolidation. We did not attempt to classify additional lesion categories to make the evaluation simpler, and restricted evaluation to the most frequently observed changes in lung ultrasound of perinatal calves. Previous studies in human medicine^43^ suggest that more such subtle variations, such as minor differences in brightness, texture, and dynamics of B-line artifacts or transitions between interstitial and early consolidation patterns, can be better characterized using automated approaches, including spectrograms and computer-aided diagnostic algorithms.

We chose to submit the scoring assessment to a representative panel of veterinarians involved in cattle practice, respiratory medicine, and with skills in thoracic ultrasonography. Inter-rater agreement was very good. All raters had prior experience with LUS, and the study was designed to reflect field conditions. These findings are consistent with previous research in human medicine, where structured lung ultrasound scores demonstrated good reproducibility among raters with different levels of experience.^19–44^ In veterinary medicine, moderate to high inter-observer agreement for detecting lung consolidation in calves with respiratory disease has been described in a review of thoracic ultrasonography applications in calves.^45^ However, a recent study^46^ showed that practitioners using a quick thoracic ultrasonography protocol to diagnose calf pneumonia achieved only moderate agreement compared with an experienced operator, emphasizing that diagnostic variability may persist in field applications even with simplified protocols. Unlike most studies in humans, that validated their scores by correlating ultrasonographic findings with oxygenation parameters, our study focused exclusively on inter-rater reliability, assessing whether a practical scoring system could be consistently applied under real farm conditions. Our findings demonstrate that the score is reliable when the same ultrasonographic loops are evaluated by different observers, providing a solid foundation for its subsequent validation. Future studies therefore should aim to determine the validity of the proposed score in live neonatal calves by investigating its correlation with clinical and physiological indicators of respiratory distress such as arterial oxygenation or acid–base balance, as previously described in human neonatology.^19^

Because our aim was to assess the inter-rater agreement of a practical scoring system rather than to provide exhaustive training on all possible pulmonary alterations, the tutorial was intentionally brief. Although the median and IQR scores for image quality and diagnostic confidence were high across raters, the relatively low Spearman correlation coefficients (ρ = 0.14 [0.05-0.30] for image quality and 0.15 [0-0.24] for diagnostic confidence) indicate that scoring confidence was substantially influenced by individual perception rather than by image quality. This variability reflects differences in how raters interpret visual features as markers of good image quality rather than inconsistency or poor reliability. Moreover, diagnostic confidence is inherently subjective, influenced by behavioral and self-assessment tendencies. Some raters are consistently confident, whereas others remain cautious even when correct. Therefore, interpretation of both image quality and confidence should consider the influence of personal perception rather than technical limitations. These findings are in accordance with a similar study design focused on lung sound auscultation quality and confidence in the diagnosis, which may be more intrinsically related to unmeasured evaluator characteristics such as prior experience or visual interpretation style than to the intrinsic properties of the image or video itself.^39^

Unlike studies performed in controlled hospital environments,^19–44^ our evaluation was conducted under commercial farm conditions with active calves and variable settings. Although our approach standardized the assessment by having all raters evaluate the same image sets, it did not account for potential variability related to image acquisition, such as probe positioning or handling. A similar limitation was previously mentioned when ultrasonographic loops were analyzed offline in feedlot calves.^47^ Future studies therefore should combine offline readings with a live phase in which independent raters both acquire and score LUS on the same calves in the field to quantify the impact of acquisition on reliability and to establish the external validity of the score.

Our study had some limitations. As previously mentioned, we selected raters who had substantial background in LUS in cattle. Therefore, the level of agreement found cannot be extrapolated to raters with various levels of expertise. However, this type of scoring system was not designed to be used by people without a minimal level of expertise in LUS in cattle. Also, this agreement may have been lower if the raters had to perform ultrasonography by themselves as opposed to interpreting the same items online. Moreover, although we believe that the tutorial was useful as a way to standardize scoring, we cannot prove its usefulness in the absence of any control group scoring the files without prior exposure to the tutorial.

In conclusion, the proposed LUS scoring system for perinatal calf assessment demonstrated high agreement among selected raters with brief online training. These findings represent a first necessary step toward standardized image interpretation. Future studies should operationalize how the score can be used in practice to evaluate responsiveness in the early postnatal period and establish validity by correlating scores with indicators of cardiorespiratory distress and with outcomes in the first weeks of life.

## Supplementary Material

aalag067_Supplemental_Files

## Off-label antimicrobial declaration

Authors declare no off-label use of antimicrobials.

## Institutional animal care and use committee or other approval declaration

Approved by the Ethics Committee on the Use of Animals of the School of Veterinary Medicine and Animal Science, University of São Paulo (CEUA 8547220523 and 5689310119).

## Abbreviations

AC2: Gwet’s agreement coefficient type 2
α: Krippendorff’s α
CI: confidence interval
FPT: failure of passive immune transfer
ICC: intraclass correlation coefficient
ICS: intercostal space
IQR: interquartile range
KFleiss: Fleiss’ κ
LUS: lung ultrasound
NCLLUS: Newborn Calf Lung Ultrasound Score
MHz: megahertz
PA: percentage agreement
ROC: receiver operating characteristic
