# Supplementary material for: Inter-rater agreement of a newborn calf lung ultrasound scoring system
Source: J Vet Intern Med. 2026 Apr 21;40(2):aalag067. doi: 10.1093/jvimsj/aalag067 (PMC13098365; doi:10.1093/jvimsj/aalag067)
Supplement: aalag067_Supplemental_Files [file aalag067_supplemental_files.zip › supplementaru_file_1_aalag067.pdf]

Scoring of Pulmonary Ultrasound Images in Bovine Neonatology: Reliability Assessment

Target Audience: Veterinarians and veterinary medicine students with experience in pulmonary ultrasound image analysis in calves.

Purpose of the Questionnaire: The purpose of this questionnaire is to understand how veterinarians and students classify ultrasound pulmonary images of neonatal calves, according to the scoring system proposed by our team. This research aims to enhance the reliability of this score. We kindly ask that you respond to the questions related to the ultrasound window images and/or videos. The materials were captured under clinical field conditions in neonatal calves, with or without symptoms of respiratory disorders, from dairy production systems and feedlots.

Access the Video: In the link below, you will find an explanatory video about each scoring point and project details. Please click the following link to access it: https://youtu.be/_LjKEUsBfMo

Recommendations for Responding to the Questionnaire:

A) It is important to review the images calmly for better navigation of the questionnaire.
B) The videos can be watched as many times as needed. If preferred, you can slow down the video and pause it for clearer visibility of the image.
C) For each image or video, you will be asked to evaluate it according to the representative score.

The scoring system is based on the worst score in a presented loop. For example if almost all the loop is normal but you find a small section with a higher score, the score attributed should be the score with the higher value.

Scoring System (5-levels scale):

0: Aerated lung with the presence of A-lines, with no pleural alteration.
1: In the ultrasound window, vertical artifacts represent less than 1/3 of the length of the pleural line.
2: In the ultrasound window, vertical artifacts represent 1/3 to 2/3 of the length of the pleural line.
3: In the ultrasound window, vertical artifacts represent more than 2/3 of the length of the pleural line.
4: Presence of lung consolidation.

Duration: The questionnaire takes approximately 30 minutes to complete.

We sincerely thank you for the time you will dedicate to this study voluntarily.

For any additional information, please do not hesitate to contact us:

Ana Carolina Araujo Abreu (anaabreu@usp.br)

Viviani Gomes (viviani.gomes@usp.br)

Sébastien Buczinski (s.buczinski@umontreal.ca)

* Indica uma pergunta obrigatória

1. E-mail *

____

2. E-mail *

____

3. Gender?

Marcar apenas uma oval.

- ( ) Male
- ( ) Female
- ( ) I prefer not to say
- ( ) Outro: _______________

4. Are you a veterinarian? *

Marcar apenas uma oval.

- ( ) Yes
- ( ) I'm a veterinary medicine student
- ( ) Other
- ( ) Outro: _______________

5. In what year did you obtain your veterinary degree? (If you are a student, indicate the expected year of graduation)

____

6. In which university or veterinary school + country did you obtain your veterinary degree? (If you are a student, please indicate your educational institution, country and year of study)

____

7. If you're still a student, tell us which year of university you're in. (Postgraduate: Classify as master's, PhD, etc.)

____

8. In which country do you practice veterinary medicine?

____

9. Have you completed any additional training or specialization in veterinary medicine?

   Marcar apenas uma oval.

   ◯ Yes

   ◯ No

10. What percentage of your work is with cattle?
    Every number increases by 10%, for example: 0 represents 0%, 1 represents 10%.

    Marcar apenas uma oval.

1	2	3	4	5	6	7	8	9	10
◯	◯	◯	◯	◯	◯	◯	◯	◯	◯

11. What is your experience with lung ultrasound in calves (from 1 almost no experience to 10 high experience)? *

Marcar apenas uma oval.

	1	2	3	4	5	6	7	8	9	10	
Low	◯	◯	◯	◯	◯	◯	◯	◯	◯	◯	High

For the next questions, we ask you to indicate which score you think is appropriate for each image or video:

We have included 25 images and 25 videos; please take your time when responding.

Image 1:

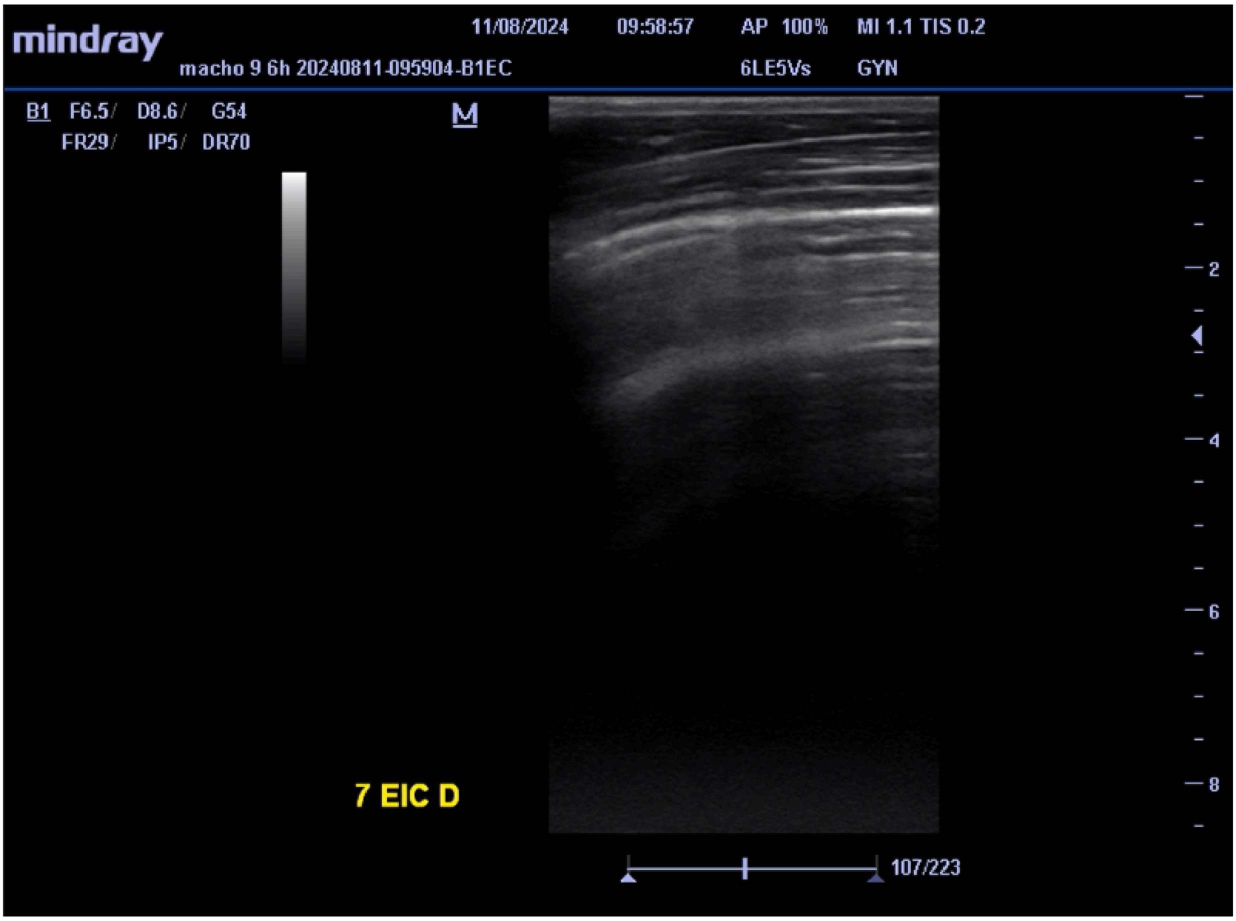

12. What score would you give this image? *

Marcar apenas uma oval.

- 0: Aerated lung with the presence of A-lines, with no pleural alteration.
- 1: In the ultrasound window, vertical artifacts represent less than 1/3 of the length of the pleural line.
- 2: In the ultrasound window, vertical artifacts represent 1/3 to 2/3 of the length of the pleural line.
- 3: In the ultrasound window, vertical artifacts represent more than 2/3 of the length of the pleural line.
- 4: Presence of consolidation.

13. How confident are you in your choice? *

Marcar apenas uma oval.

	1	2	3	4	5	
Not						Very confident

14. How do you rate the quality of the image? *

Marcar apenas uma oval.

	1	2	3	4	5	
Low						High quality

15. Note (if any)

Image 2:

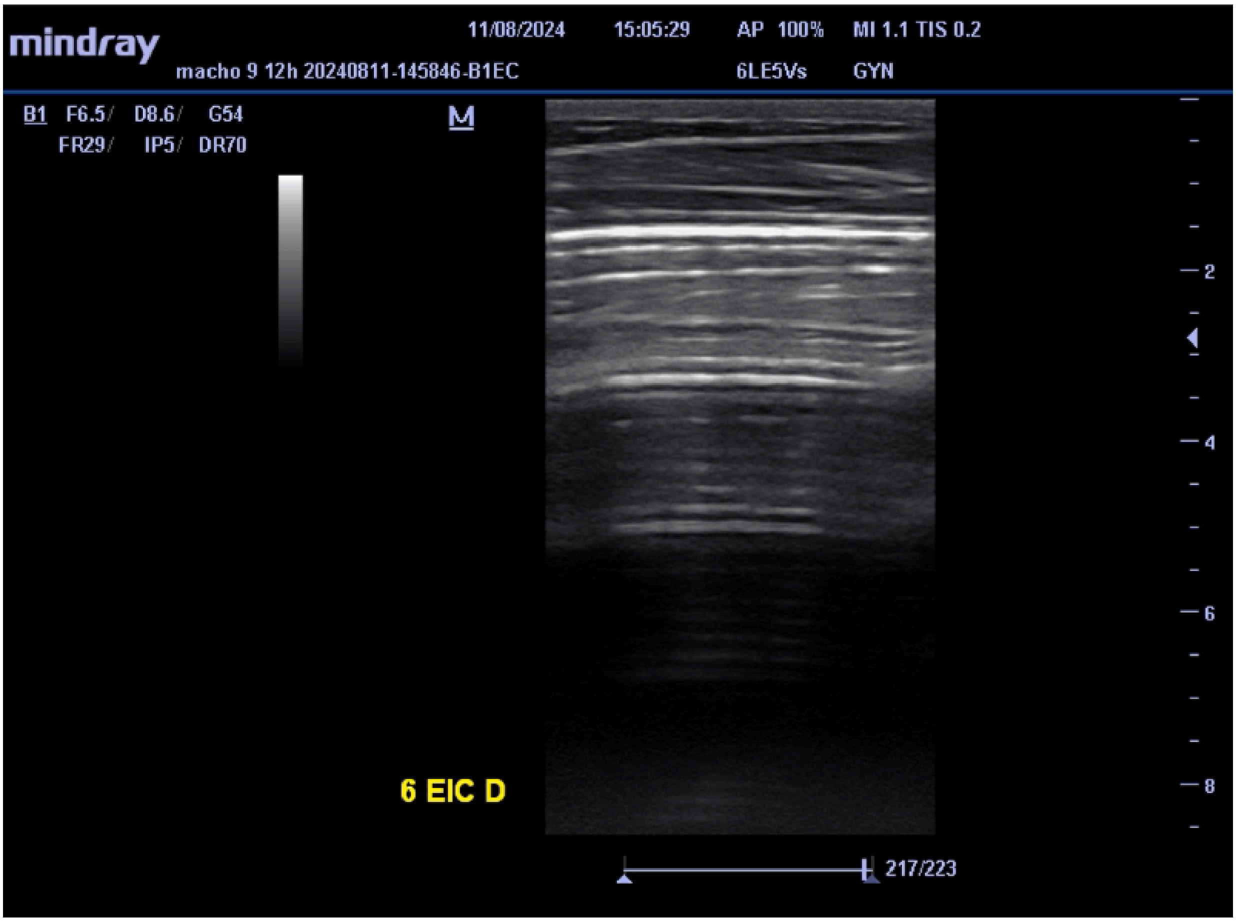

16. What score would you give this image? *

Marcar apenas uma oval.

- 0: Aerated lung with the presence of A-lines, with no pleural alteration.
- 1: In the ultrasound window, vertical artifacts represent less than 1/3 of the length of the pleural line.
- 2: In the ultrasound window, vertical artifacts represent 1/3 to 2/3 of the length of the pleural line.
- 3: In the ultrasound window, vertical artifacts represent more than 2/3 of the length of the pleural line.
- 4: Presence of consolidation.

17. How confident are you in your choice? *

Marcar apenas uma oval.

	1	2	3	4	5	
Not	◯	◯	◯	◯	◯	Very confident

18. How do you rate the quality of the image? *

Marcar apenas uma oval.

	1	2	3	4	5	
Low	◯	◯	◯	◯	◯	High quality

19. Note (if any)

____

Image 3:

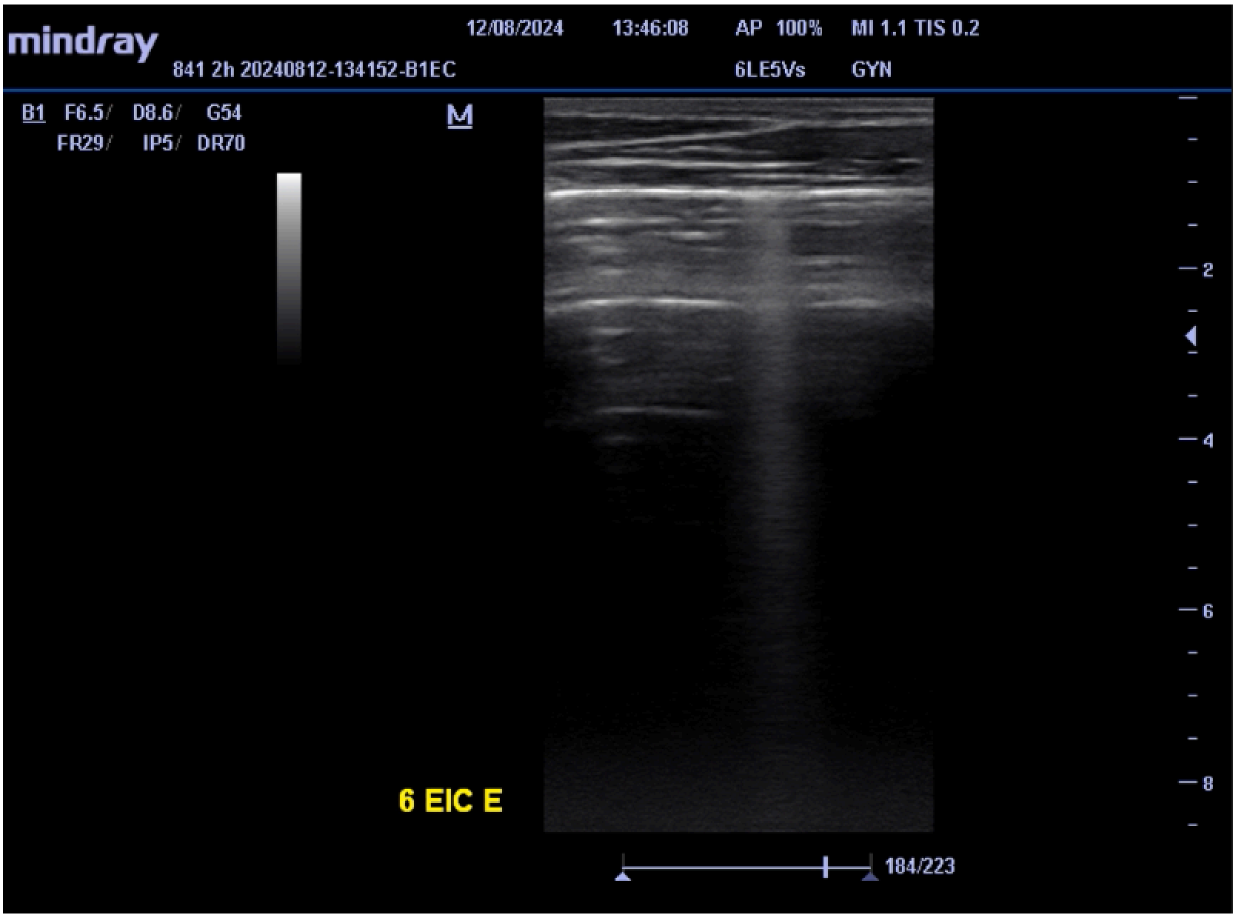

20. What score would you give this image? *

Marcar apenas uma oval.

- 0: Aerated lung with the presence of A-lines, with no pleural alteration.
- 1: In the ultrasound window, vertical artifacts represent less than 1/3 of the length of the pleural line.
- 2: In the ultrasound window, vertical artifacts represent 1/3 to 2/3 of the length of the pleural line.
- 3: In the ultrasound window, vertical artifacts represent more than 2/3 of the length of the pleural line.
- 4: Presence of consolidation.

21. How confident are you in your choice? *

Marcar apenas uma oval.

	1	2	3	4	5	
Not	◯	◯	◯	◯	◯	Very confident

22. How do you rate the quality of the image? *

Marcar apenas uma oval.

	1	2	3	4	5	
Low	◯	◯	◯	◯	◯	High quality

23. Note (if any)

Image 4:

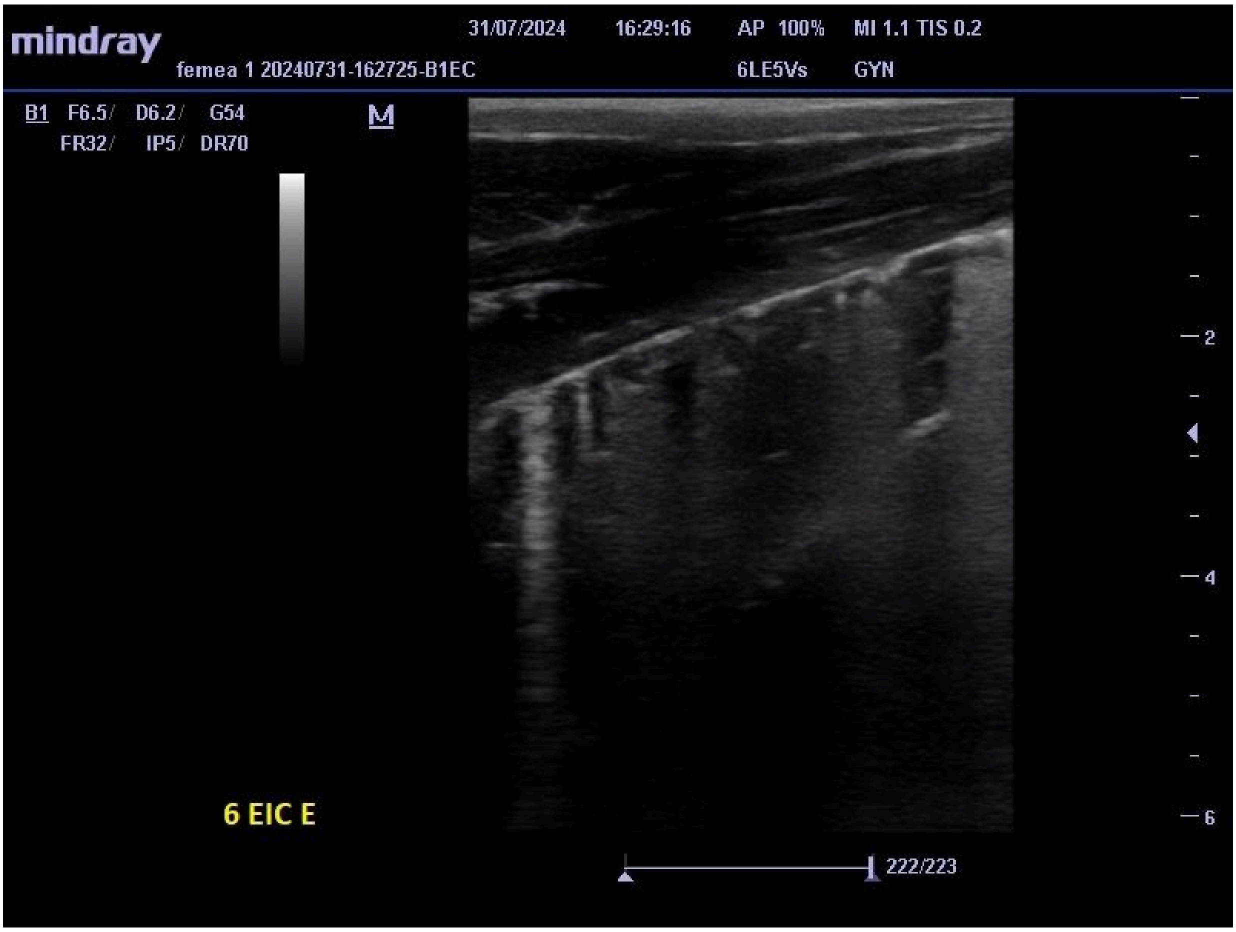

24. What score would you give this image? *

Marcar apenas uma oval.

- 0: Aerated lung with the presence of A-lines, with no pleural alteration.
- 1: In the ultrasound window, vertical artifacts represent less than 1/3 of the length of the pleural line.
- 2: In the ultrasound window, vertical artifacts represent 1/3 to 2/3 of the length of the pleural line.
- 3: In the ultrasound window, vertical artifacts represent more than 2/3 of the length of the pleural line.
- 4: Presence of consolidation.

25. How confident are you in your choice? *

Marcar apenas uma oval.

	1	2	3	4	5	
Not	◯	◯	◯	◯	◯	Very confident

26. How do you rate the quality of the image? *

Marcar apenas uma oval.

	1	2	3	4	5	
Low	◯	◯	◯	◯	◯	High quality

27. Note (if any)

____

____

____

____

____

28. What score would you give this image? *

Marcar apenas uma oval.

◯ 0: Aerated lung with the presence of A-lines, with no pleural alteration.

◯ 1: In the ultrasound window, vertical artifacts represent less than 1/3 of the length of the pleural line.

◯ 2: In the ultrasound window, vertical artifacts represent 1/3 to 2/3 of the length of the pleural line.

◯ 3: In the ultrasound window, vertical artifacts represent more than 2/3 of the length of the pleural line.

◯ 4: Presence of consolidation.

29. How confident are you in your choice? *

Marcar apenas uma oval.

	1	2	3	4	5	
Not	◯	◯	◯	◯	◯	Very confident

30. How do you rate the quality of the image? *

Marcar apenas uma oval.

	1	2	3	4	5	
Low	◯	◯	◯	◯	◯	High quality

31. Note (if any)

____

____

____

____

____

Image 5

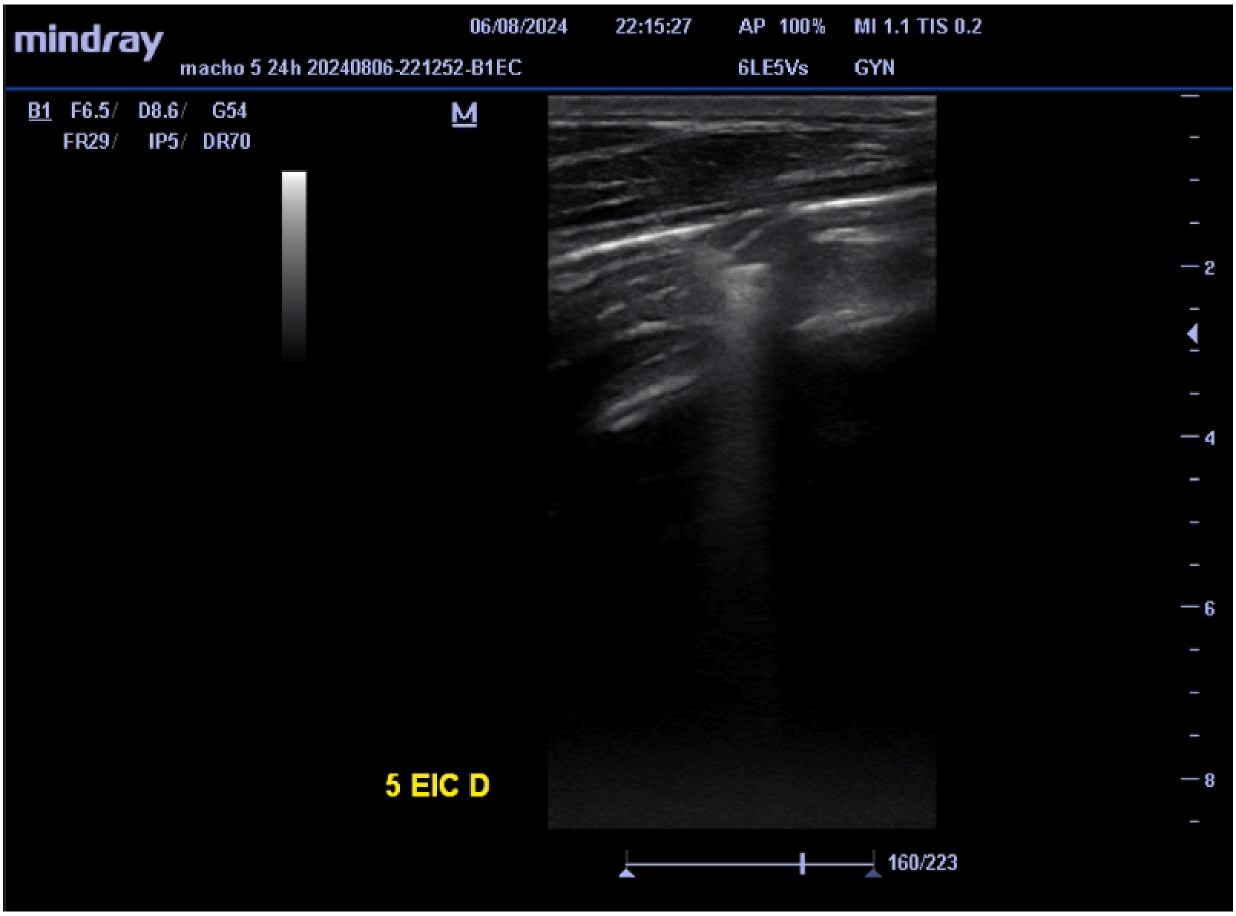

32. What score would you give this image? *

Marcar apenas uma oval.

- 0: Aerated lung with the presence of A-lines, with no pleural alteration.
- 1: In the ultrasound window, vertical artifacts represent less than 1/3 of the length of the pleural line.
- 2: In the ultrasound window, vertical artifacts represent 1/3 to 2/3 of the length of the pleural line.
- 3: In the ultrasound window, vertical artifacts represent more than 2/3 of the length of the pleural line.
- 4: Presence of consolidation.

33. How confident are you in your choice? *

Marcar apenas uma oval.

	1	2	3	4	5	
Not	◯	◯	◯	◯	◯	Very confident

34. How do you rate the quality of the video? *

Marcar apenas uma oval.

	1	2	3	4	5	
Low	◯	◯	◯	◯	◯	High quality

35. Note (if any)

____

____

____

____

____

Image 6

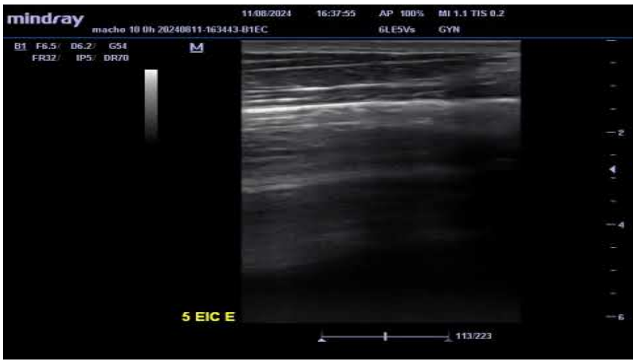

http://youtube.com/watch?v=LMBDm-56rr0

36. What score would you give this image? *

Marcar apenas uma oval.

- 0: Aerated lung with the presence of A-lines, with no pleural alteration.
- 1: In the ultrasound window, vertical artifacts represent less than 1/3 of the length of the pleural line.
- 2: In the ultrasound window, vertical artifacts represent 1/3 to 2/3 of the length of the pleural line.
- 3: In the ultrasound window, vertical artifacts represent more than 2/3 of the length of the pleural line.
- 4: Presence of consolidation.

37. How confident are you in your choice? *

Marcar apenas uma oval.

	1	2	3	4	5	
Not						Very confident

38. How do you rate the quality of the video? *

Marcar apenas uma oval.

	1	2	3	4	5	
Low						High quality

39. Note (if any)

Image 7

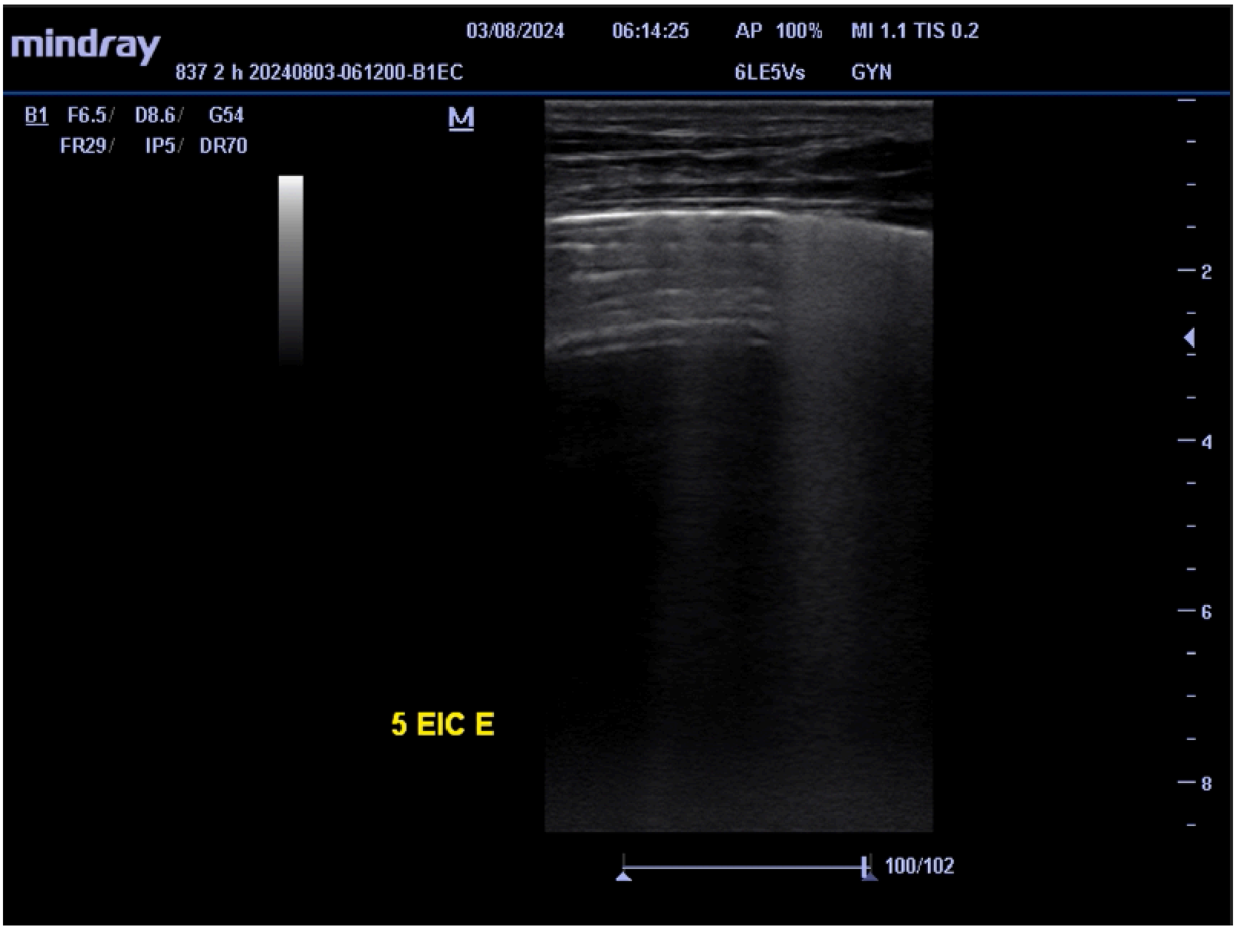

40. What score would you give this image? *

Marcar apenas uma oval.

- 0: Aerated lung with the presence of A-lines, with no pleural alteration.
- 1: In the ultrasound window, vertical artifacts represent less than 1/3 of the length of the pleural line.
- 2: In the ultrasound window, vertical artifacts represent 1/3 to 2/3 of the length of the pleural line.
- 3: In the ultrasound window, vertical artifacts represent more than 2/3 of the length of the pleural line.
- 4: Presence of consolidation.

41. How confident are you in your choice? *

Marcar apenas uma oval.

	1	2	3	4	5	
Not	◯	◯	◯	◯	◯	Very confident

42. How do you rate the quality of the image? *

Marcar apenas uma oval.

	1	2	3	4	5	
Low	◯	◯	◯	◯	◯	High quality

43. Note (if any)

____

____

____

____

____

Image 8

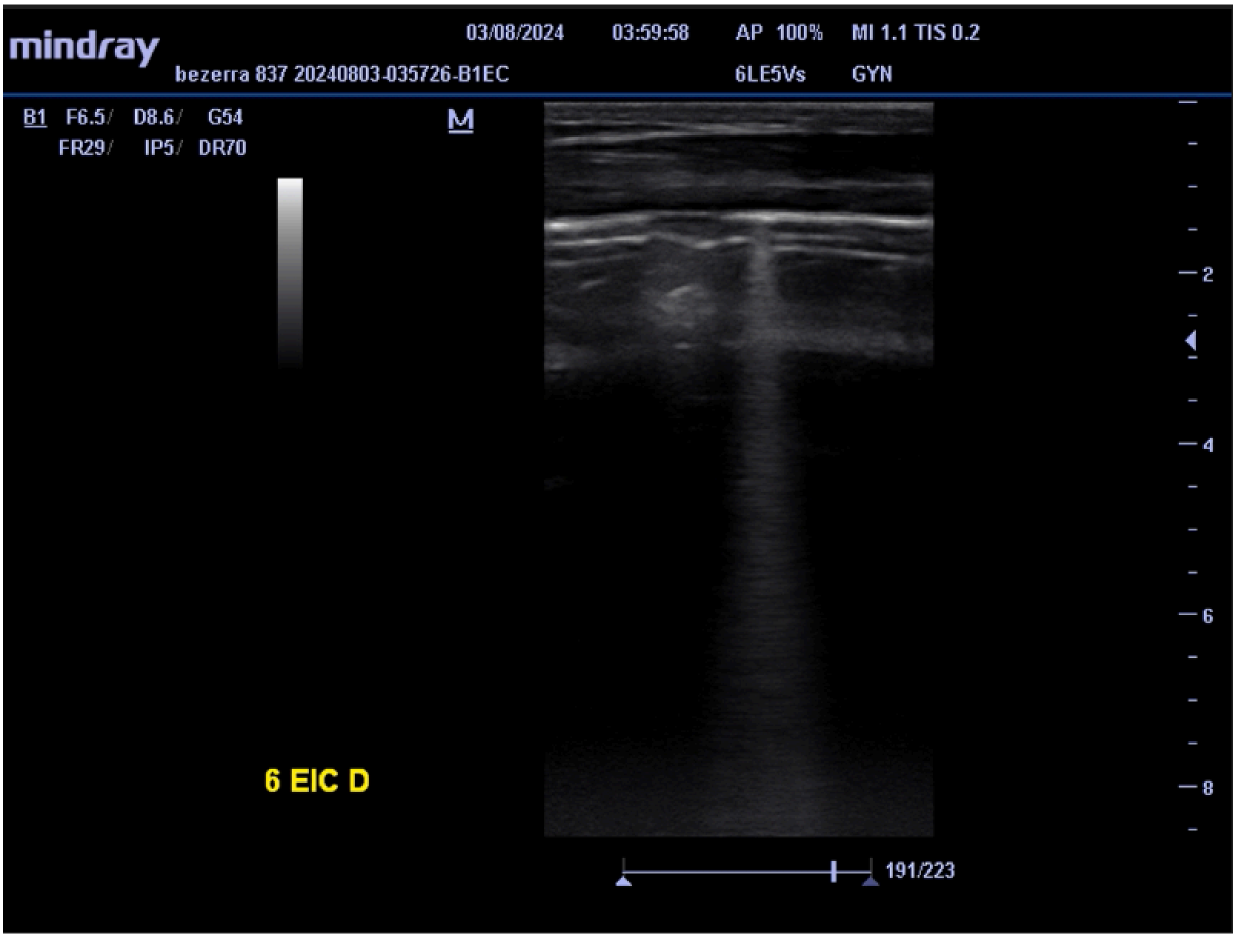

44. What score would you give this image? *

Marcar apenas uma oval.

- 0: Aerated lung with the presence of A-lines, with no pleural alteration.
- 1: In the ultrasound window, vertical artifacts represent less than 1/3 of the length of the pleural line.
- 2: In the ultrasound window, vertical artifacts represent 1/3 to 2/3 of the length of the pleural line.
- 3: In the ultrasound window, vertical artifacts represent more than 2/3 of the length of the pleural line.
- 4: Presence of consolidation and pleural incongruity.

45. How confident are you in your choice? *

Marcar apenas uma oval.

	1	2	3	4	5	
Not	◯	◯	◯	◯	◯	Very confident

46. How do you rate the quality of the image? *

Marcar apenas uma oval.

	1	2	3	4	5	
Low	◯	◯	◯	◯	◯	High quality

47. Note (if any)

Image 9

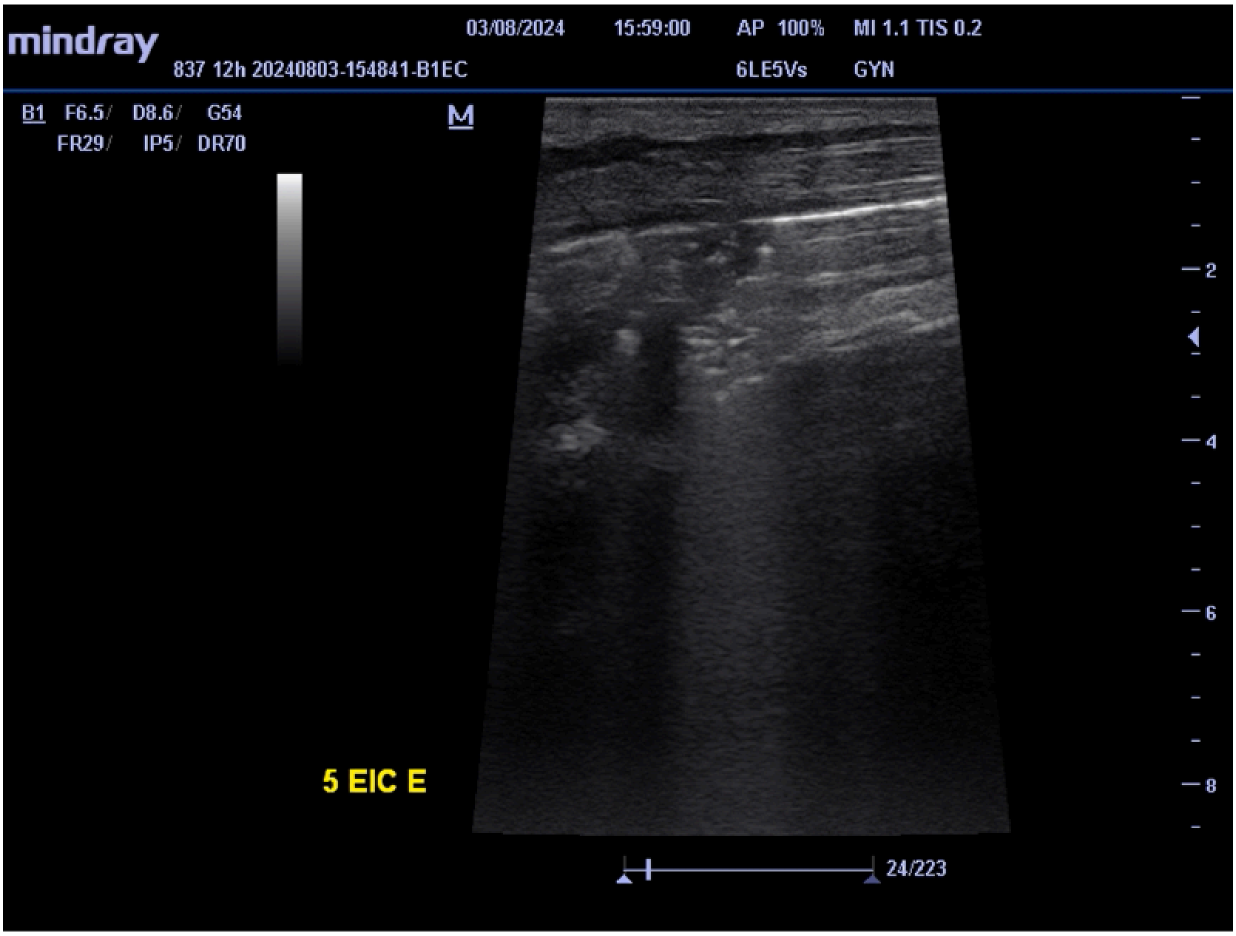

48. What score would you give this image? *

Marcar apenas uma oval.

- 0: Aerated lung with the presence of A-lines, with no pleural alteration.
- 1: In the ultrasound window, vertical artifacts represent less than 1/3 of the length of the pleural line.
- 2: In the ultrasound window, vertical artifacts represent 1/3 to 2/3 of the length of the pleural line.
- 3: In the ultrasound window, vertical artifacts represent more than 2/3 of the length of the pleural line.
- 4: Presence of consolidation.

49. How confident are you in your choice? *

Marcar apenas uma oval.

	1	2	3	4	5	
Not	◯	◯	◯	◯	◯	Very confident

50. How do you rate the quality of the image? *

Marcar apenas uma oval.

	1	2	3	4	5	
Low	◯	◯	◯	◯	◯	High quality

51. Note (if any)

____
____
____
____
____

Image 10

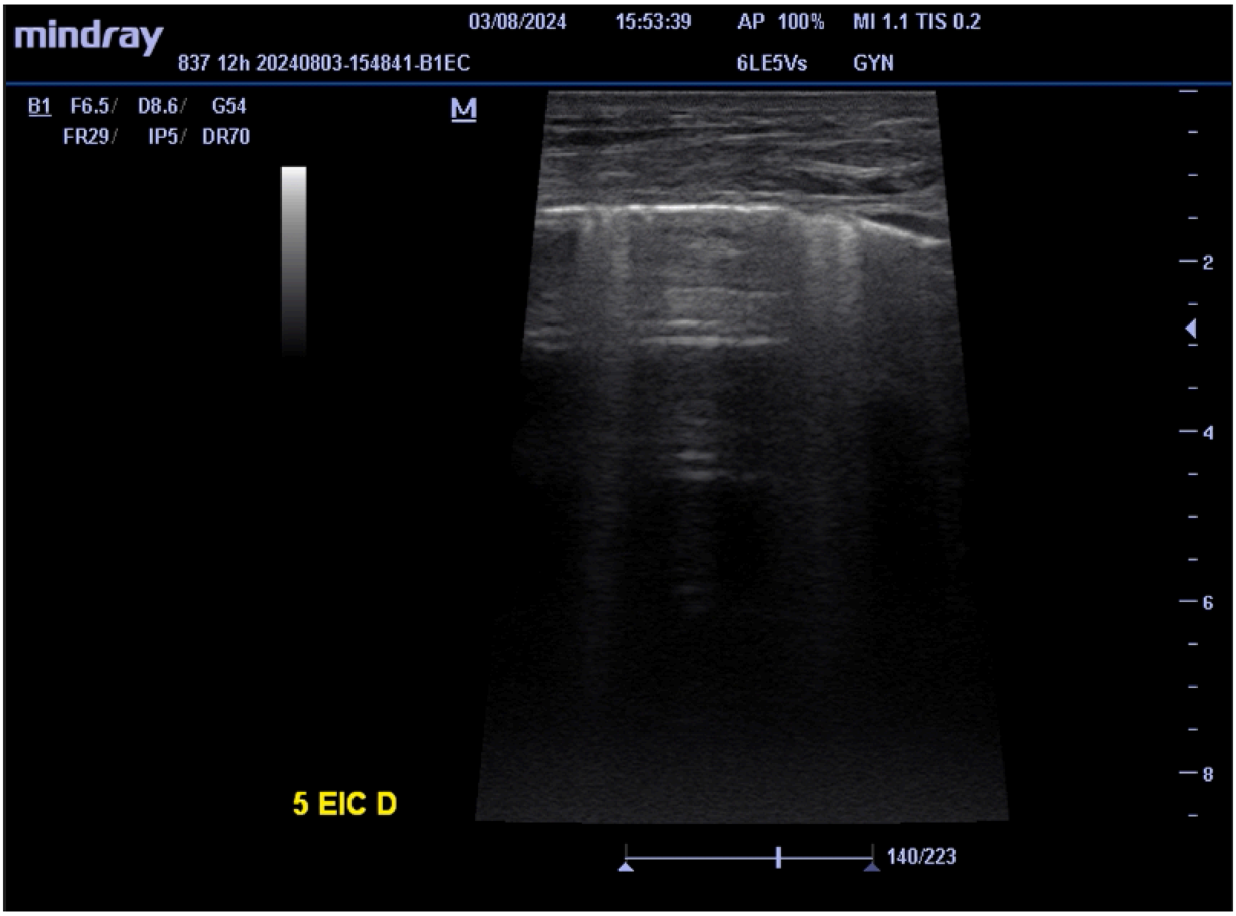

52. What score would you give this image? *

Marcar apenas uma oval.

- 0: Aerated lung with the presence of A-lines, with no pleural alteration.
- 1: In the ultrasound window, vertical artifacts represent less than 1/3 of the length of the pleural line.
- 2: In the ultrasound window, vertical artifacts represent 1/3 to 2/3 of the length of the pleural line.
- 3: In the ultrasound window, vertical artifacts represent more than 2/3 of the length of the pleural line.
- 4: Presence of consolidation.

53. How confident are you in your choice? *

Marcar apenas uma oval.

	1	2	3	4	5	
Not	◯	◯	◯	◯	◯	Very confident

54. How do you rate the quality of the image? *

Marcar apenas uma oval.

	1	2	3	4	5	
Low	◯	◯	◯	◯	◯	High quality

55. Note (if any)

____

____

____

____

____

Image 11

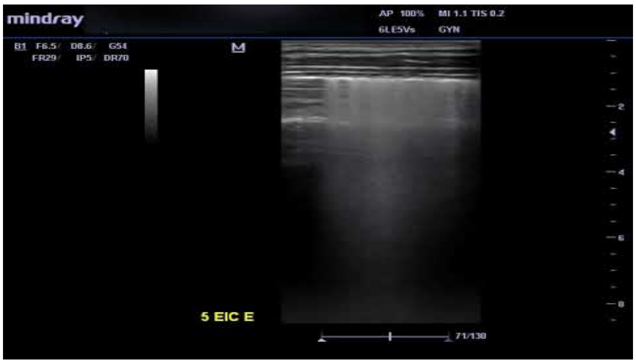

http://youtube.com/watch?v=aghNZR-SGbc

56. What score would you give this image? *

Marcar apenas uma oval.

- ( ) 0: Aerated lung with the presence of A-lines, with no pleural alteration.
- ( ) 1: In the ultrasound window, vertical artifacts represent less than 1/3 of the length of the pleural line.
- ( ) 2: In the ultrasound window, vertical artifacts represent 1/3 to 2/3 of the length of the pleural line.
- ( ) 3: In the ultrasound window, vertical artifacts represent more than 2/3 of the length of the pleural line.
- ( ) 4: Presence of consolidation.

57. How confident are you in your choice? *

Marcar apenas uma oval.

	1	2	3	4	5	
Not	( )	( )	( )	( )	( )	Very confident

58. How do you rate the quality of the video? *

Marcar apenas uma oval.

	1	2	3	4	5	
Low	( )	( )	( )	( )	( )	High quality

59. Note (if any)

Image 12

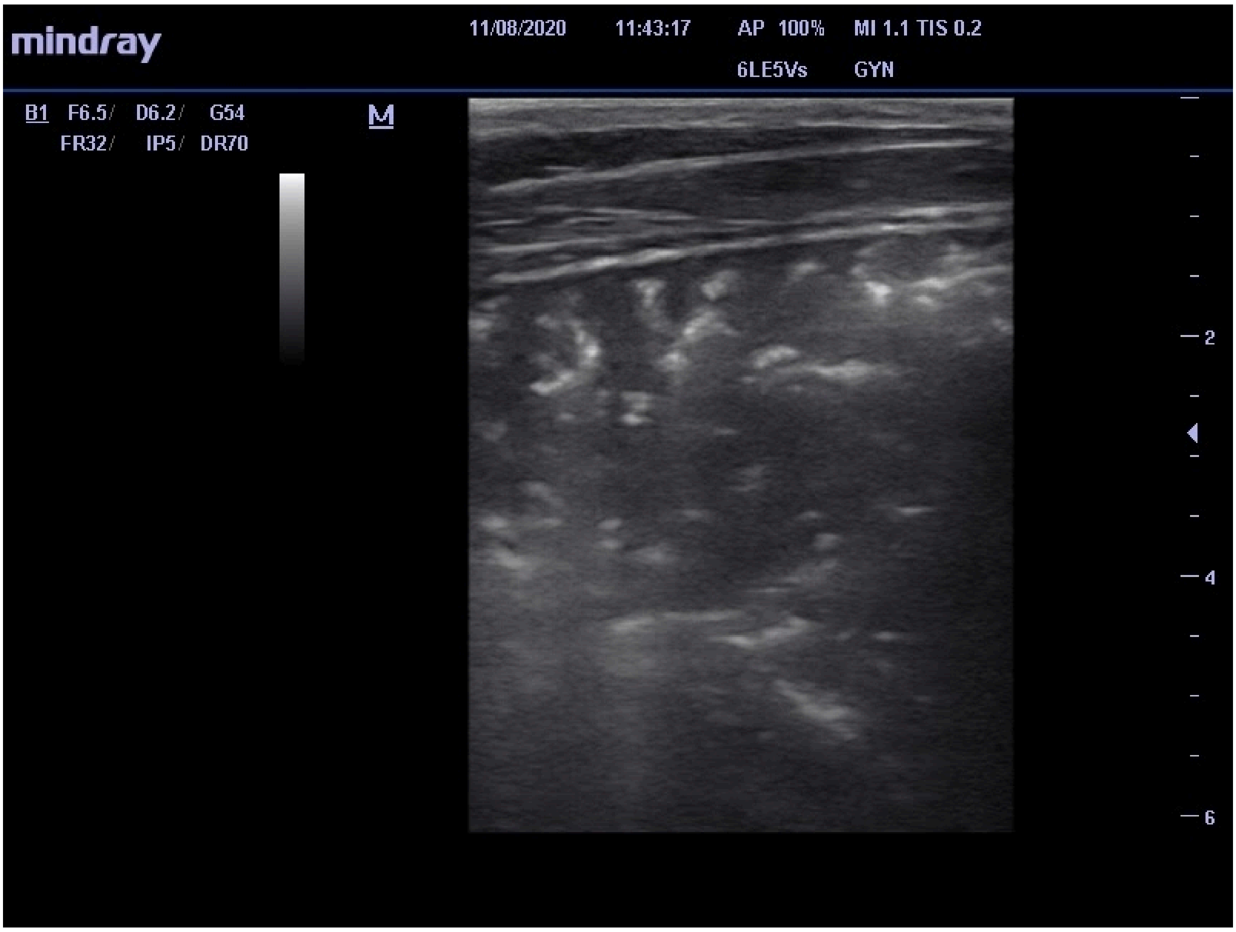

60. What score would you give this image? *

Marcar apenas uma oval.

- 0: Aerated lung with the presence of A-lines, with no pleural alteration.
- 1: In the ultrasound window, vertical artifacts represent less than 1/3 of the length of the pleural line.
- 2: In the ultrasound window, vertical artifacts represent 1/3 to 2/3 of the length of the pleural line.
- 3: In the ultrasound window, vertical artifacts represent more than 2/3 of the length of the pleural line.
- 4: Presence of consolidation.

61. How confident are you in your choice? *

Marcar apenas uma oval.

	1	2	3	4	5	
Not	◯	◯	◯	◯	◯	Very confident

62. How do you rate the quality of the video? *

Marcar apenas uma oval.

	1	2	3	4	5	
Low	◯	◯	◯	◯	◯	High quality

63. Note (if any)

Image 13

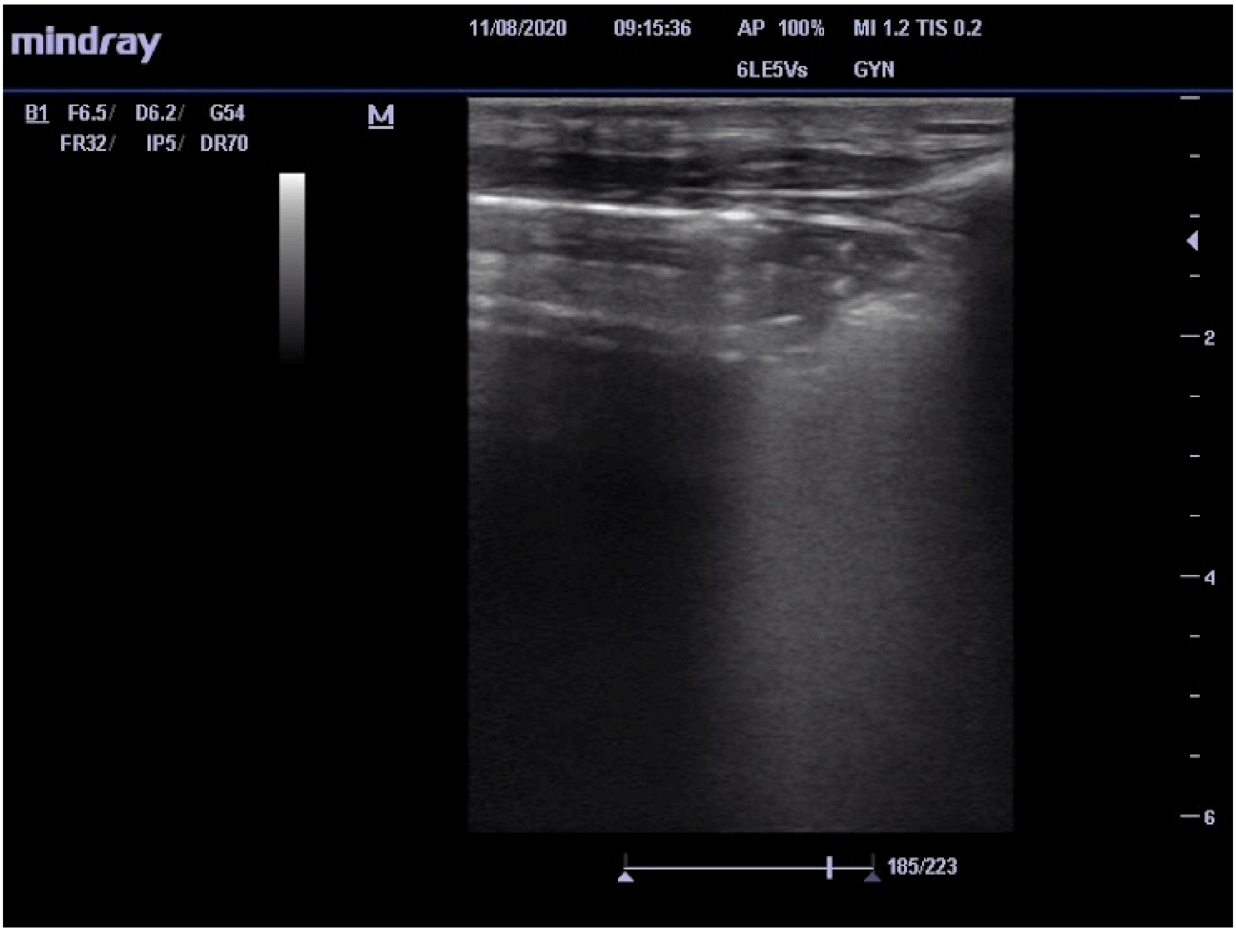

64. What score would you give this image? *

Marcar apenas uma oval.

- 0: Aerated lung with the presence of A-lines, with no pleural alteration.
- 1: In the ultrasound window, vertical artifacts represent less than 1/3 of the length of the pleural line.
- 2: In the ultrasound window, vertical artifacts represent 1/3 to 2/3 of the length of the pleural line.
- 3: In the ultrasound window, vertical artifacts represent more than 2/3 of the length of the pleural line.
- 4: Presence of consolidation.

65. How confident are you in your choice? *

Marcar apenas uma oval.

	1	2	3	4	5	
Not	◯	◯	◯	◯	◯	Very confident

66. How do you rate the quality of the video? *

Marcar apenas uma oval.

	1	2	3	4	5	
Low	◯	◯	◯	◯	◯	High quality

67. Note (if any)

____

____

____

____

____

Image 14

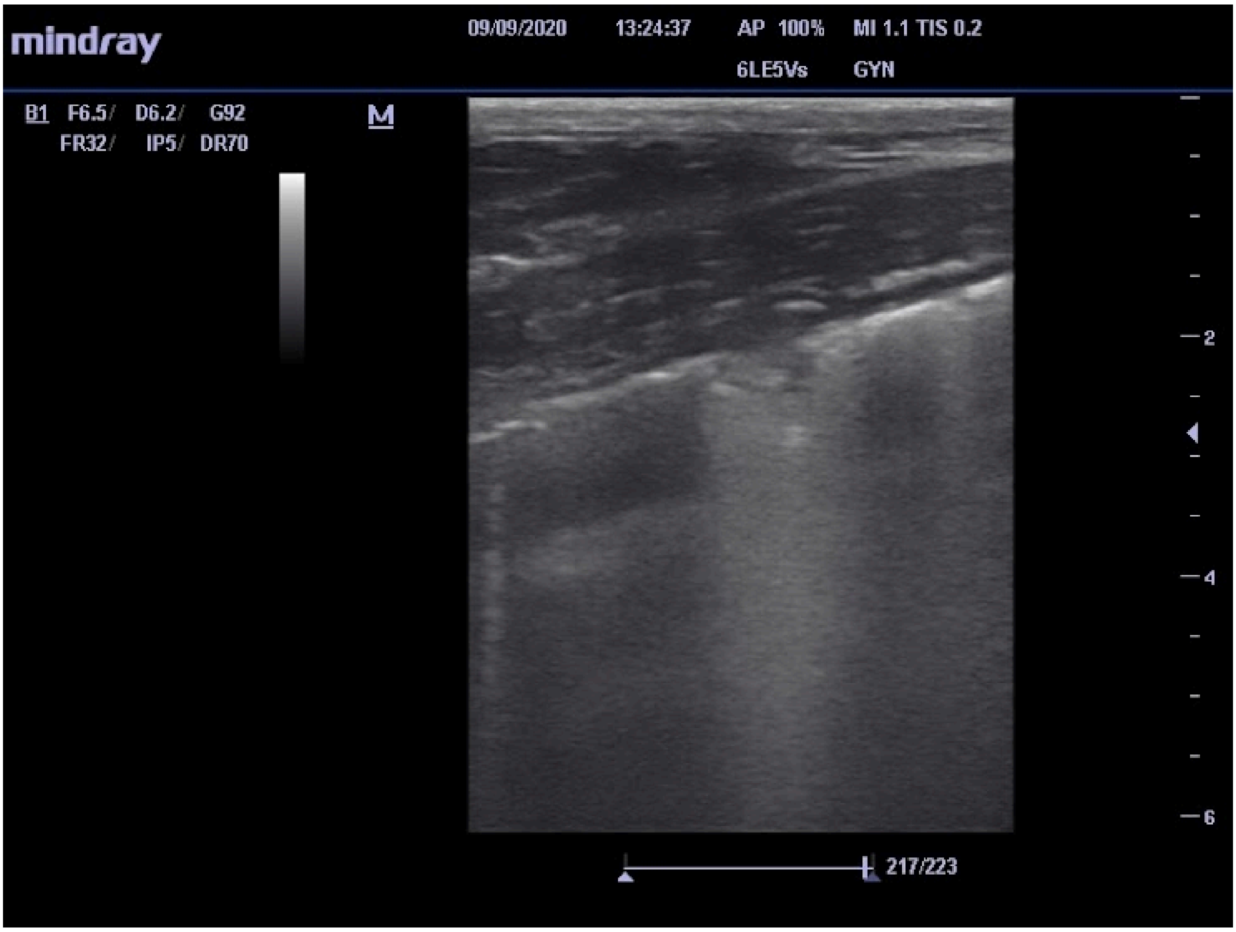

68. What score would you give this image? *

Marcar apenas uma oval.

- 0: Aerated lung with the presence of A-lines, with no pleural alteration.
- 1: In the ultrasound window, vertical artifacts represent less than 1/3 of the length of the pleural line.
- 2: In the ultrasound window, vertical artifacts represent 1/3 to 2/3 of the length of the pleural line.
- 3: In the ultrasound window, vertical artifacts represent more than 2/3 of the length of the pleural line.
- 4: Presence of consolidation.

69. How confident are you in your choice? *

Marcar apenas uma oval.

	1	2	3	4	5	
Not	◯	◯	◯	◯	◯	Very confident

70. How do you rate the quality of the video? *

Marcar apenas uma oval.

	1	2	3	4	5	
Low	◯	◯	◯	◯	◯	High quality

71. Note (if any)

Image 15

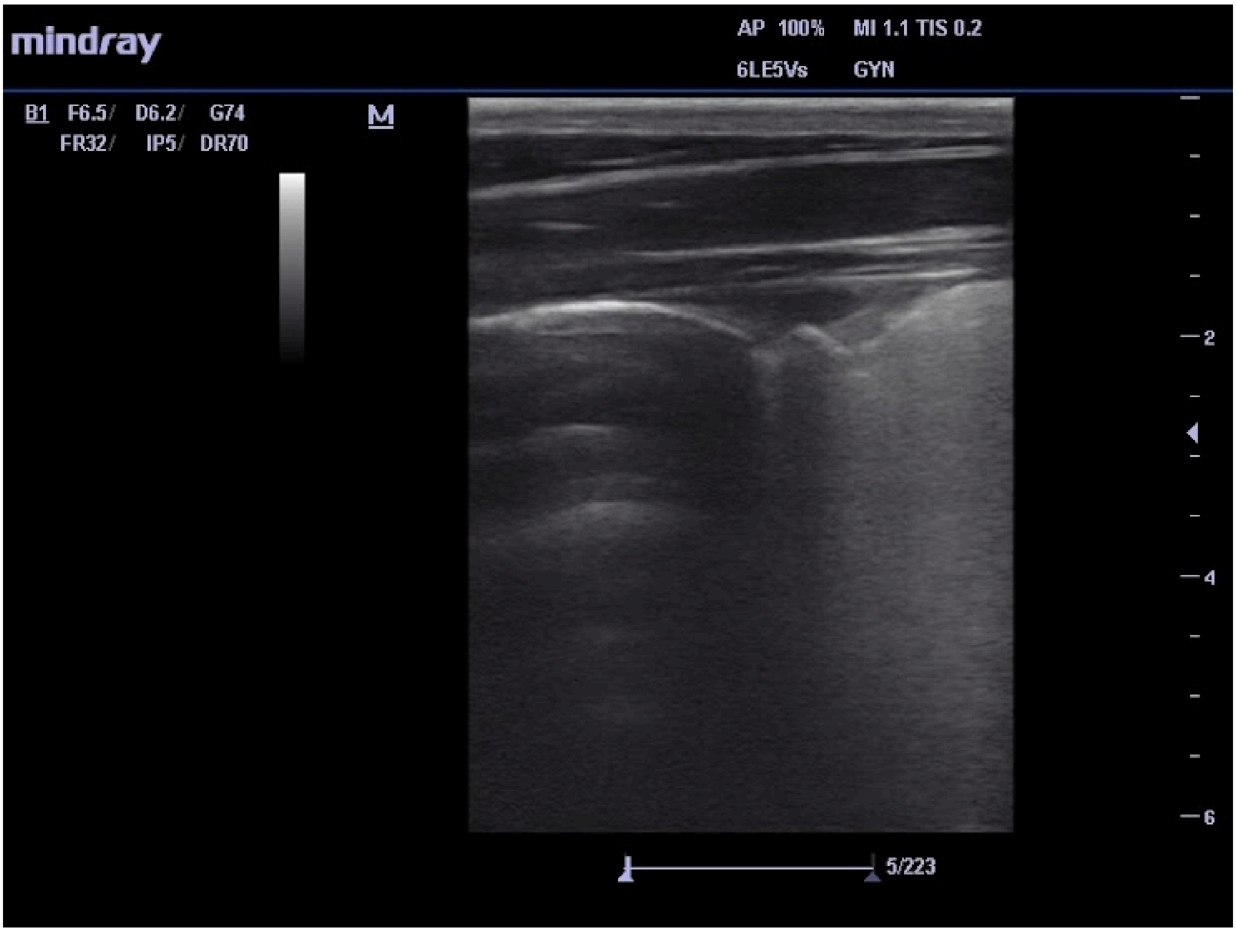

72. What score would you give this image? *

Marcar apenas uma oval.

- 0: Aerated lung with the presence of A-lines, with no pleural alteration.
- 1: In the ultrasound window, vertical artifacts represent less than 1/3 of the length of the pleural line.
- 2: In the ultrasound window, vertical artifacts represent 1/3 to 2/3 of the length of the pleural line.
- 3: In the ultrasound window, vertical artifacts represent more than 2/3 of the length of the pleural line.
- 4: Presence of consolidation.

73. How confident are you in your choice? *

Marcar apenas uma oval.

	1	2	3	4	5	
Not	◯	◯	◯	◯	◯	Very confident

74. How do you rate the quality of the video? *

Marcar apenas uma oval.

	1	2	3	4	5	
Low	◯	◯	◯	◯	◯	High quality

75. Note (if any)

____
____
____
____
____

Image 16

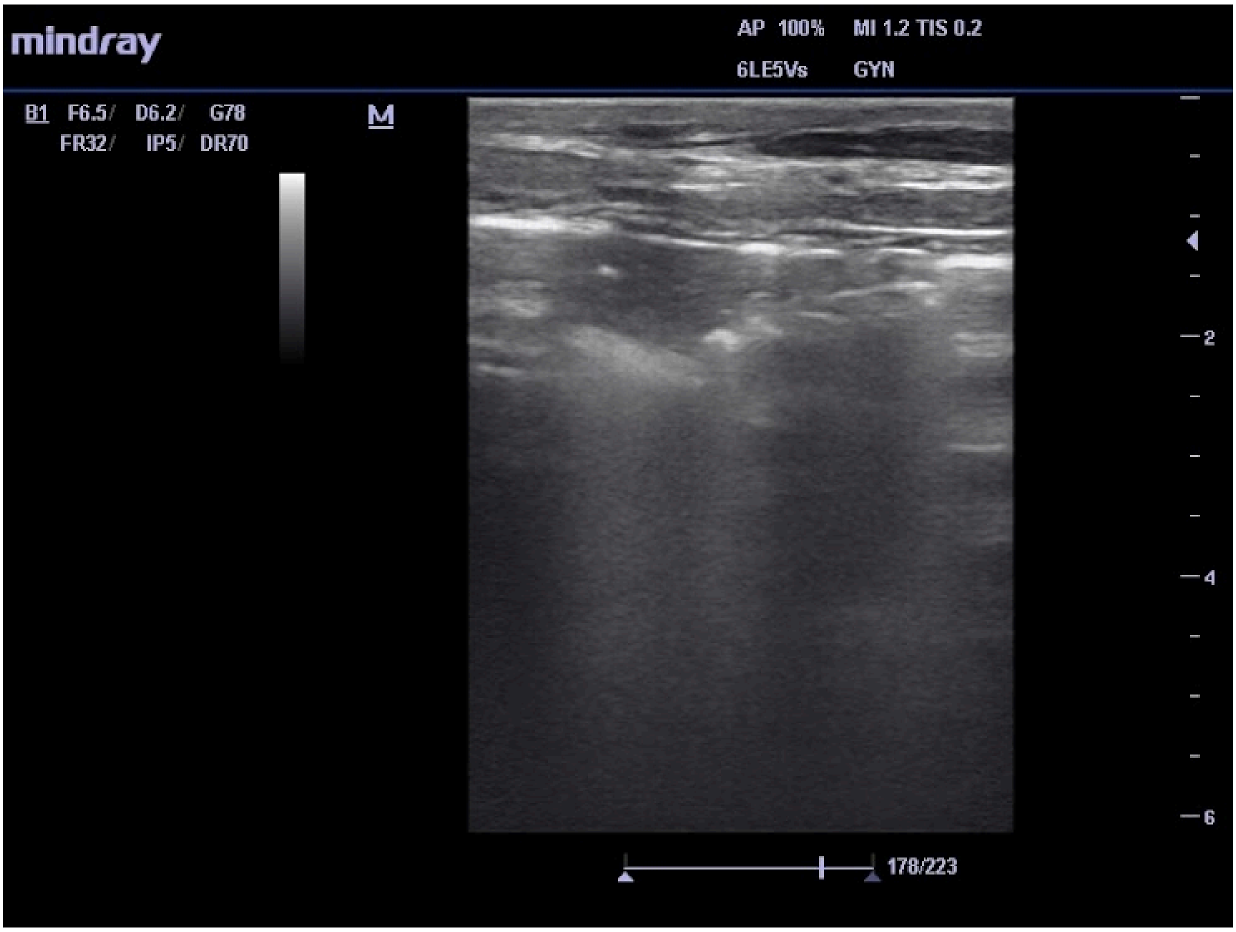

76. What score would you give this image? *

Marcar apenas uma oval.

- 0: Aerated lung with the presence of A-lines, with no pleural alteration.
- 1: In the ultrasound window, vertical artifacts represent less than 1/3 of the length of the pleural line.
- 2: In the ultrasound window, vertical artifacts represent 1/3 to 2/3 of the length of the pleural line.
- 3: In the ultrasound window, vertical artifacts represent more than 2/3 of the length of the pleural line.
- 4: Presence of consolidation.

77. How confident are you in your choice? *

Marcar apenas uma oval.

	1	2	3	4	5	
Not	◯	◯	◯	◯	◯	Very confident

78. How do you rate the quality of the video? *

Marcar apenas uma oval.

	1	2	3	4	5	
Low	◯	◯	◯	◯	◯	High quality

79. Note (if any)

____

____

____

____

____

Image 17

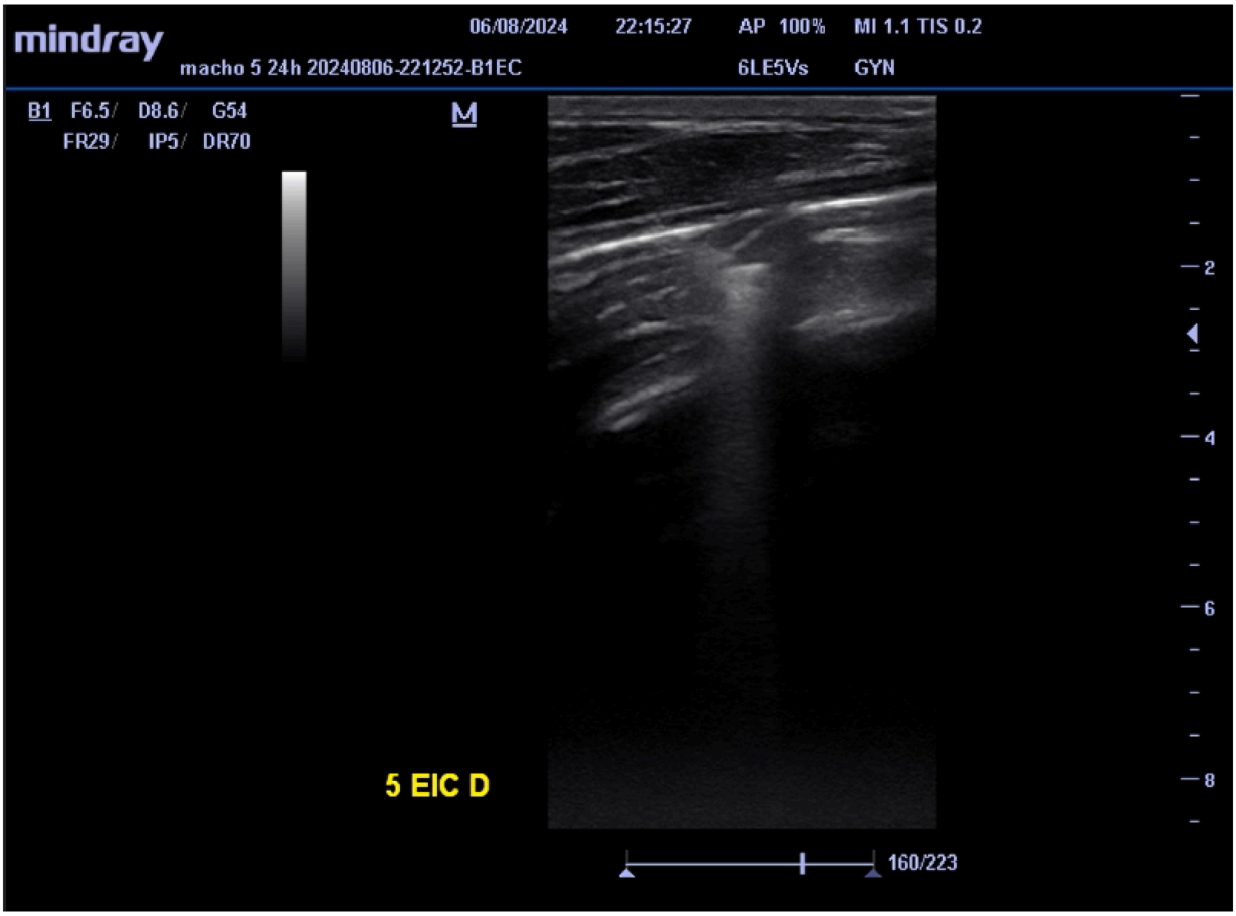

80. What score would you give this image? *

Marcar apenas uma oval.

- 0: Aerated lung with the presence of A-lines, with no pleural alteration.
- 1: In the ultrasound window, vertical artifacts represent less than 1/3 of the length of the pleural line.
- 2: In the ultrasound window, vertical artifacts represent 1/3 to 2/3 of the length of the pleural line.
- 3: In the ultrasound window, vertical artifacts represent more than 2/3 of the length of the pleural line.
- 4: Presence of consolidation.

81. How confident are you in your choice? *

Marcar apenas uma oval.

	1	2	3	4	5	
Not	◯	◯	◯	◯	◯	Very confident

82. How do you rate the quality of the image? *

Marcar apenas uma oval.

	1	2	3	4	5	
Low	◯	◯	◯	◯	◯	High quality

83. Note (if any)

____

____

____

____

____

Image 18

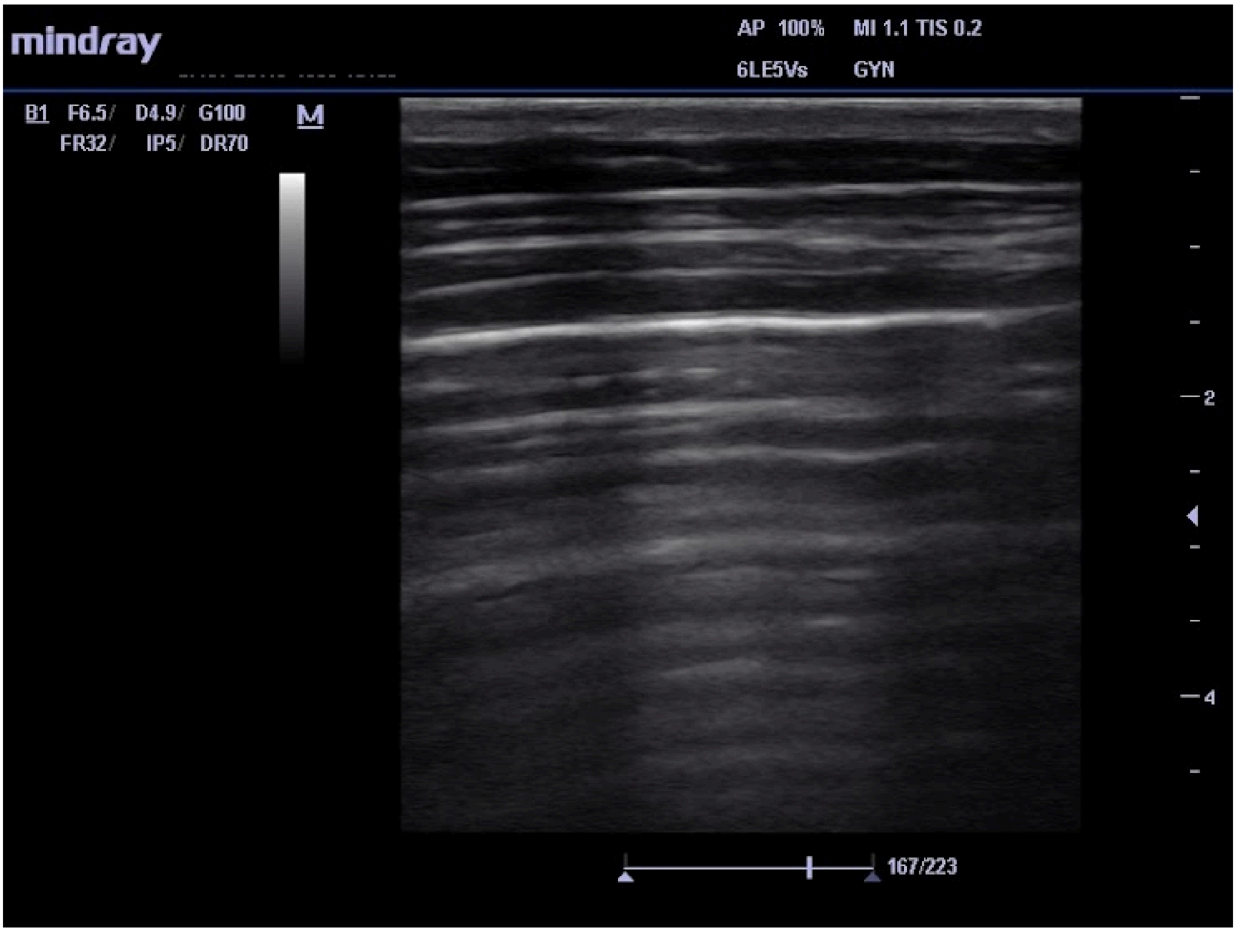

84. What score would you give this image? *

Marcar apenas uma oval.

- 0: Aerated lung with the presence of A-lines, with no pleural alteration.
- 1: In the ultrasound window, vertical artifacts represent less than 1/3 of the length of the pleural line.
- 2: In the ultrasound window, vertical artifacts represent 1/3 to 2/3 of the length of the pleural line.
- 3: In the ultrasound window, vertical artifacts represent more than 2/3 of the length of the pleural line.
- 4: Presence of consolidation.

85. How confident are you in your choice? *

Marcar apenas uma oval.

	1	2	3	4	5	
Not	◯	◯	◯	◯	◯	Very confident

86. How do you rate the quality of the image? *

Marcar apenas uma oval.

	1	2	3	4	5	
Low	◯	◯	◯	◯	◯	High quality

87. Note (if any)

Image 19

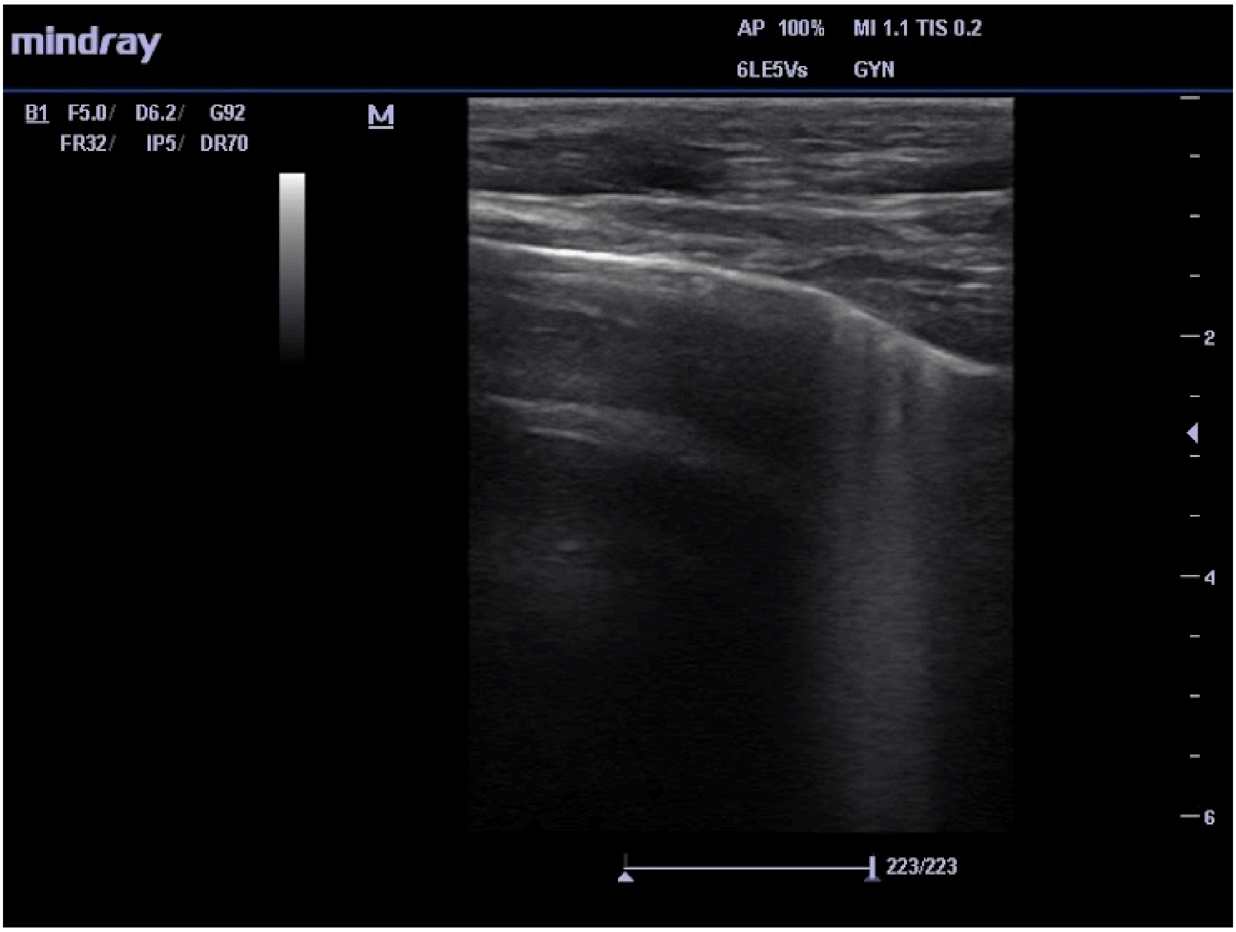

88. What score would you give this image? *

Marcar apenas uma oval.

- 0: Aerated lung with the presence of A-lines, with no pleural alteration.
- 1: In the ultrasound window, vertical artifacts represent less than 1/3 of the length of the pleural line.
- 2: In the ultrasound window, vertical artifacts represent 1/3 to 2/3 of the length of the pleural line.
- 3: In the ultrasound window, vertical artifacts represent more than 2/3 of the length of the pleural line.
- 4: Presence of consolidation.

89. How confident are you in your choice? *

Marcar apenas uma oval.

	1	2	3	4	5	
Not	◯	◯	◯	◯	◯	Very confident

90. How do you rate the quality of the image? *

Marcar apenas uma oval.

	1	2	3	4	5	
Low	◯	◯	◯	◯	◯	High quality

91. Note (if any)

____
____
____
____
____

92. **Image 20**

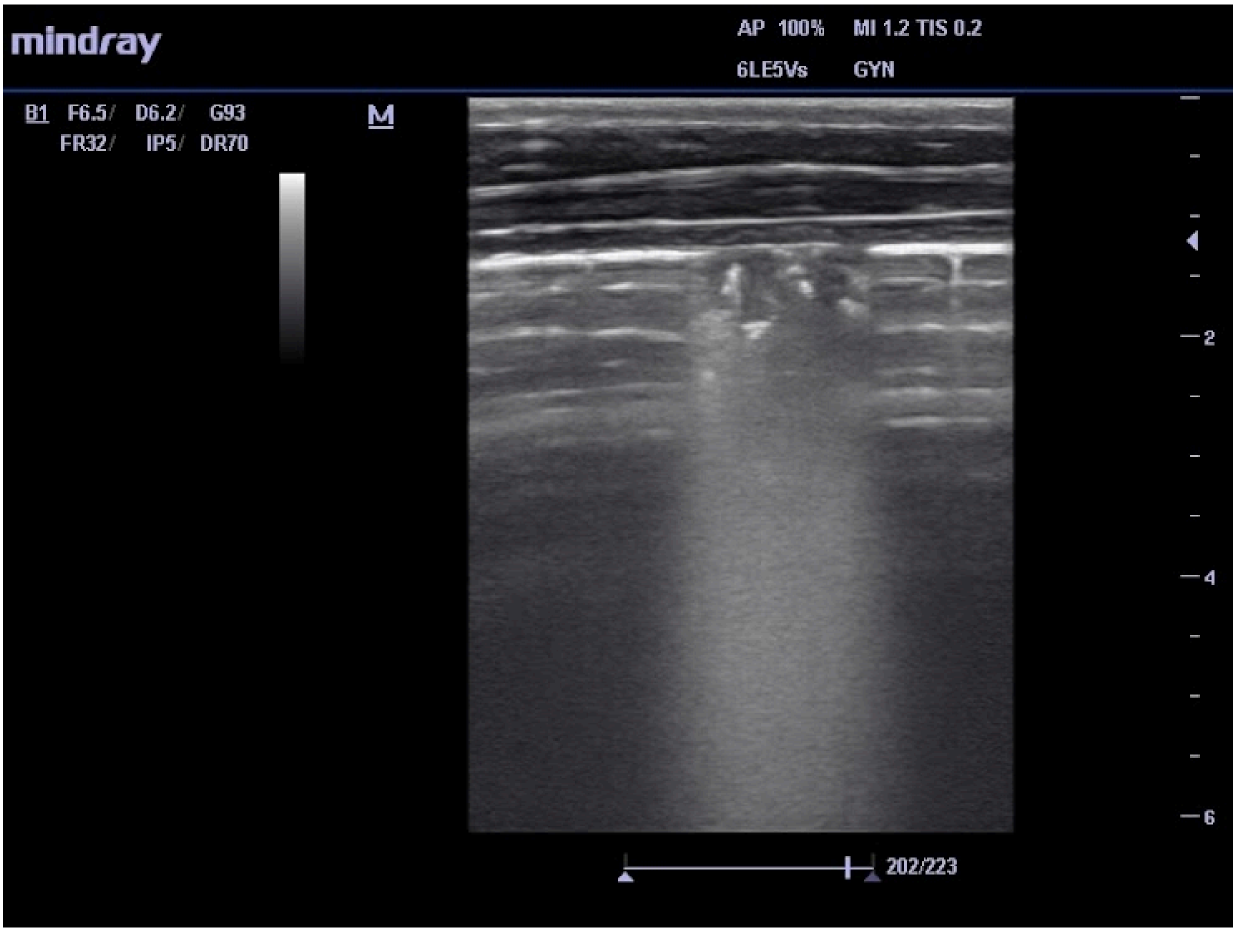

Marcar apenas uma oval.

- 0: Aerated lung with the presence of A-lines, with no pleural alteration.
- 1: In the ultrasound window, vertical artifacts represent less than 1/3 of the length of the pleural line.
- 2: In the ultrasound window, vertical artifacts represent 1/3 to 2/3 of the length of the pleural line.
- 3: In the ultrasound window, vertical artifacts represent more than 2/3 of the length of the pleural line.
- 4: Presence of consolidation.

93. How confident are you in your choice? *

Marcar apenas uma oval.

	1	2	3	4	5	
Not						Very confident

94. How do you rate the quality of the image? *

Marcar apenas uma oval.

	1	2	3	4	5	
Low	◯	◯	◯	◯	◯	High quality

95. Note (if any)

Image 21

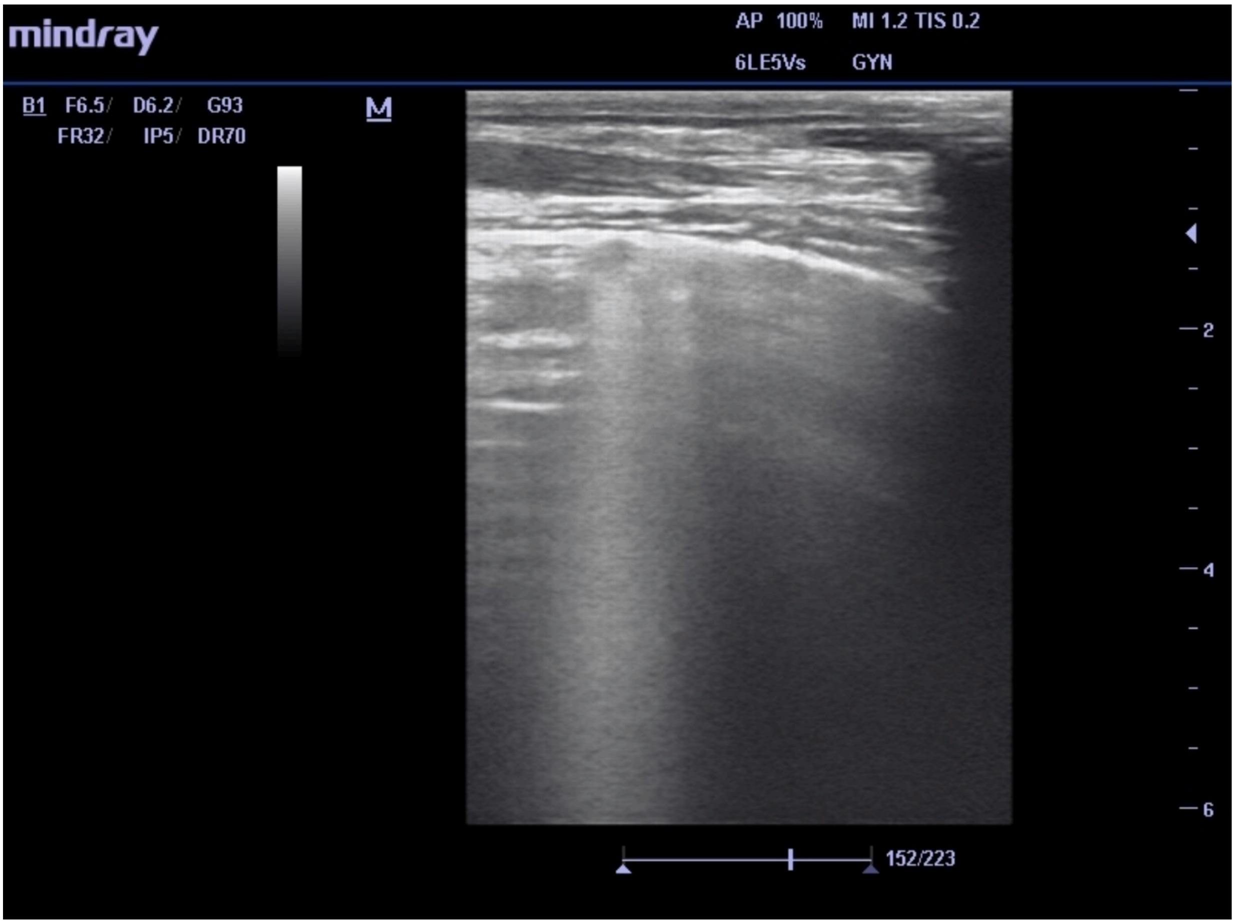

96. What score would you give this image? *

Marcar apenas uma oval.

- 0: Aerated lung with the presence of A-lines, with no pleural alteration.
- 1: In the ultrasound window, vertical artifacts represent less than 1/3 of the length of the pleural line.
- 2: In the ultrasound window, vertical artifacts represent 1/3 to 2/3 of the length of the pleural line.
- 3: In the ultrasound window, vertical artifacts represent more than 2/3 of the length of the pleural line.
- 4: Presence of consolidation.

97. How confident are you in your choice? *

Marcar apenas uma oval.

	1	2	3	4	5	
Not						Very confident

98. How do you rate the quality of the image? *

Marcar apenas uma oval.

	1	2	3	4	5	
Low						High quality

99. Note (if any)

Image 22

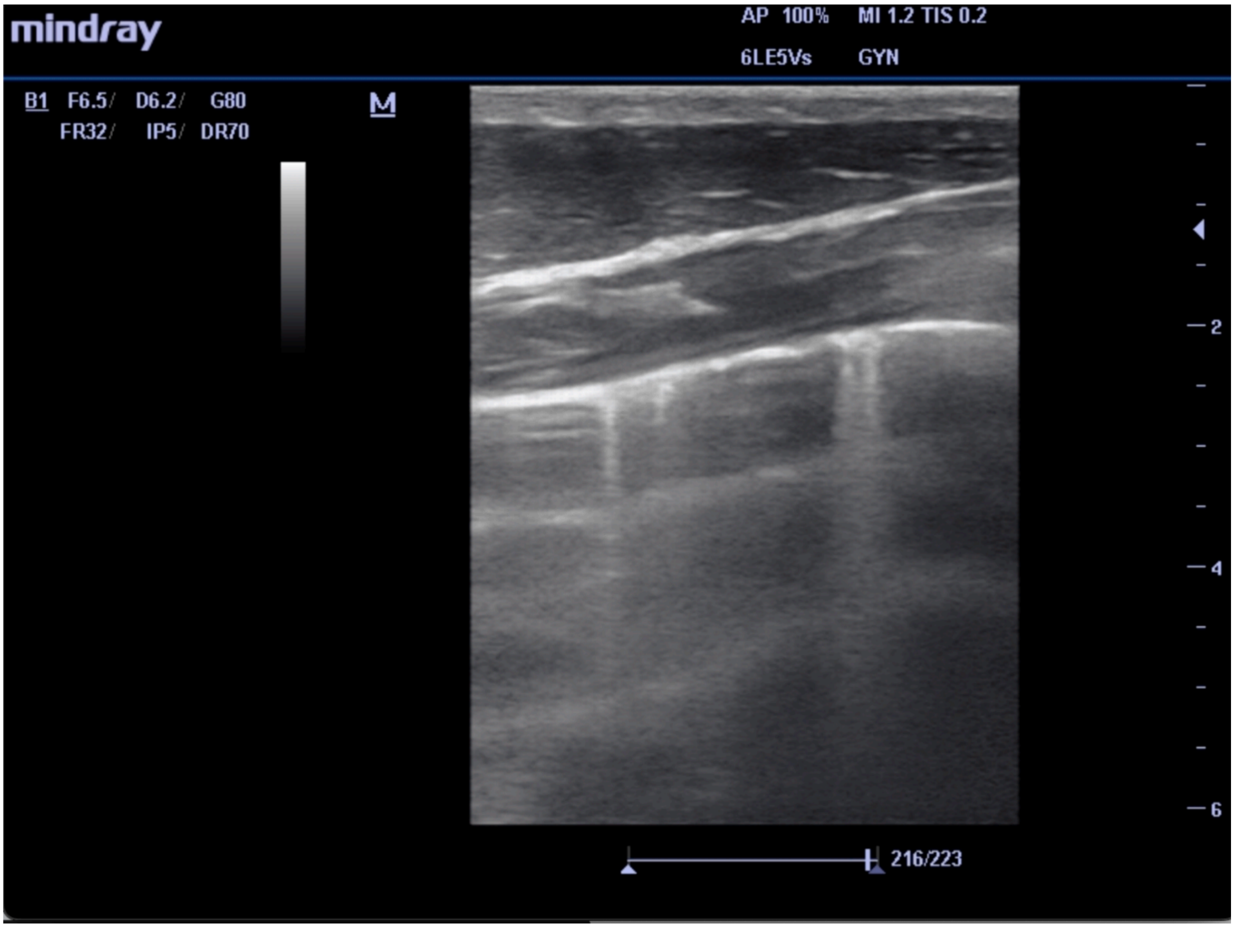

100. What score would you give this image? *

Marcar apenas uma oval.

- 0: Aerated lung with the presence of A-lines, with no pleural alteration.
- 1: In the ultrasound window, vertical artifacts represent less than 1/3 of the length of the pleural line.
- 2: In the ultrasound window, vertical artifacts represent 1/3 to 2/3 of the length of the pleural line.
- 3: In the ultrasound window, vertical artifacts represent more than 2/3 of the length of the pleural line.
- 4: Presence of consolidation.

101. How confident are you in your choice? *

Marcar apenas uma oval.

	1	2	3	4	5	
Not	◯	◯	◯	◯	◯	Very confident

102. How do you rate the quality of the image? *

Marcar apenas uma oval.

	1	2	3	4	5	
Low	◯	◯	◯	◯	◯	High quality

103. Note (if any)

____

____

____

____

____

Image 23

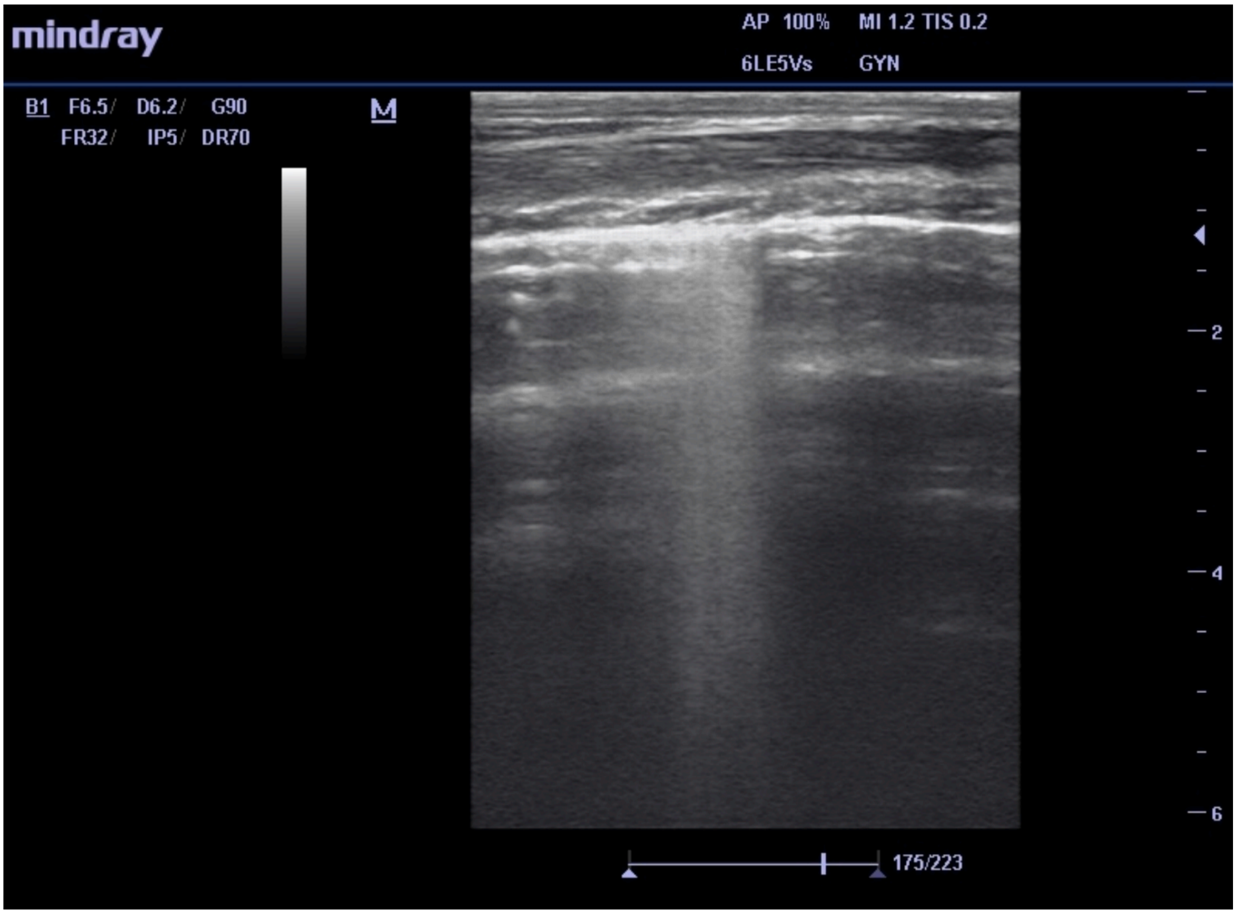

104. What score would you give this image? *

Marcar apenas uma oval.

- 0: Aerated lung with the presence of A-lines, with no pleural alteration.
- 1: In the ultrasound window, vertical artifacts represent less than 1/3 of the length of the pleural line.
- 2: In the ultrasound window, vertical artifacts represent 1/3 to 2/3 of the length of the pleural line.
- 3: In the ultrasound window, vertical artifacts represent more than 2/3 of the length of the pleural line.
- 4: Presence of consolidation.

105. How confident are you in your choice? *

Marcar apenas uma oval.

	1	2	3	4	5	
Not	◯	◯	◯	◯	◯	Very confident

106. How do you rate the quality of the image? *

Marcar apenas uma oval.

	1	2	3	4	5	
Low	◯	◯	◯	◯	◯	High quality

107. Note (if any)

____

____

____

____

____

Image 24

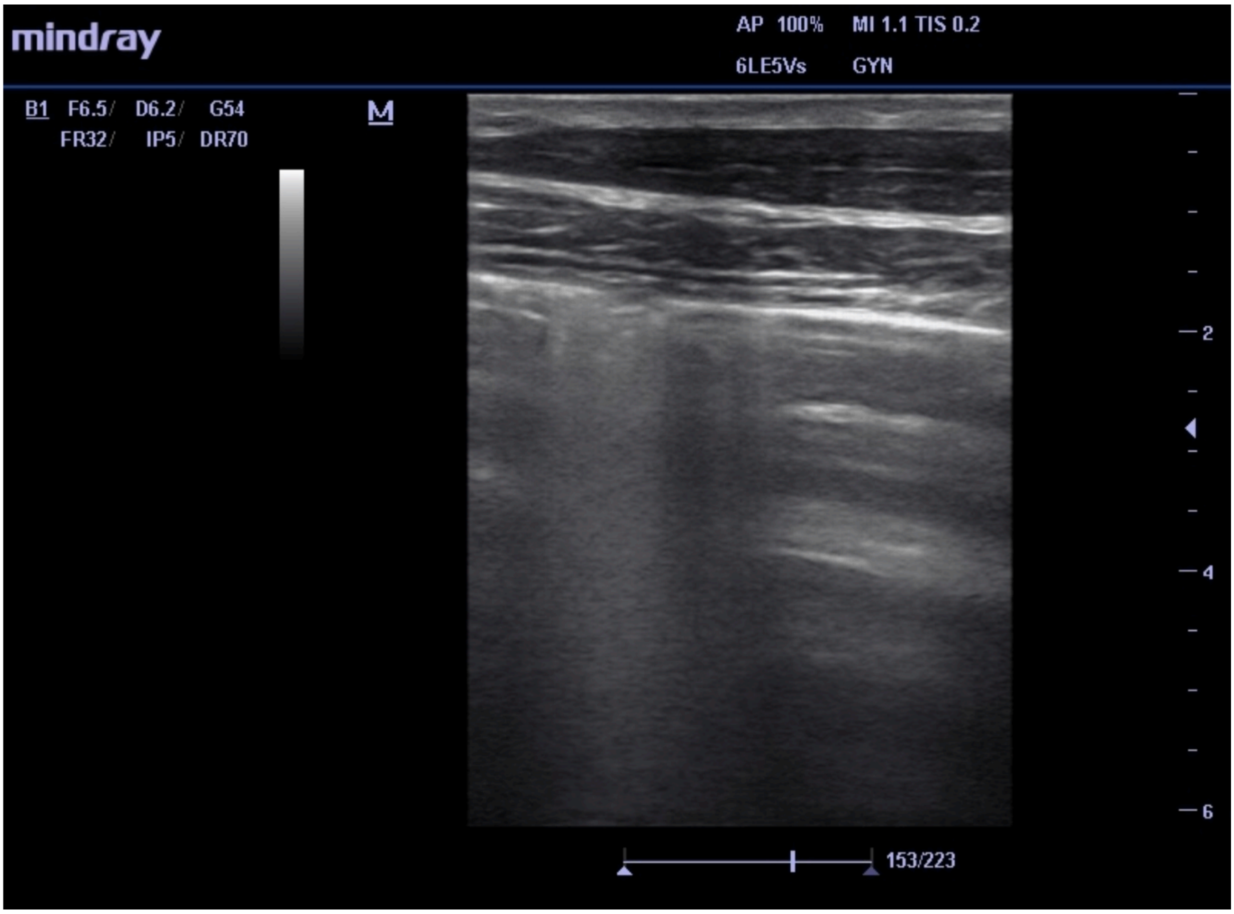

108. What score would you give this image? *

Marcar apenas uma oval.

- 0: Aerated lung with the presence of A-lines, with no pleural alteration.
- 1: In the ultrasound window, vertical artifacts represent less than 1/3 of the length of the pleural line.
- 2: In the ultrasound window, vertical artifacts represent 1/3 to 2/3 of the length of the pleural line.
- 3: In the ultrasound window, vertical artifacts represent more than 2/3 of the length of the pleural line.
- 4: Presence of consolidation.

109. How confident are you in your choice? *

Marcar apenas uma oval.

	1	2	3	4	5	
Not	◯	◯	◯	◯	◯	Very confident

110. How do you rate the quality of the image? *

Marcar apenas uma oval.

	1	2	3	4	5	
Low	◯	◯	◯	◯	◯	High quality

111. Note (if any)

Image 25

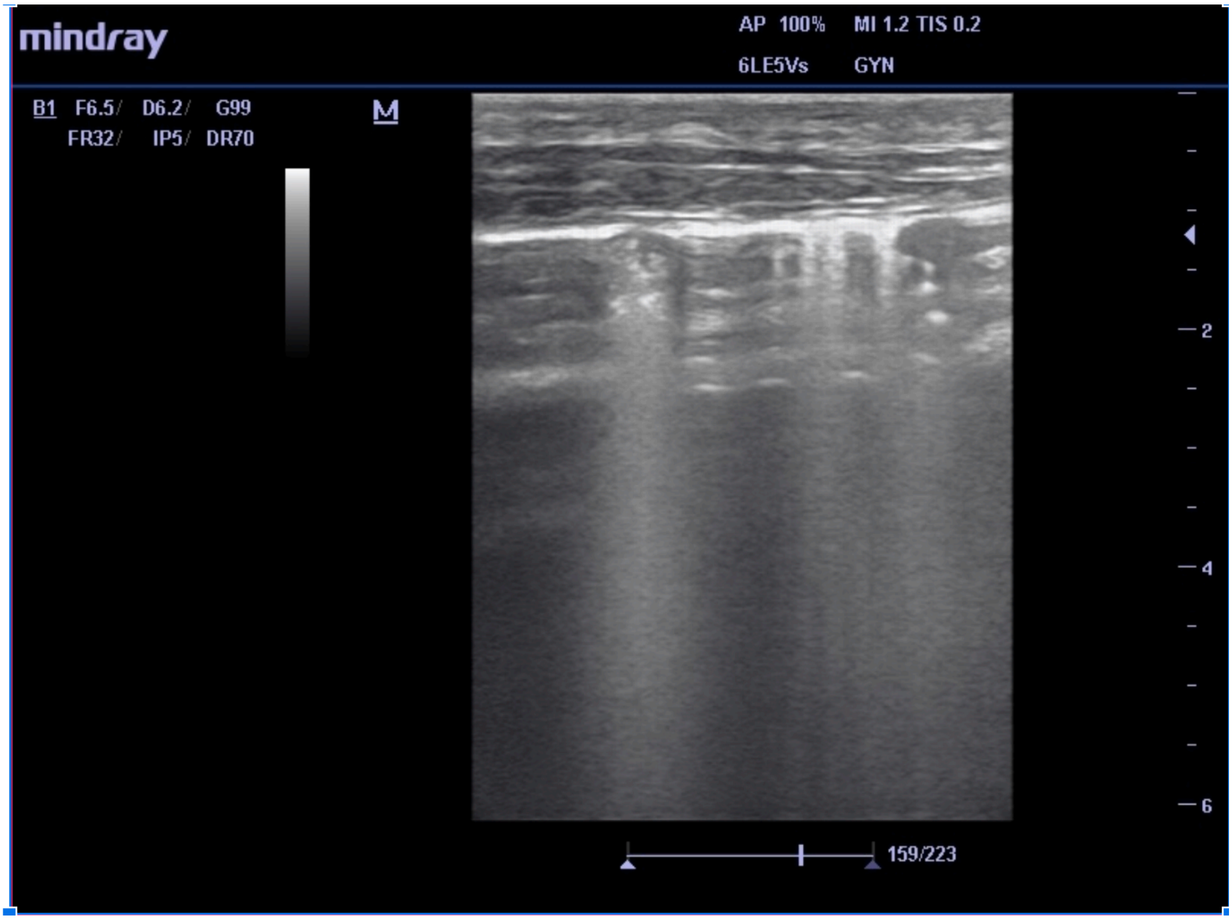

112. What score would you give this image? *

Marcar apenas uma oval.

- 0: Aerated lung with the presence of A-lines, with no pleural alteration.
- 1: In the ultrasound window, vertical artifacts represent less than 1/3 of the length of the pleural line.
- 2: In the ultrasound window, vertical artifacts represent 1/3 to 2/3 of the length of the pleural line.
- 3: In the ultrasound window, vertical artifacts represent more than 2/3 of the length of the pleural line.
- 4: Presence of consolidation.

113. How confident are you in your choice? *

Marcar apenas uma oval.

	1	2	3	4	5	
Not	◯	◯	◯	◯	◯	Very confident

114. How do you rate the quality of the image? *

Marcar apenas uma oval.

	1	2	3	4	5	
Low	◯	◯	◯	◯	◯	High quality

115. Note (if any)

Video 1

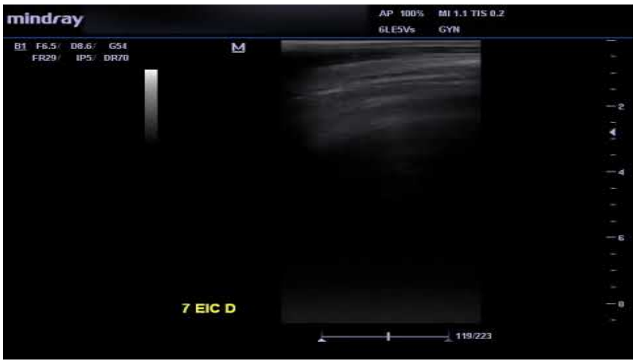

http://youtube.com/watch?v=2vt0pR6bZxw

116. What score would you give this video? *

Marcar apenas uma oval.

- 0: Aerated lung with the presence of A-lines, with no pleural alteration.
- 1: In the ultrasound window, vertical artifacts represent less than 1/3 of the length of the pleural line.
- 2: In the ultrasound window, vertical artifacts represent 1/3 to 2/3 of the length of the pleural line.
- 3: In the ultrasound window, vertical artifacts represent more than 2/3 of the length of the pleural line.
- 4: Presence of consolidation.

117. How confident are you in your choice? *

Marcar apenas uma oval.

	1	2	3	4	5	
Not	○	○	○	○	○	Very confident

118. How do you rate the quality of the video? *

Marcar apenas uma oval.

	1	2	3	4	5	
Low	○	○	○	○	○	High quality

119. Note (if any)

Video 2

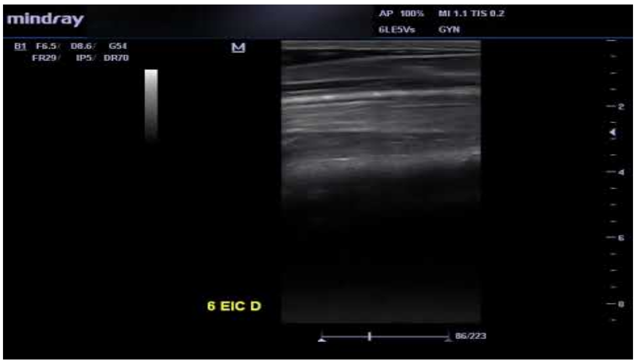

http://youtube.com/watch?v=quhpP0IHskQ

120. What score would you give this video? *

Marcar apenas uma oval.

- ( ) 0: Aerated lung with the presence of A-lines, with no pleural alteration.
- ( ) 1: In the ultrasound window, vertical artifacts represent less than 1/3 of the length of the pleural line.
- ( ) 2: In the ultrasound window, vertical artifacts represent 1/3 to 2/3 of the length of the pleural line.
- ( ) 3: In the ultrasound window, vertical artifacts represent more than 2/3 of the length of the pleural line.
- ( ) 4: Presence of consolidation.

121. How confident are you in your choice? *

Marcar apenas uma oval.

	1	2	3	4	5	
Not	( )	( )	( )	( )	( )	Very confident

122. How do you rate the quality of the video? *

Marcar apenas uma oval.

	1	2	3	4	5	
Low	◯	◯	◯	◯	◯	High quality

123. Note (if any)

____

____

____

____

____

Video 3

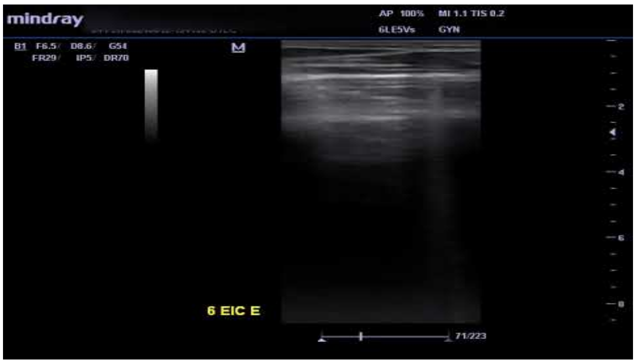

http://youtube.com/watch?v=RHpOLkelX7I

124. What score would you give this video? *

Marcar apenas uma oval.

- 0: Aerated lung with the presence of A-lines, with no pleural alteration.
- 1: In the ultrasound window, vertical artifacts represent less than 1/3 of the length of the pleural line.
- 2: In the ultrasound window, vertical artifacts represent 1/3 to 2/3 of the length of the pleural line.
- 3: In the ultrasound window, vertical artifacts represent more than 2/3 of the length of the pleural line.
- 4: Presence of consolidation.

125. How confident are you in your choice? *

Marcar apenas uma oval.

	1	2	3	4	5	
Not						Very confident

126. How do you rate the quality of the video? *

Marcar apenas uma oval.

	1	2	3	4	5	
Low						High quality

127. Note (if any)

Video 4

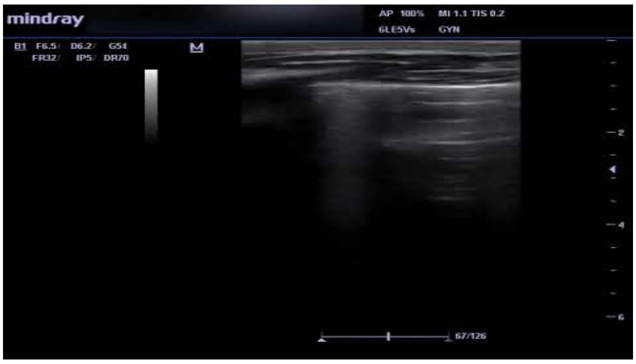

http://youtube.com/watch?v=eN7JWymM7gc

128. What score would you give this video? *

Marcar apenas uma oval.

- ◯ 0: Aerated lung with the presence of A-lines, with no pleural alteration.
- ◯ 1: In the ultrasound window, vertical artifacts represent less than 1/3 of the length of the pleural line.
- ◯ 2: In the ultrasound window, vertical artifacts represent 1/3 to 2/3 of the length of the pleural line.
- ◯ 3: In the ultrasound window, vertical artifacts represent more than 2/3 of the length of the pleural line.
- ◯ 4: Presence of consolidation.

129. How confident are you in your choice? *

Marcar apenas uma oval.

	1	2	3	4	5	
Not	◯	◯	◯	◯	◯	Very confident

130. How do you rate the quality of the video? *

Marcar apenas uma oval.

	1	2	3	4	5	
Low	◯	◯	◯	◯	◯	High quality

131. Note (if any)

____

____

____

____

____

Video 5

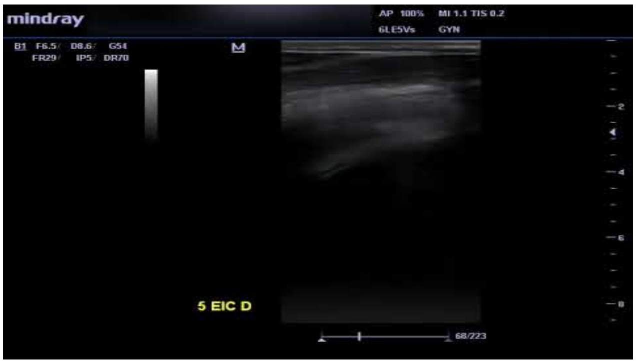

http://youtube.com/watch?v=zIqyvYVj6AY

132. What score would you give this video? *

Marcar apenas uma oval.

◯ 0: Aerated lung with the presence of A-lines, with no pleural alteration.

◯ 1: In the ultrasound window, vertical artifacts represent less than 1/3 of the length of the pleural line.

◯ 2: In the ultrasound window, vertical artifacts represent 1/3 to 2/3 of the length of the pleural line.

◯ 3: In the ultrasound window, vertical artifacts represent more than 2/3 of the length of the pleural line.

◯ 4: Presence of consolidation.

133. How confident are you in your choice? *

Marcar apenas uma oval.

	1	2	3	4	5	
Not	◯	◯	◯	◯	◯	Very confident

134. How do you rate the quality of the video? *

Marcar apenas uma oval.

	1	2	3	4	5	
Low	◯	◯	◯	◯	◯	High quality

135. Note (if any)

____

____

____

____

____

Video 6

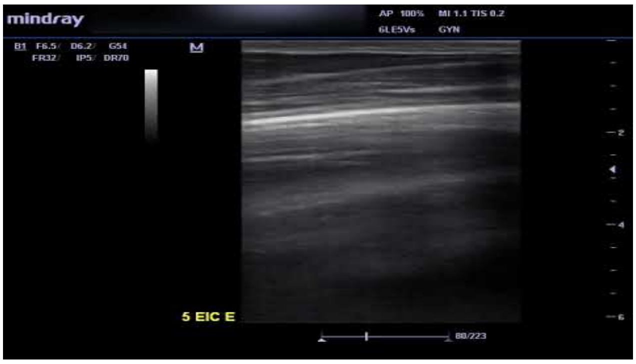

http://youtube.com/watch?v=3ChqK8YFMH4

136. What score would you give this video? *

Marcar apenas uma oval.

- ( ) 0: Aerated lung with the presence of A-lines, with no pleural alteration.
- ( ) 1: In the ultrasound window, vertical artifacts represent less than 1/3 of the length of the pleural line.
- ( ) 2: In the ultrasound window, vertical artifacts represent 1/3 to 2/3 of the length of the pleural line.
- ( ) 3: In the ultrasound window, vertical artifacts represent more than 2/3 of the length of the pleural line.
- ( ) 4: Presence of consolidation.

137. How confident are you in your choice? *

Marcar apenas uma oval.

	1	2	3	4	5	
Not	( )	( )	( )	( )	( )	Very confident

138. How do you rate the quality of the video? *

Marcar apenas uma oval.

	1	2	3	4	5	
Low	( )	( )	( )	( )	( )	High quality

139. Note (if any)

Video 7

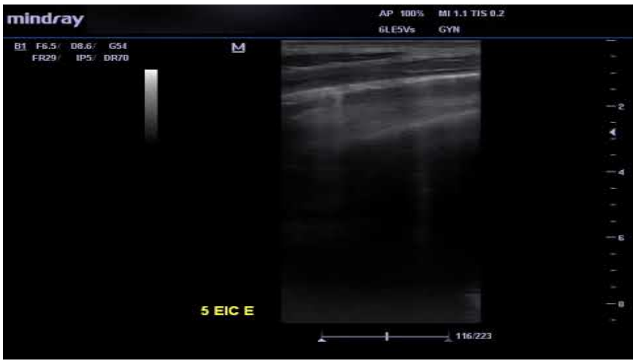

http://youtube.com/watch?v=T8rAt2WDGQ4

140. What score would you give this video? *

Marcar apenas uma oval.

- ( ) 0: Aerated lung with the presence of A-lines, with no pleural alteration.
- ( ) 1: In the ultrasound window, vertical artifacts represent less than 1/3 of the length of the pleural line.
- ( ) 2: In the ultrasound window, vertical artifacts represent 1/3 to 2/3 of the length of the pleural line.
- ( ) 3: In the ultrasound window, vertical artifacts represent more than 2/3 of the length of the pleural line.
- ( ) 4: Presence of consolidation.

141. How confident are you in your choice? *

Marcar apenas uma oval.

	1	2	3	4	5	
Not	( )	( )	( )	( )	( )	Very confident

142. How do you rate the quality of the video? *

Marcar apenas uma oval.

	1	2	3	4	5	
Low	◯	◯	◯	◯	◯	High quality

143. Note (if any)

Video 9

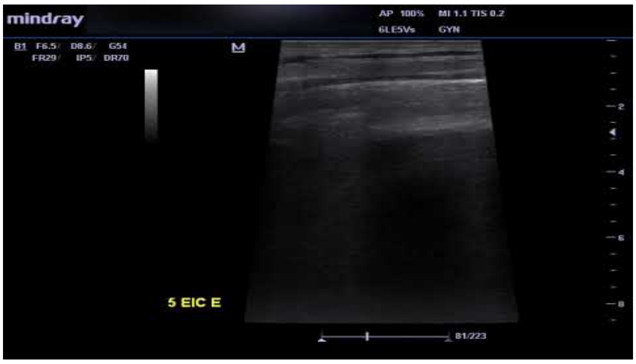

http://youtube.com/watch?v=LGNH5_eE1Dg

144. What score would you give this video? *

Marcar apenas uma oval.

- 0: Aerated lung with the presence of A-lines, with no pleural alteration.
- 1: In the ultrasound window, vertical artifacts represent less than 1/3 of the length of the pleural line.
- 2: In the ultrasound window, vertical artifacts represent 1/3 to 2/3 of the length of the pleural line.
- 3: In the ultrasound window, vertical artifacts represent more than 2/3 of the length of the pleural line.
- 4: Presence of consolidation.

145. How confident are you in your choice? *

Marcar apenas uma oval.

	1	2	3	4	5	
Not	○	○	○	○	○	Very confident

146. How do you rate the quality of the video? *

Marcar apenas uma oval.

	1	2	3	4	5	
Low	○	○	○	○	○	High quality

147. Note (if any)

Video 10

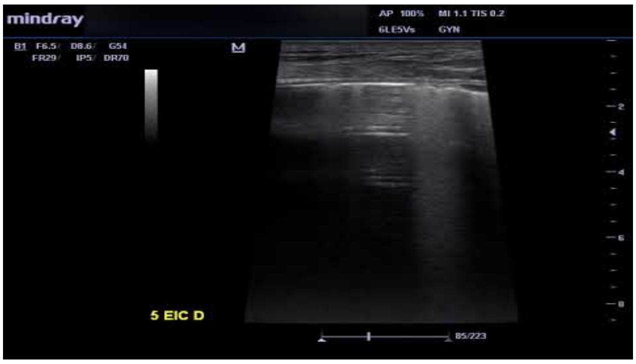

http://youtube.com/watch?v=6RetQGPooM4

148. What score would you give this video? *

Marcar apenas uma oval.

- 0: Aerated lung with the presence of A-lines, with no pleural alteration.
- 1: In the ultrasound window, vertical artifacts represent less than 1/3 of the length of the pleural line.
- 2: In the ultrasound window, vertical artifacts represent 1/3 to 2/3 of the length of the pleural line.
- 3: In the ultrasound window, vertical artifacts represent more than 2/3 of the length of the pleural line.
- 4: Presence of consolidation.

149. How confident are you in your choice? *

Marcar apenas uma oval.

	1	2	3	4	5	
Not						Very confident

150. How do you rate the quality of the video? *

Marcar apenas uma oval.

	1	2	3	4	5	
Low	⬭	⬭	⬭	⬭	⬭	High quality

151. Note (if any)

____
____
____
____
____

Video 11

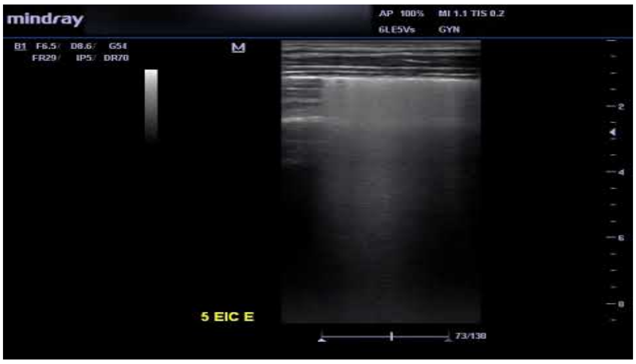

http://youtube.com/watch?v=Lt1TeVbPogM

152. What score would you give this video? *

Marcar apenas uma oval.

- 0: Aerated lung with the presence of A-lines, with no pleural alteration.
- 1: In the ultrasound window, vertical artifacts represent less than 1/3 of the length of the pleural line.
- 2: In the ultrasound window, vertical artifacts represent 1/3 to 2/3 of the length of the pleural line.
- 3: In the ultrasound window, vertical artifacts represent more than 2/3 of the length of the pleural line.
- 4: Presence of consolidation.

153. How confident are you in your choice? *

Marcar apenas uma oval.

	1	2	3	4	5	
Not	◯	◯	◯	◯	◯	Very confident

154. How do you rate the quality of the video? *

Marcar apenas uma oval.

	1	2	3	4	5	
Low	◯	◯	◯	◯	◯	High quality

155. Note (if any)

Video 12

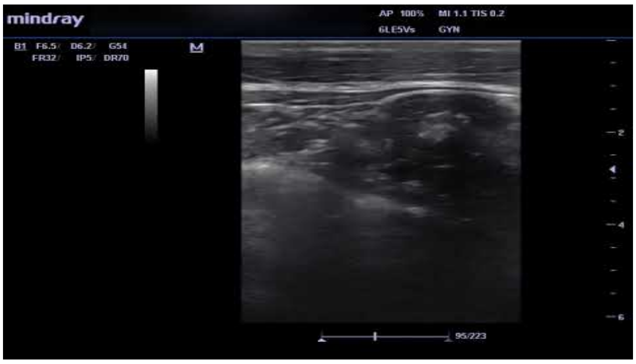

http://youtube.com/watch?v=D1j5qCsLJ0Q

156. What score would you give this video? *

Marcar apenas uma oval.

- 0: Aerated lung with the presence of A-lines, with no pleural alteration.
- 1: In the ultrasound window, vertical artifacts represent less than 1/3 of the length of the pleural line.
- 2: In the ultrasound window, vertical artifacts represent 1/3 to 2/3 of the length of the pleural line.
- 3: In the ultrasound window, vertical artifacts represent more than 2/3 of the length of the pleural line.
- 4: Presence of consolidation.

157. How confident are you in your choice? *

Marcar apenas uma oval.

	1	2	3	4	5	
Not	◯	◯	◯	◯	◯	Very confident

158. How do you rate the quality of the video? *

Marcar apenas uma oval.

	1	2	3	4	5	
Low	◯	◯	◯	◯	◯	High quality

159. Note (if any)

____

____

____

____

____

Video 13

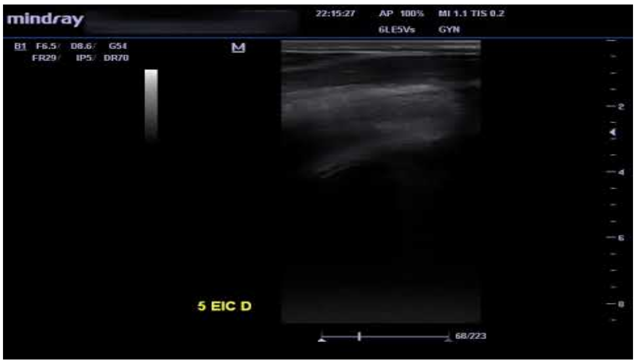

http://youtube.com/watch?v=jXXepFGPcUs

160. What score would you give this video? *

Marcar apenas uma oval.

- 0: Aerated lung with the presence of A-lines, with no pleural alteration.
- 1: In the ultrasound window, vertical artifacts represent less than 1/3 of the length of the pleural line.
- 2: In the ultrasound window, vertical artifacts represent 1/3 to 2/3 of the length of the pleural line.
- 3: In the ultrasound window, vertical artifacts represent more than 2/3 of the length of the pleural line.
- 4: Presence of consolidation.

161. How confident are you in your choice? *

Marcar apenas uma oval.

	1	2	3	4	5	
Not						Very confident

162. How do you rate the quality of the video? *

Marcar apenas uma oval.

	1	2	3	4	5	
Low						High quality

163. Note (if any)

Video 14

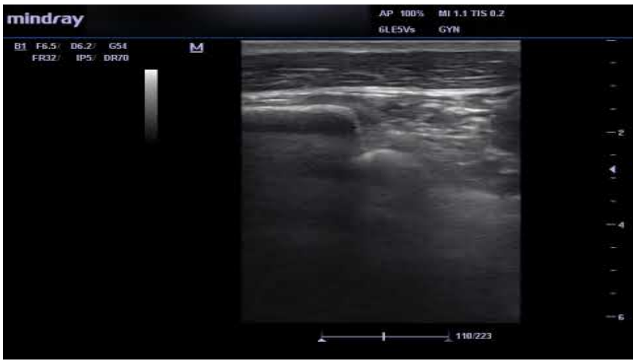

http://youtube.com/watch?v=NA_L6JpB7BQ

164. What score would you give this video? *

Marcar apenas uma oval.

- ⬭ 0: Aerated lung with the presence of A-lines, with no pleural alteration.
- ⬭ 1: In the ultrasound window, vertical artifacts represent less than 1/3 of the length of the pleural line.
- ⬭ 2: In the ultrasound window, vertical artifacts represent 1/3 to 2/3 of the length of the pleural line.
- ⬭ 3: In the ultrasound window, vertical artifacts represent more than 2/3 of the length of the pleural line.
- ⬭ 4: Presence of consolidation.

165. How confident are you in your choice? *

Marcar apenas uma oval.

	1	2	3	4	5	
Not	⬭	⬭	⬭	⬭	⬭	Very confident

166. How do you rate the quality of the video? *

Marcar apenas uma oval.

	1	2	3	4	5	
Low	◯	◯	◯	◯	◯	High quality

167. Note (if any)

____

____

____

____

____

Video 15

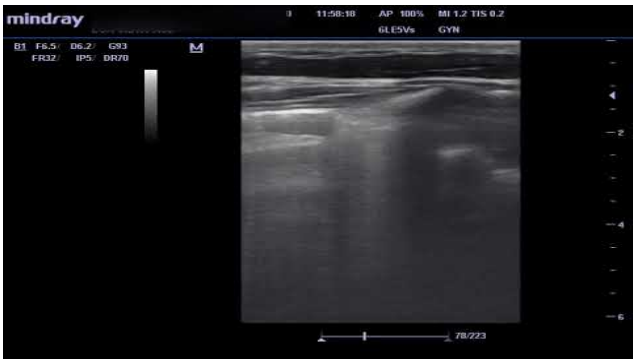

http://youtube.com/watch?v=a7bXjok7V2U

168. What score would you give this video? *

Marcar apenas uma oval.

- 0: Aerated lung with the presence of A-lines, with no pleural alteration.
- 1: In the ultrasound window, vertical artifacts represent less than 1/3 of the length of the pleural line.
- 2: In the ultrasound window, vertical artifacts represent 1/3 to 2/3 of the length of the pleural line.
- 3: In the ultrasound window, vertical artifacts represent more than 2/3 of the length of the pleural line.
- 4: Presence of consolidation.

169. How confident are you in your choice? *

Marcar apenas uma oval.

	1	2	3	4	5	
Not						Very confident

170. How do you rate the quality of the video? *

Marcar apenas uma oval.

	1	2	3	4	5	
Low						High quality

171. Note (if any)

Video 16

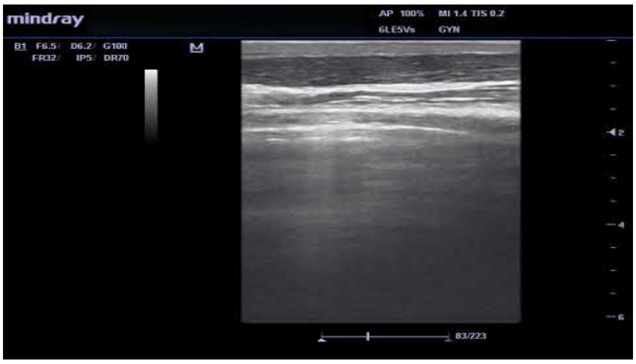 http://youtube.com/watch?v=90DtynHjwQM

172. What score would you give this video? *

Marcar apenas uma oval.

- 0: Aerated lung with the presence of A-lines, with no pleural alteration.
- 1: In the ultrasound window, vertical artifacts represent less than 1/3 of the length of the pleural line.
- 2: In the ultrasound window, vertical artifacts represent 1/3 to 2/3 of the length of the pleural line.
- 3: In the ultrasound window, vertical artifacts represent more than 2/3 of the length of the pleural line.
- 4: Presence of consolidation.

173. How confident are you in your choice? *

Marcar apenas uma oval.

	1	2	3	4	5	
Not						Very confident

174. How do you rate the quality of the video? *

Marcar apenas uma oval.

	1	2	3	4	5	
Low	◯	◯	◯	◯	◯	High quality

175. Note (if any)

____

____

____

____

____

Video 17

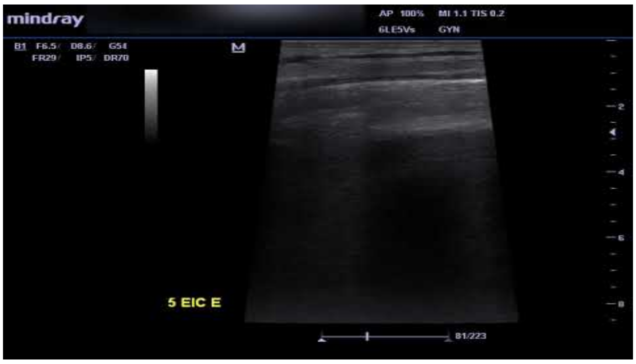

http://youtube.com/watch?v=nNLnlt6QWUU

176. What score would you give this video? *

Marcar apenas uma oval.

- 0: Aerated lung with the presence of A-lines, with no pleural alteration.
- 1: In the ultrasound window, vertical artifacts represent less than 1/3 of the length of the pleural line.
- 2: In the ultrasound window, vertical artifacts represent 1/3 to 2/3 of the length of the pleural line.
- 3: In the ultrasound window, vertical artifacts represent more than 2/3 of the length of the pleural line.
- 4: Presence of consolidation.

177. How confident are you in your choice? *

Marcar apenas uma oval.

	1	2	3	4	5	
Not						Very confident

178. How do you rate the quality of the video? *

Marcar apenas uma oval.

	1	2	3	4	5	
Low						High quality

179. Note (if any)

Video 18

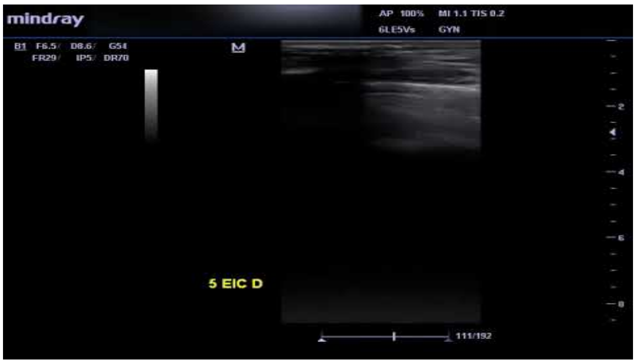

http://youtube.com/watch?v=kNgqBgtfbEA

180. What score would you give this video? *

Marcar apenas uma oval.

- 0: Aerated lung with the presence of A-lines, with no pleural alteration.
- 1: In the ultrasound window, vertical artifacts represent less than 1/3 of the length of the pleural line.
- 2: In the ultrasound window, vertical artifacts represent 1/3 to 2/3 of the length of the pleural line.
- 3: In the ultrasound window, vertical artifacts represent more than 2/3 of the length of the pleural line.
- 4: Presence of consolidation.

181. How confident are you in your choice? *

Marcar apenas uma oval.

	1	2	3	4	5	
Not	◯	◯	◯	◯	◯	Very confident

182. How do you rate the quality of the video? *

Marcar apenas uma oval.

	1	2	3	4	5	
Low	◯	◯	◯	◯	◯	High quality

183. Note (if any)

____

____

____

____

____

Video 19

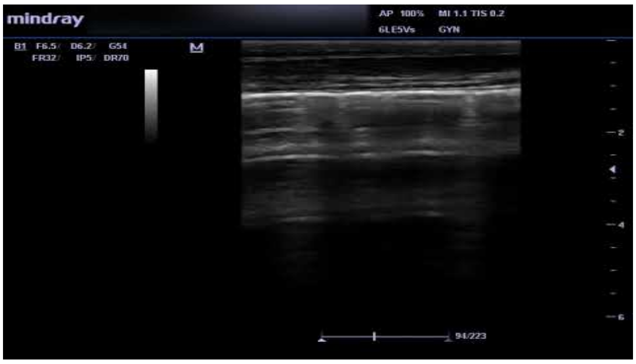

http://youtube.com/watch?v=eVAGezC7bzM

184. What score would you give this video? *

Marcar apenas uma oval.

- 0: Aerated lung with the presence of A-lines, with no pleural alteration.
- 1: In the ultrasound window, vertical artifacts represent less than 1/3 of the length of the pleural line.
- 2: In the ultrasound window, vertical artifacts represent 1/3 to 2/3 of the length of the pleural line.
- 3: In the ultrasound window, vertical artifacts represent more than 2/3 of the length of the pleural line.
- 4: Presence of consolidation.

185. How confident are you in your choice? *

Marcar apenas uma oval.

	1	2	3	4	5	
Not						Very confident

186. How do you rate the quality of the video? *

Marcar apenas uma oval.

	1	2	3	4	5	
Low						High quality

187. Note (if any)

Video 20

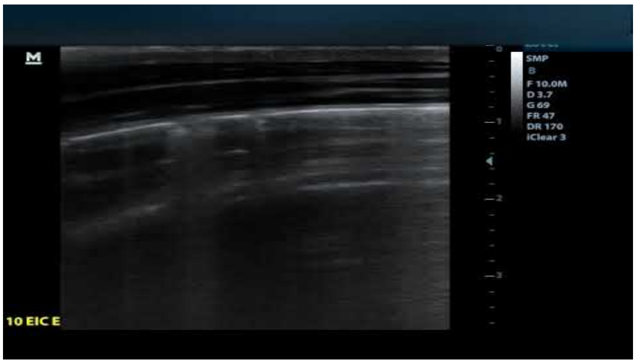

http://youtube.com/watch?v=zjMse9a51Hk

188. What score would you give this video? *

Marcar apenas uma oval.

- ◯ 0: Aerated lung with the presence of A-lines, with no pleural alteration.
- ◯ 1: In the ultrasound window, vertical artifacts represent less than 1/3 of the length of the pleural line.
- ◯ 2: In the ultrasound window, vertical artifacts represent 1/3 to 2/3 of the length of the pleural line.
- ◯ 3: In the ultrasound window, vertical artifacts represent more than 2/3 of the length of the pleural line.
- ◯ 4: Presence of consolidation.

189. How confident are you in your choice? *

Marcar apenas uma oval.

	1	2	3	4	5	
Not	◯	◯	◯	◯	◯	Very confident

190. How do you rate the quality of the video? *

Marcar apenas uma oval.

	1	2	3	4	5	
Low	◯	◯	◯	◯	◯	High quality

191. Note (if any)

Video 21

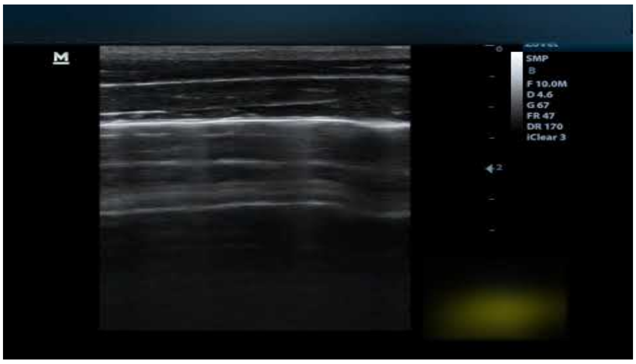

http://youtube.com/watch?v=b-G_LqTGC7I

192. What score would you give this video? *

Marcar apenas uma oval.

- 0: Aerated lung with the presence of A-lines, with no pleural alteration.
- 1: In the ultrasound window, vertical artifacts represent less than 1/3 of the length of the pleural line.
- 2: In the ultrasound window, vertical artifacts represent 1/3 to 2/3 of the length of the pleural line.
- 3: In the ultrasound window, vertical artifacts represent more than 2/3 of the length of the pleural line.
- 4: Presence of consolidation.

193. How confident are you in your choice? *

Marcar apenas uma oval.

	1	2	3	4	5	
Not						Very confident

194. How do you rate the quality of the video? *

Marcar apenas uma oval.

	1	2	3	4	5	
Low						High quality

195. Note (if any)

Video 22

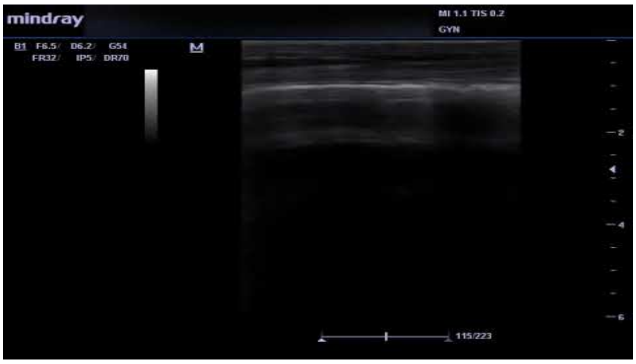

http://youtube.com/watch?v=01ja0hULH70

196. What score would you give this video? *

Marcar apenas uma oval.

- ( ) 0: Aerated lung with the presence of A-lines, with no pleural alteration.
- ( ) 1: In the ultrasound window, vertical artifacts represent less than 1/3 of the length of the pleural line.
- ( ) 2: In the ultrasound window, vertical artifacts represent 1/3 to 2/3 of the length of the pleural line.
- ( ) 3: In the ultrasound window, vertical artifacts represent more than 2/3 of the length of the pleural line.
- ( ) 4: Presence of consolidation.

197. How confident are you in your choice? *

Marcar apenas uma oval.

	1	2	3	4	5	
Not	( )	( )	( )	( )	( )	Very confident

198. How do you rate the quality of the video? *

Marcar apenas uma oval.

	1	2	3	4	5	
Low	◯	◯	◯	◯	◯	High quality

199. Note (if any)

Video 23

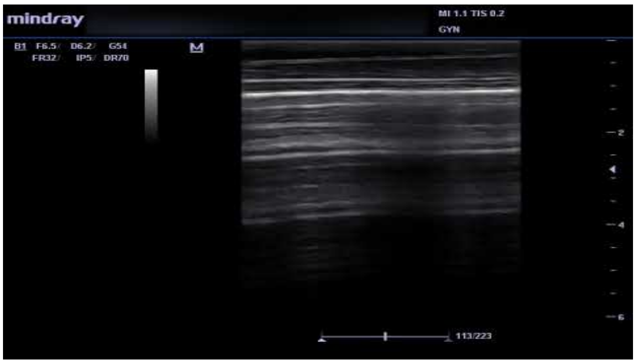

http://youtube.com/watch?v=ySCy_n5_nLw

200. What score would you give this video? *

Marcar apenas uma oval.

- 0: Aerated lung with the presence of A-lines, with no pleural alteration.
- 1: In the ultrasound window, vertical artifacts represent less than 1/3 of the length of the pleural line.
- 2: In the ultrasound window, vertical artifacts represent 1/3 to 2/3 of the length of the pleural line.
- 3: In the ultrasound window, vertical artifacts represent more than 2/3 of the length of the pleural line.
- 4: Presence of consolidation.

201. How confident are you in your choice? *

Marcar apenas uma oval.

	1	2	3	4	5	
Not						Very confident

202. How do you rate the quality of the video? *

Marcar apenas uma oval.

	1	2	3	4	5	
Low						High quality

203. Note (if any)

Video 24

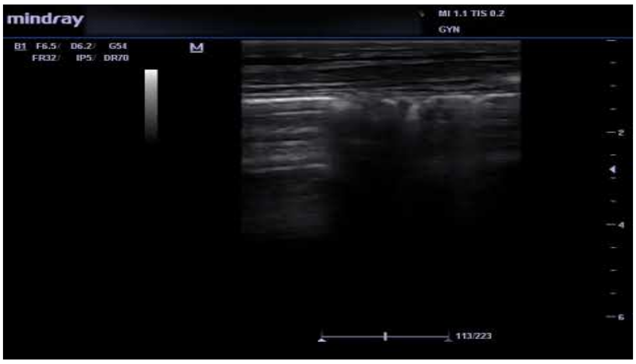

http://youtube.com/watch?v=35ZMB_ZxnyU

204. What score would you give this video? *

Marcar apenas uma oval.

- ◯ 0: Aerated lung with the presence of A-lines, with no pleural alteration.
- ◯ 1: In the ultrasound window, vertical artifacts represent less than 1/3 of the length of the pleural line.
- ◯ 2: In the ultrasound window, vertical artifacts represent 1/3 to 2/3 of the length of the pleural line.
- ◯ 3: In the ultrasound window, vertical artifacts represent more than 2/3 of the length of the pleural line.
- ◯ 4: Presence of consolidation.

205. How confident are you in your choice? *

Marcar apenas uma oval.

	1	2	3	4	5	
Not	◯	◯	◯	◯	◯	Very confident

206. How do you rate the quality of the video? *

Marcar apenas uma oval.

	1	2	3	4	5	
Low	◯	◯	◯	◯	◯	High quality

207. Note (if any)

____

____

____

____

____

Video 25

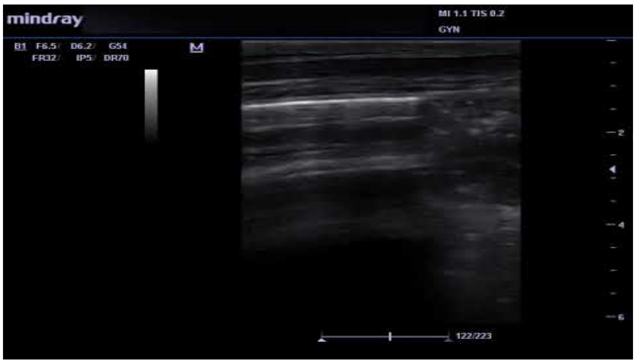

http://youtube.com/watch?v=xvm2Lmt_6u4

208. What score would you give this video? *

Marcar apenas uma oval.

- ( ) 0: Aerated lung with the presence of A-lines, with no pleural alteration.
- ( ) 1: In the ultrasound window, vertical artifacts represent less than 1/3 of the length of the pleural line.
- ( ) 2: In the ultrasound window, vertical artifacts represent 1/3 to 2/3 of the length of the pleural line.
- ( ) 3: In the ultrasound window, vertical artifacts represent more than 2/3 of the length of the pleural line.
- ( ) 4: Presence of consolidation.

209. How confident are you in your choice? *

Marcar apenas uma oval.

	1	2	3	4	5	
Not	( )	( )	( )	( )	( )	Very confident

210. How do you rate the quality of the video? *

Marcar apenas uma oval.

	1	2	3	4	5	
Low	( )	( )	( )	( )	( )	High quality

211. Note (if any)

____
____
____
____
____

Este conteúdo não foi criado nem aprovado pelo Google.

Google Formulários
