# Supplementary material for: Inter-rater agreement of a newborn calf lung ultrasound scoring system
Source: J Vet Intern Med. 2026 Apr 21;40(2):aalag067. doi: 10.1093/jvimsj/aalag067 (PMC13098365; doi:10.1093/jvimsj/aalag067)

Reliability assessment of lung ultrasound image scores in bovine neonatology

A. C. A Abreu[1], V. Gomes [1], S. Buczinski [2] .

[1]Department of Internal Medicine, Faculty of Veterinary Medicine and Animal Science, University of São Paulo, São Paulo, Brazil.
[2] Faculty of Veterinary Medicine, Université de Montréal, Saint-Hyacinthe, Canada.

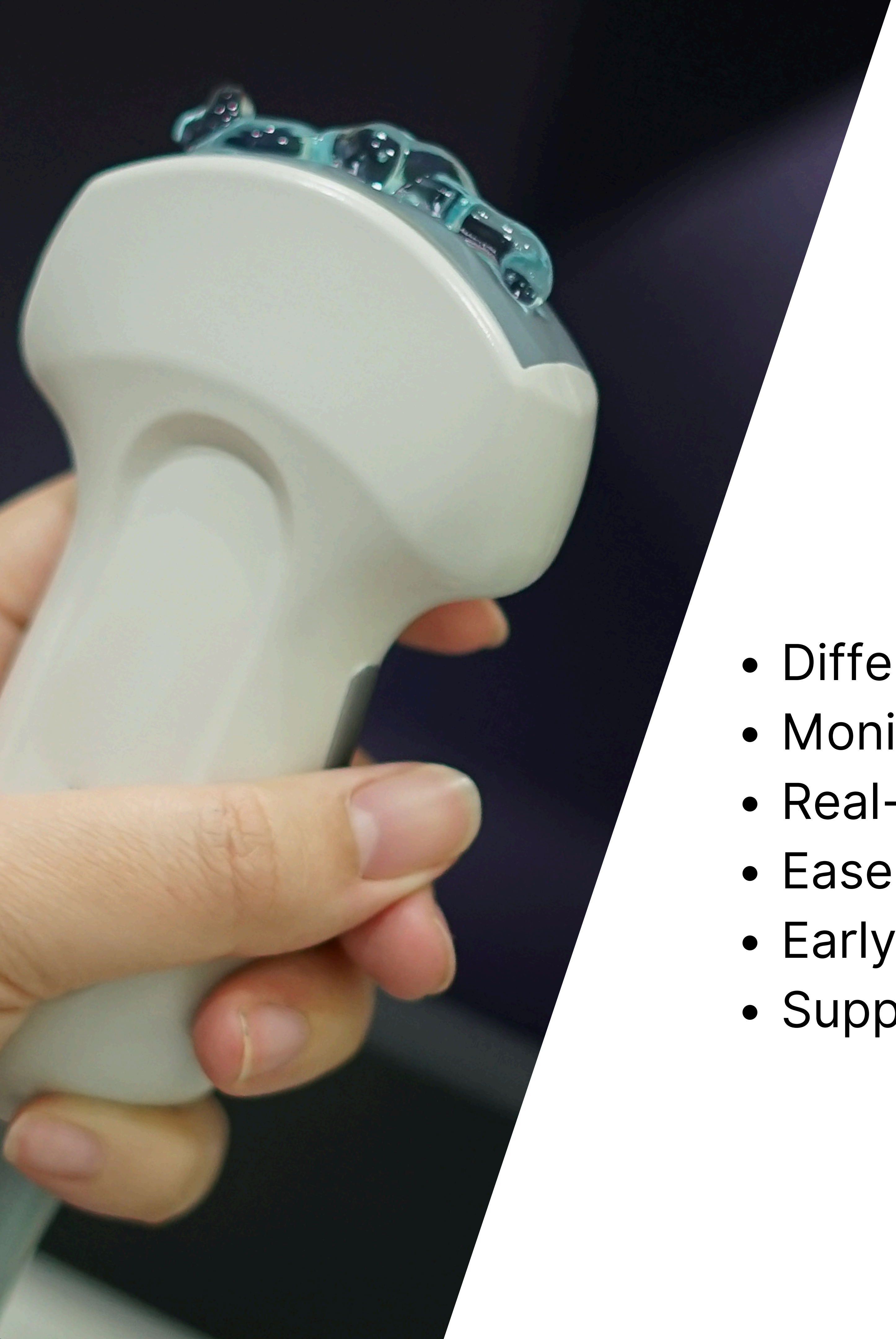

Importance of Lung Ultrasound

- Differentiation Between Physiological and Pathological Changes
- Monitoring of Pulmonary Clearance
- Real-Time Assessment
- Ease of Use in the Field
- Early Detection of Anomalies
- Support for Differential Diagnosis

FMVZ USP GECRIA Université de Montréal

Advantages:

- NON-INVASIVE METHOD
- NO RADIATION EXPOSURE
- EASY PORTABILITY
- DYNAMIC IMAGING
- COMPLEMENTARY TO CLINICAL EXAMINATION

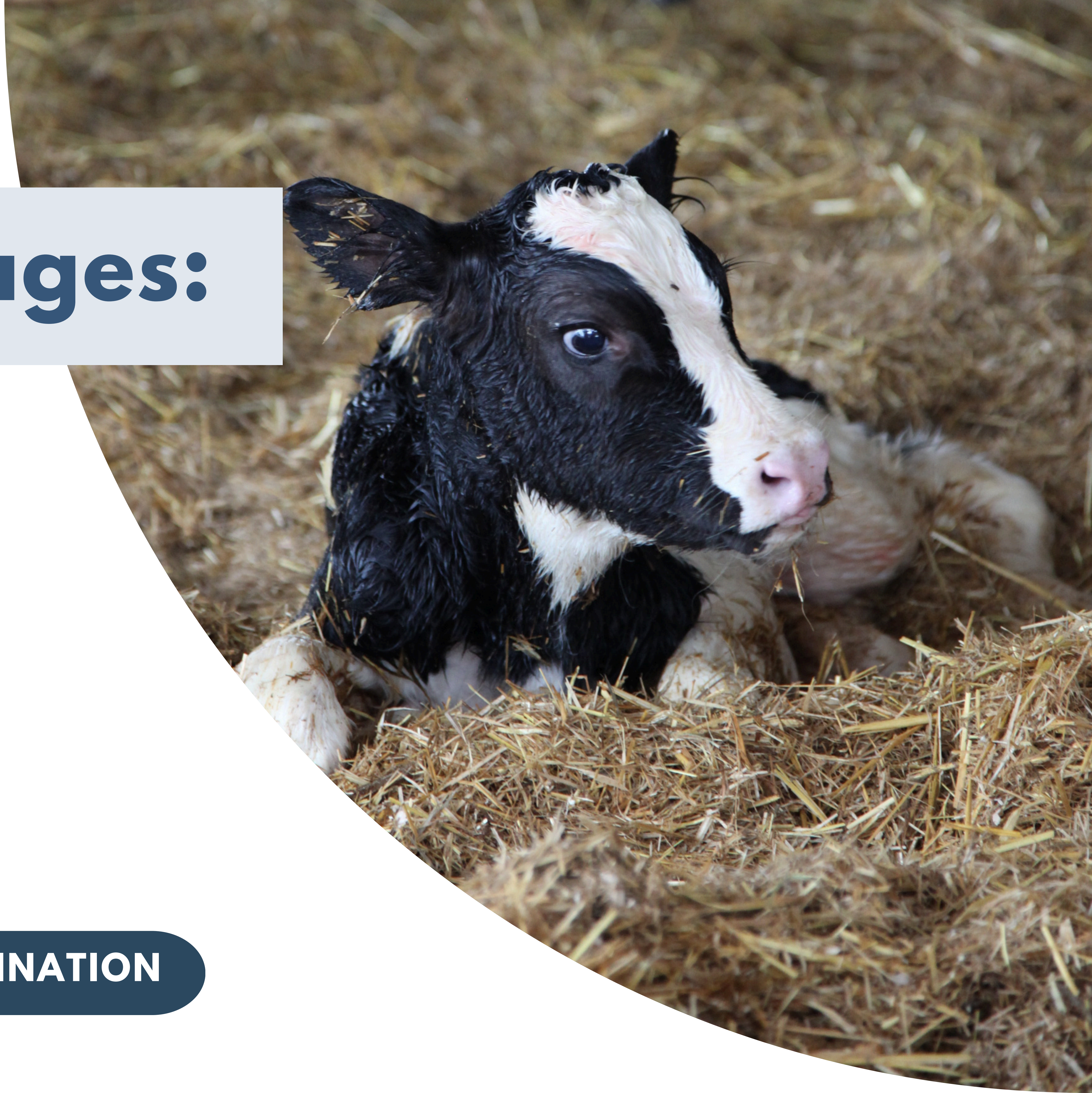

Why is it important to perform a neonatal lung ultrasound scoring in calves?

UNDERSTANDING THE PHYSIOLOGY OF PULMONARY CLEARANCE

ASSISTING IN EARLY DIAGNOSIS OF BRONCHOPNEUMONIA

TO UNDERSTAND WHEN TO USE POSSIBLE INTERVENTIONS AFTER CALVING.

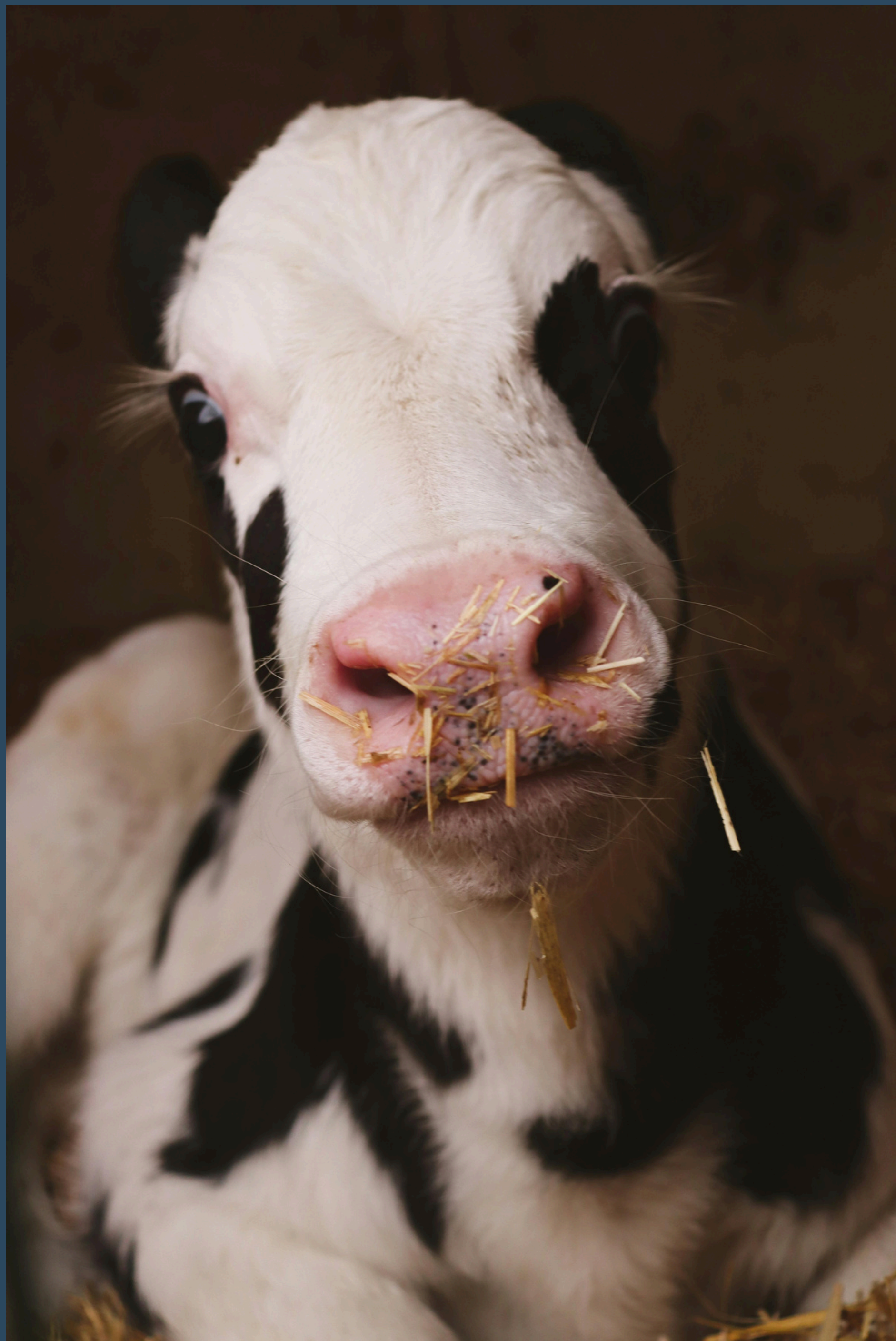

Project Objective:

Reliability of Lung Ultrasound Image Scoring in Bovine Neonates.

How Does It Work?

In the following slides, you will receive a brief explanation of the ultrasound principles.

We will demonstrate videos with the classification of **5 scores** to help you identify them.

In the form, you will evaluate and classify each image or video according to the scores.

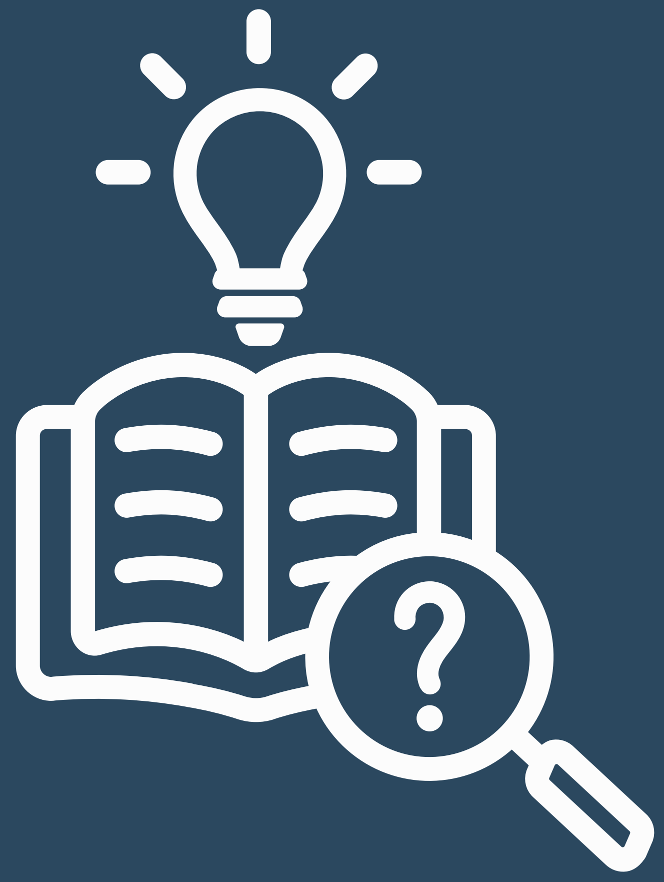

"A" Lines

A-lines are horizontal, hyperechoic lines parallel to the pleural line, formed by the reverberation of the ultrasound wave within the pleura, equidistant from each other.

This pattern indicates normal lung aeration, characterized by the presence of A-lines.

As the patient breathes, these structures move relative to the superficial tissues, which we call "pleural sliding."

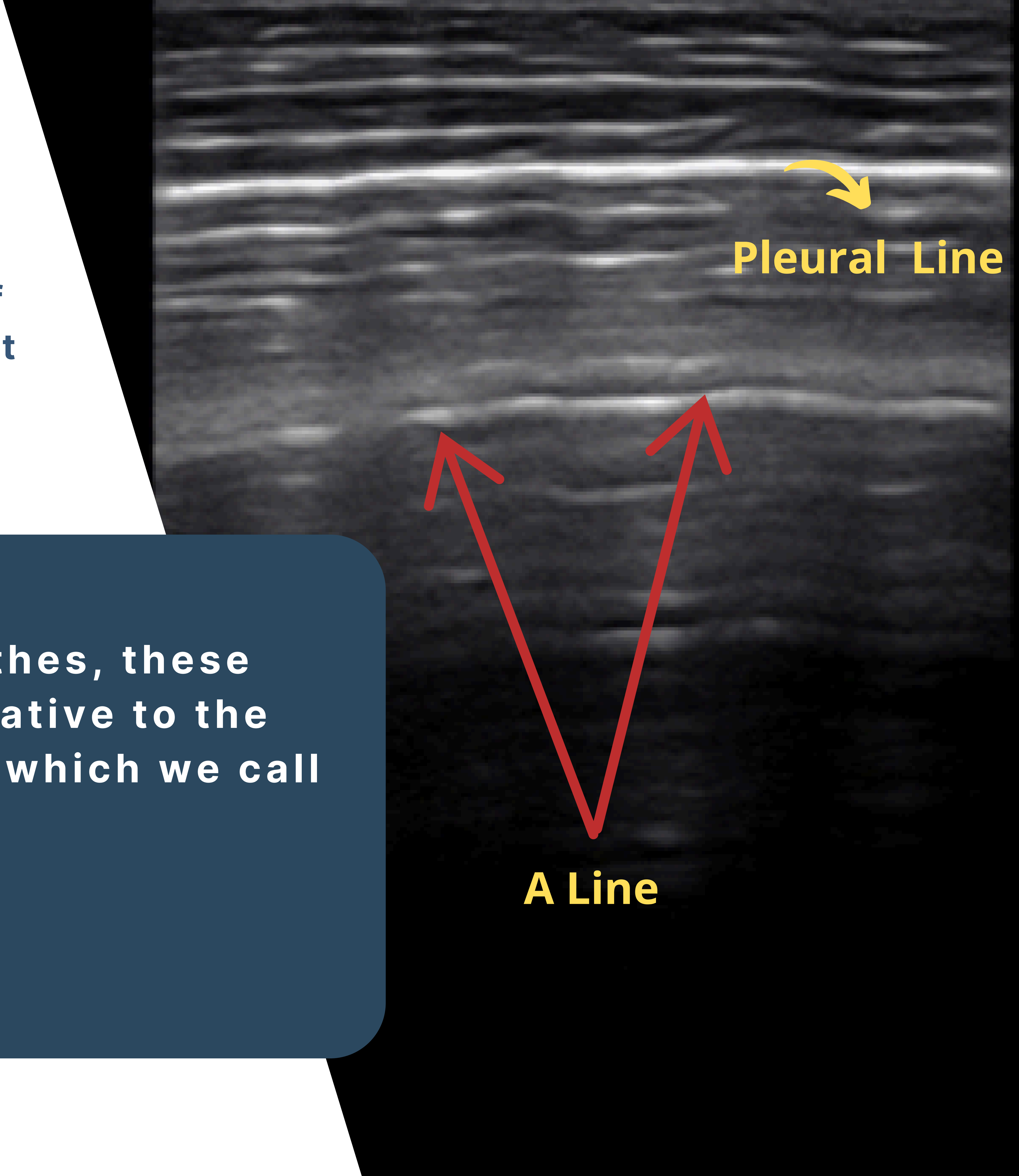

Vertical Artifacts

Called B-lines, these are characterized as vertical artifacts perpendicular to the pleural line, with a "comet-tail" appearance. They move along with the pleural line, erasing the A-lines.

.

They move in sync with respiratory movements.

B-lines appear when the ultrasound sound wave interacts with a fluid-gas interface, which occurs in conditions such as pulmonary contusion or edema. On the scan, they appear as hyperechoic (bright) bands.

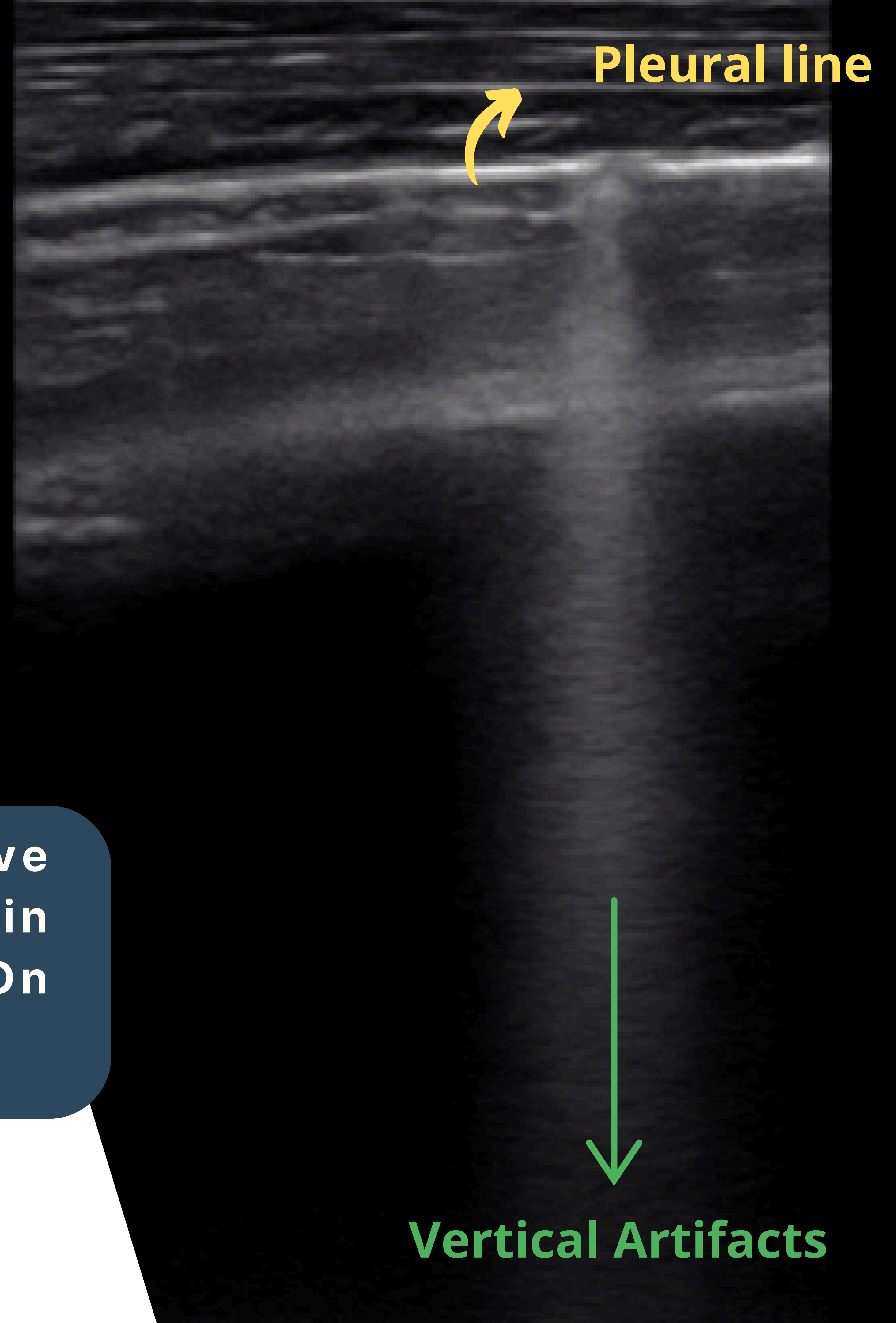

White Lung

White lung is a severe vertical artifact in lung ultrasound, appearing predominantly echogenic **(white and blurry)** instead of showing the typical hypoechoic **(black)** lines of an aerated lung.

The increased fluid is mainly in the **interstitial space**, distinguishing it from consolidation, where alveoli are filled with fluid or cells. Since alveoli still contain air, white lung is an artifact, not a direct depiction of lung structure.

Key ultrasound features include **multiple coalescing B-lines**, **absence of A-lines**, and limited visualization of deeper structures, unlike consolidation, which may reveal air bronchograms.

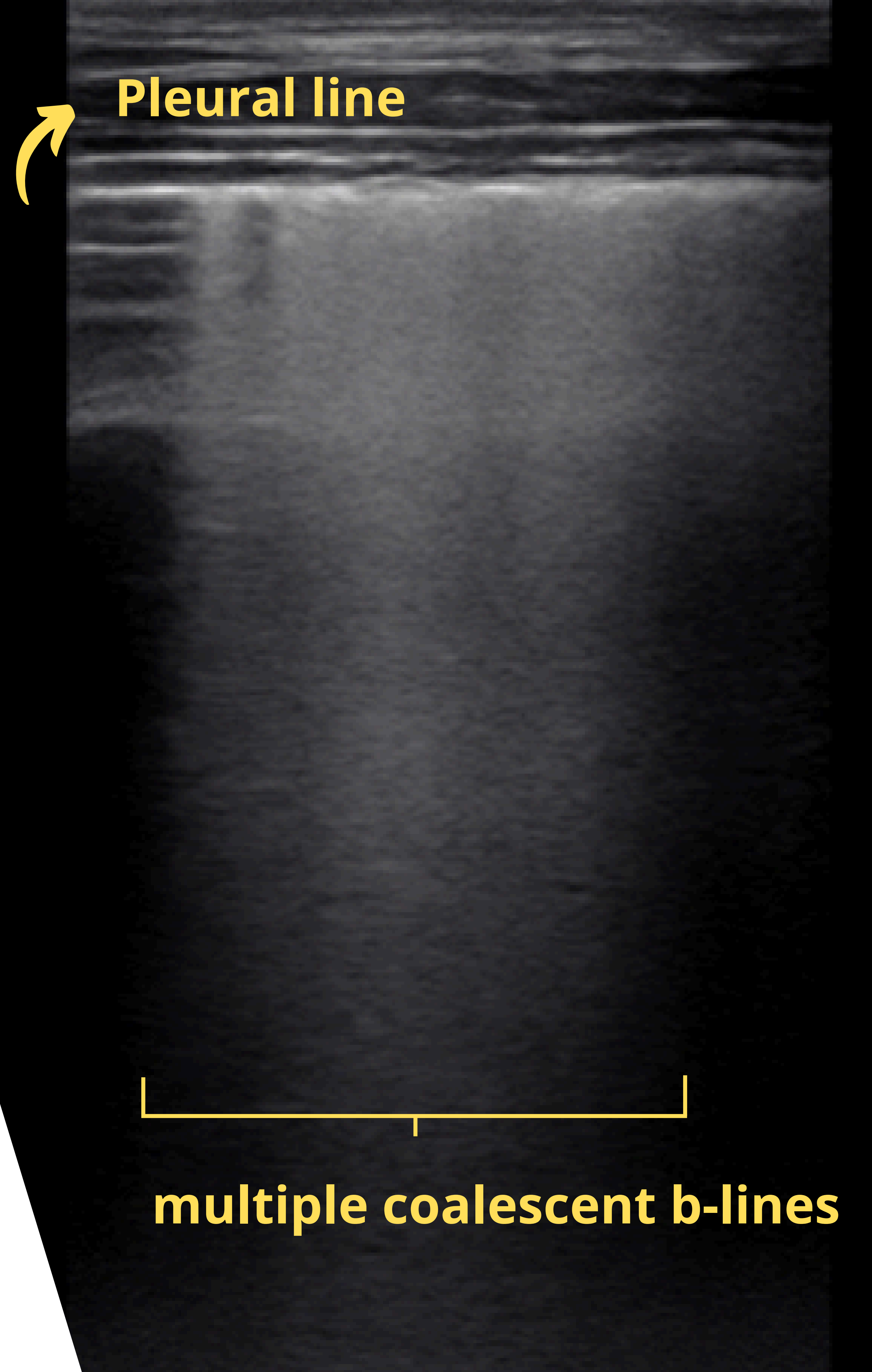

White Lung

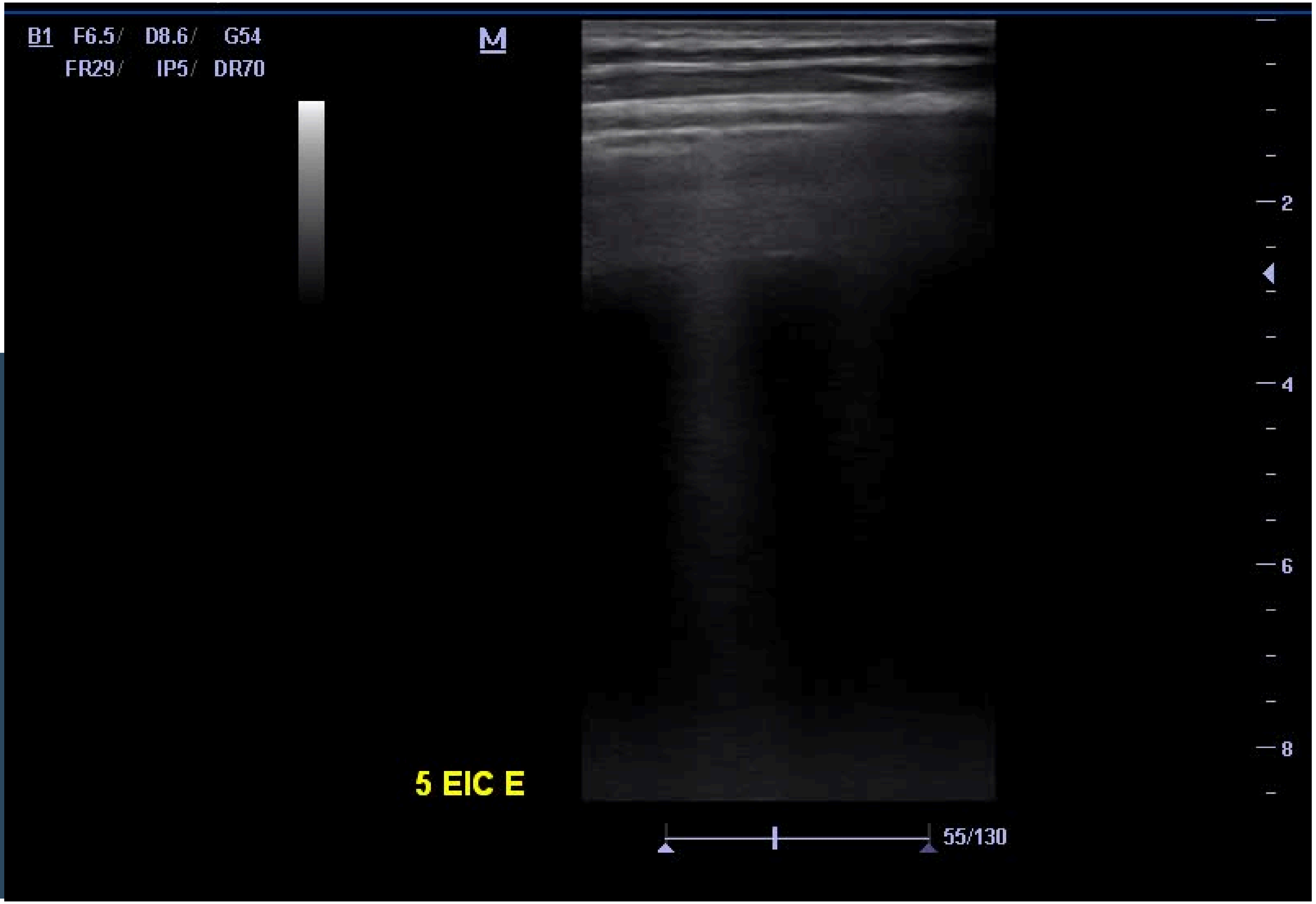

Consolidation

In this condition, the air-filled spaces in the pulmonary alveoli are replaced by fluid, blood, or other substances. On ultrasound, consolidations appear as small, can be circular or irregular lung consolidation (shred-sign), and hyperechoic structures.

The finding of atelectasis or pulmonary consolidation involves a loss of aeration, creating an area of visible parenchyma with a liver-like texture, having poorly defined and irregular borders.

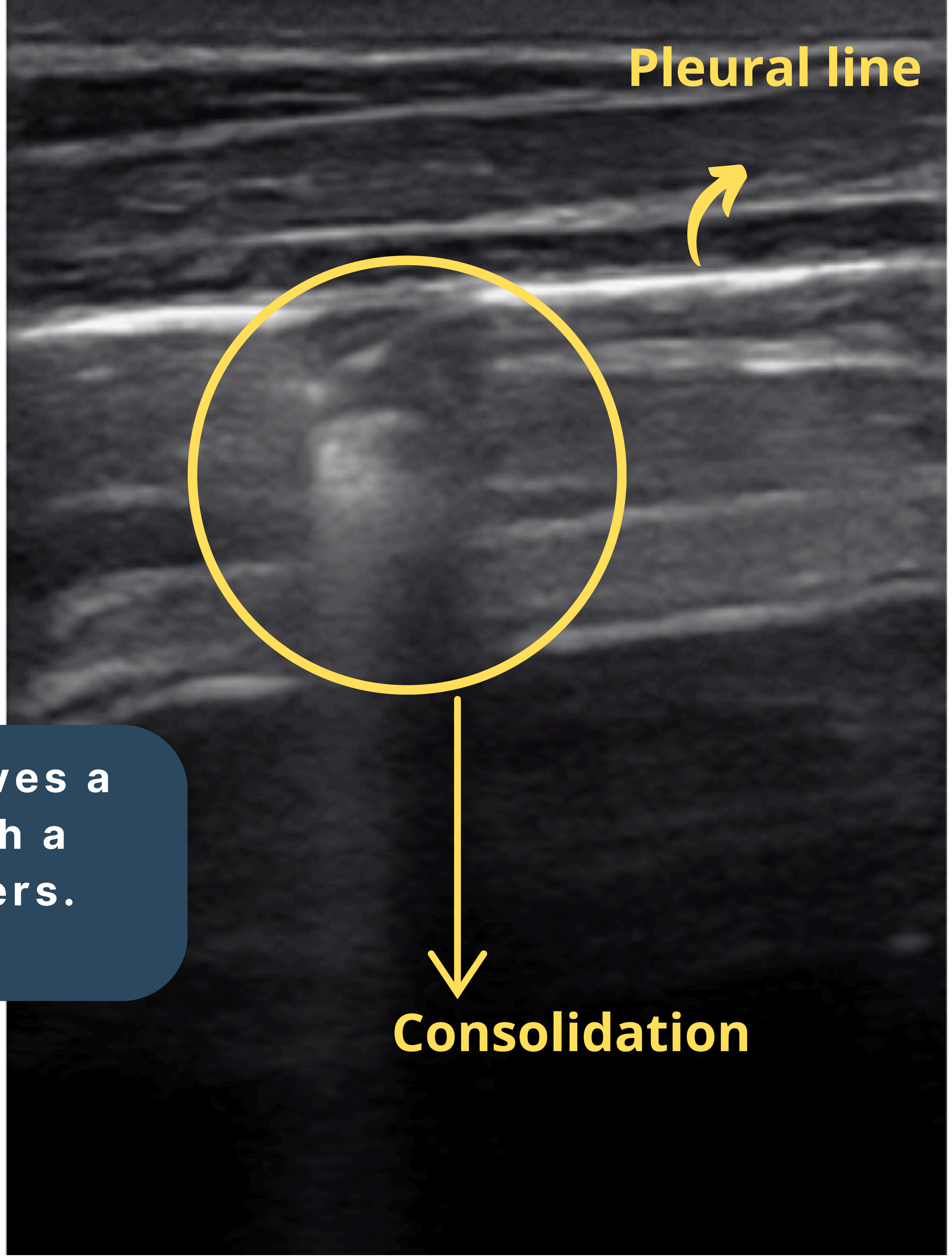

Lung Ultrasound Scoring

0 NORMAL VISUALIZATION OF A-LINE THROUGHOUT THE LOOP

1 THE VERTICAL ARTIFACTS REPRESENTS <1/3 OF THE PLEURAL LINE LENGTH

2 THE VERTICAL ARTIFACTS REPRESENTS 1/3-2/3 OF THE PLEURAL LINE LENGTH

3 THE VERTICAL ARTIFACTS REPRESENTS >2/3 OF THE PLEURAL LINE LENGTH

4 PRESENCE OF CONSOLIDATION

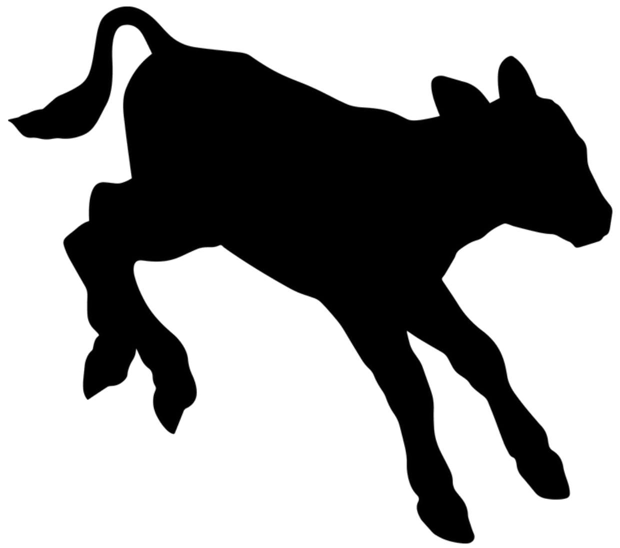

Is important to note that some images may present different ultrasound findings simultaneously. You should select the feature that occupies the majority of the image or video.

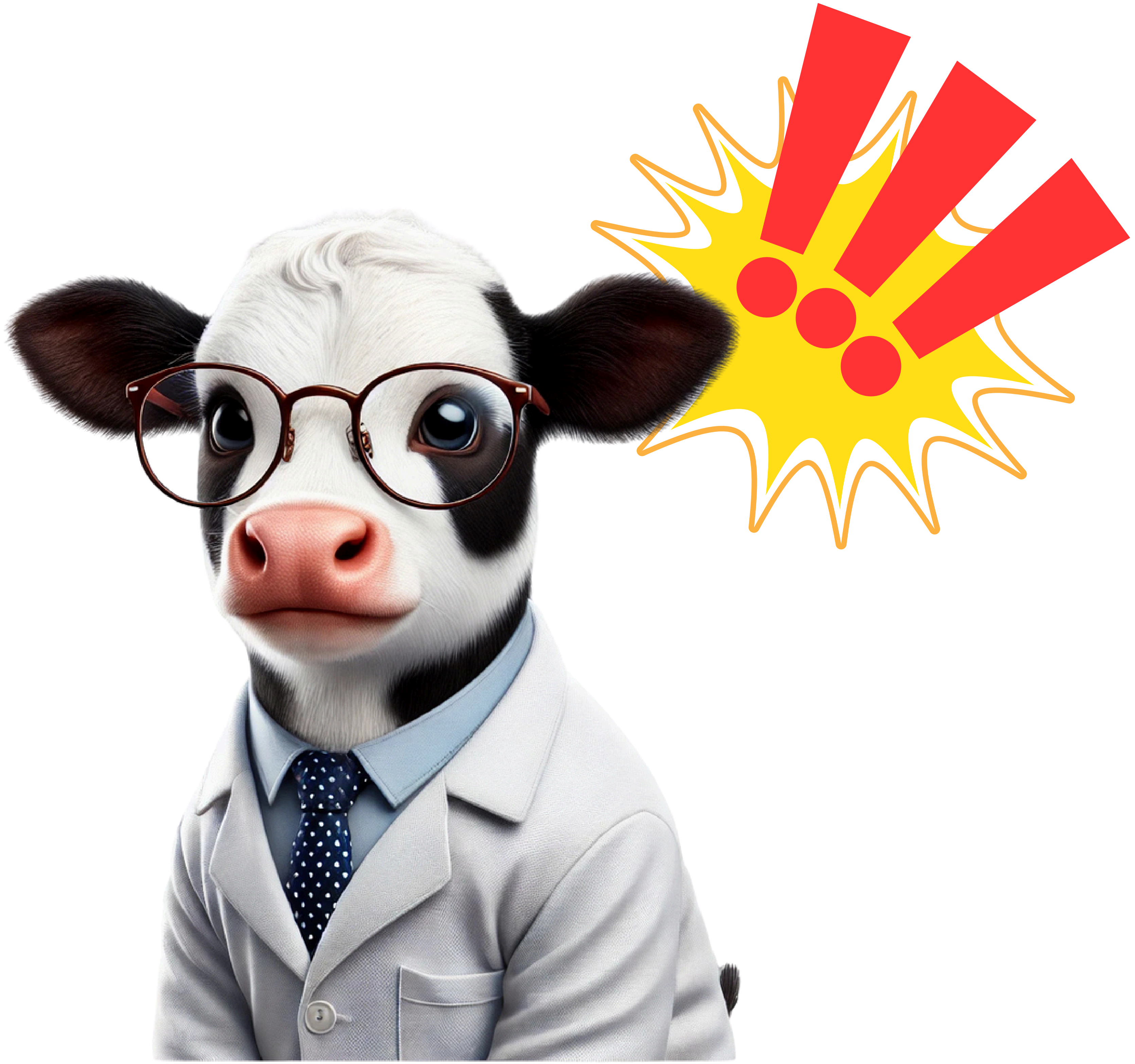

0: Aerated lung with the presence of A-lines, with no pleural alteration.

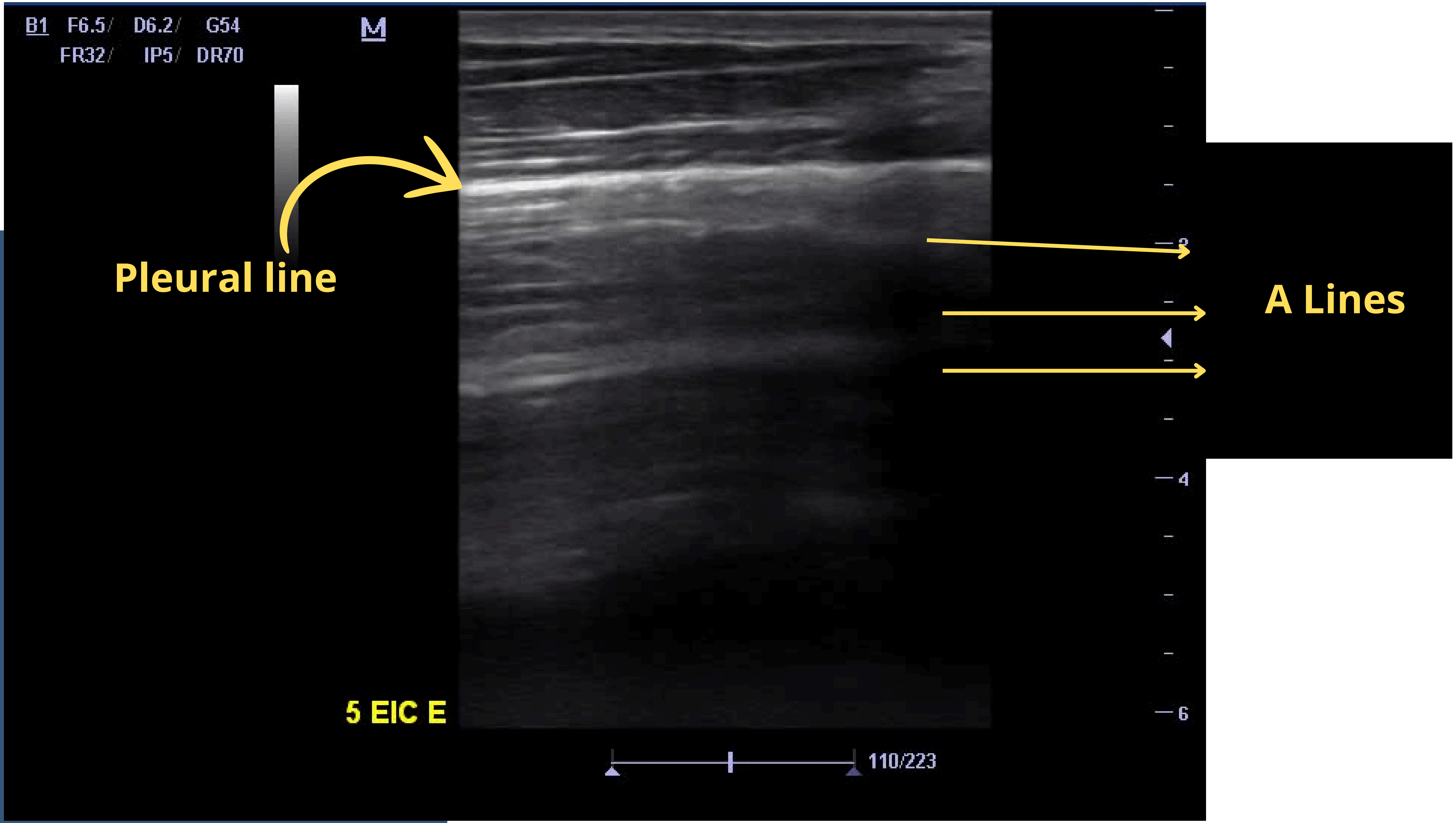

1-The vertical artifacts represents <1/3 of the pleural line length

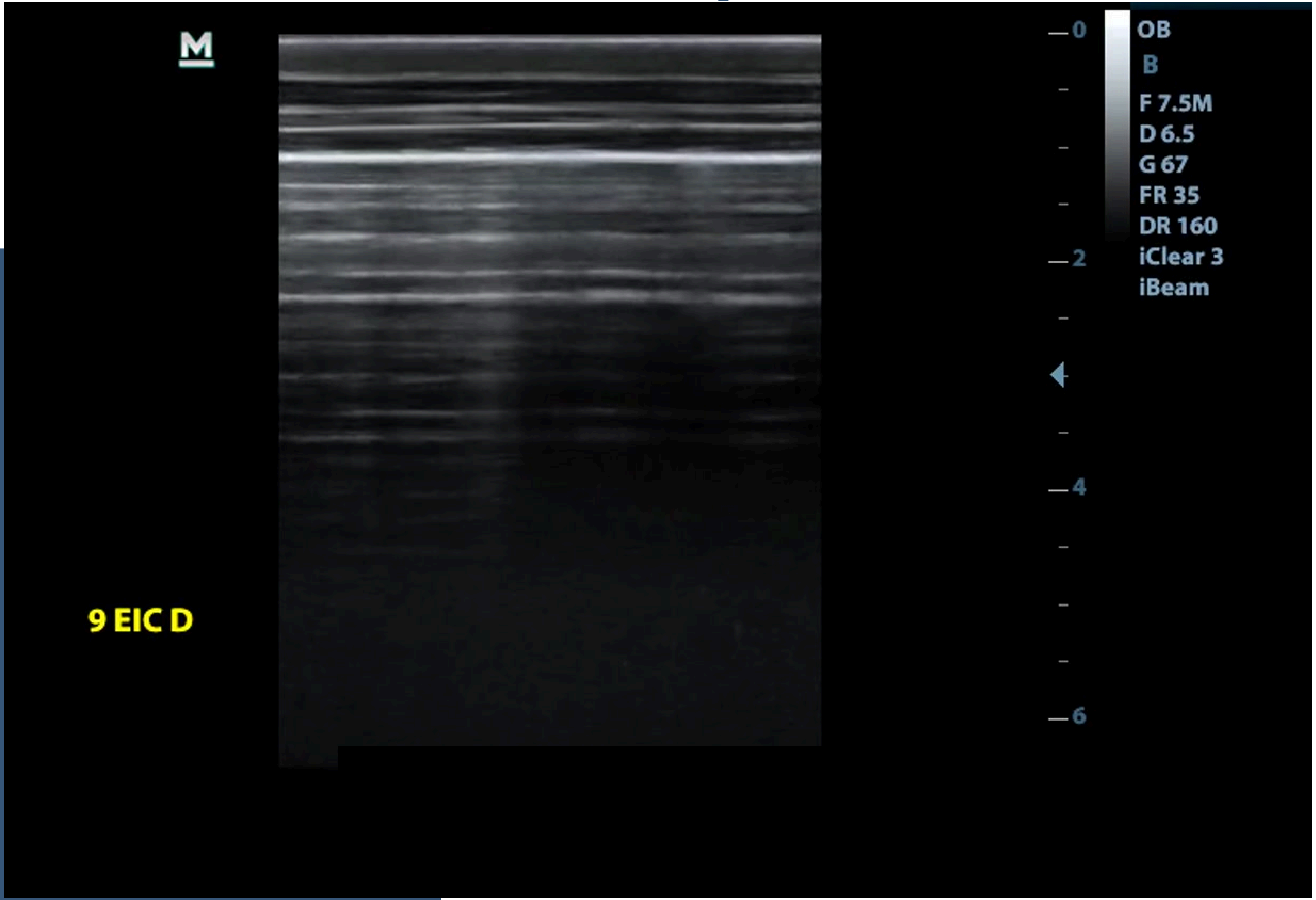

1-The vertical artifacts represents <1/3 of the pleural line length

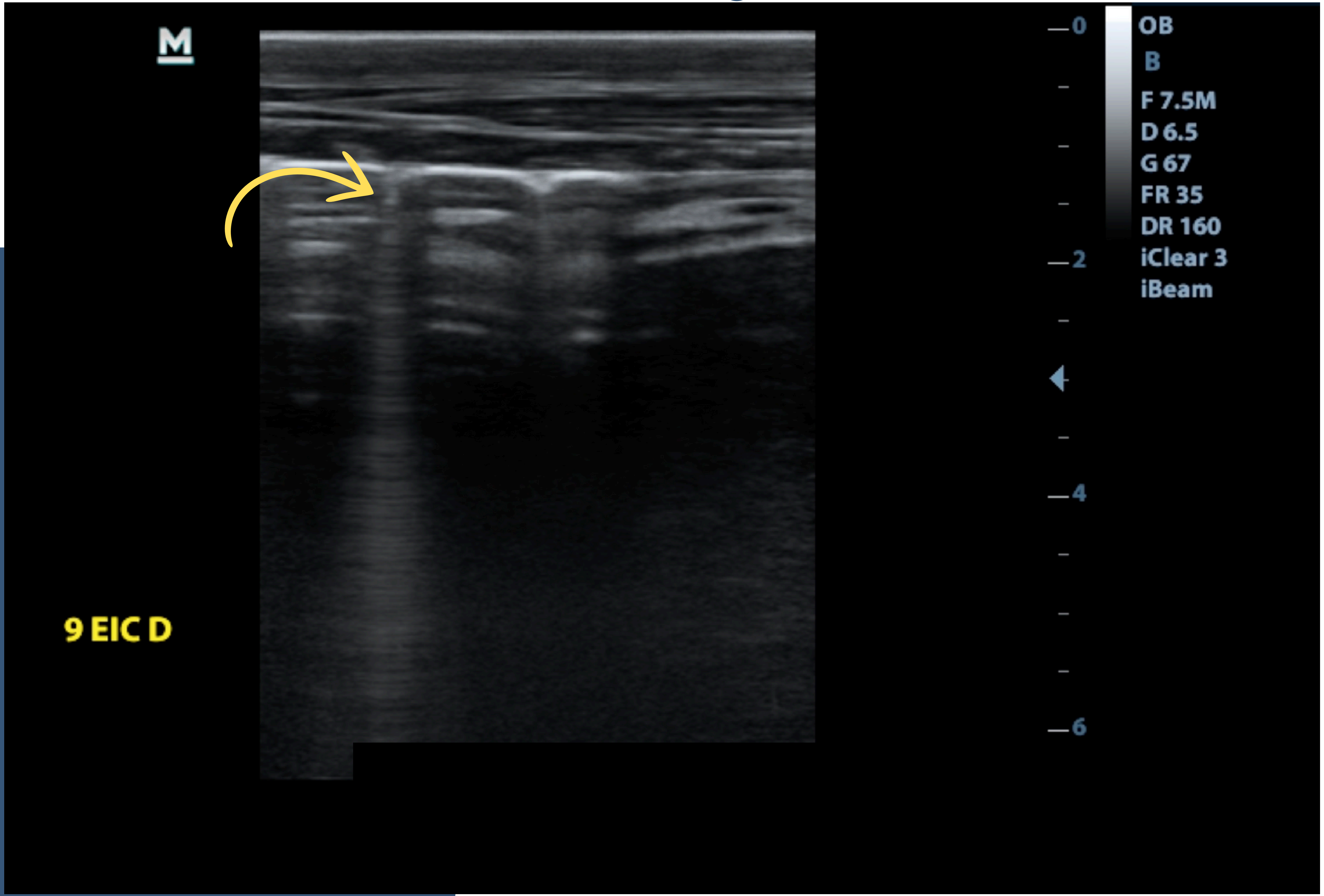

2- The vertical artifacts represents 1/3-2/3 of the pleural line length

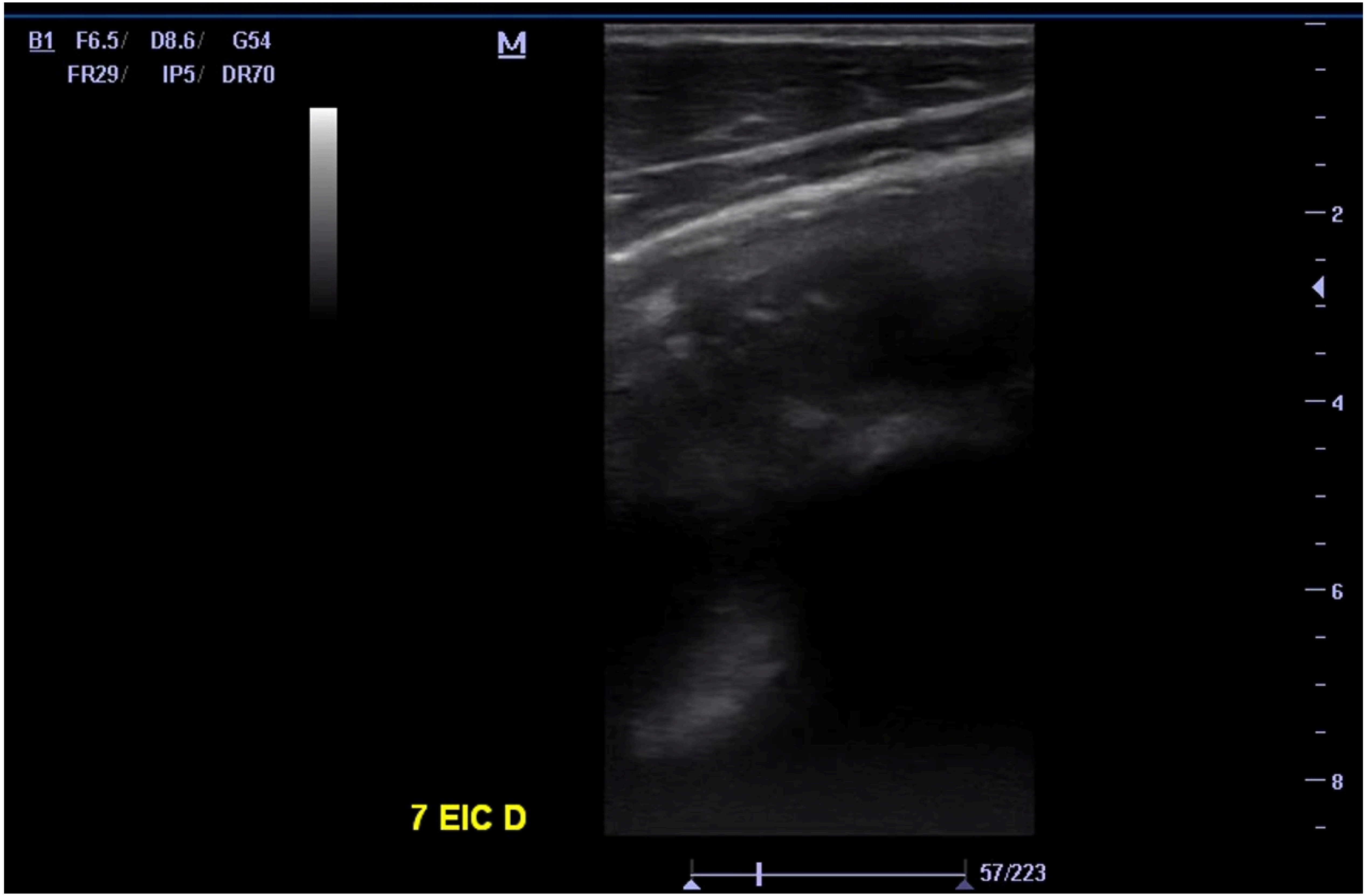

3: Vertical artifacts represent more than 2/3 of the length of the pleural line.

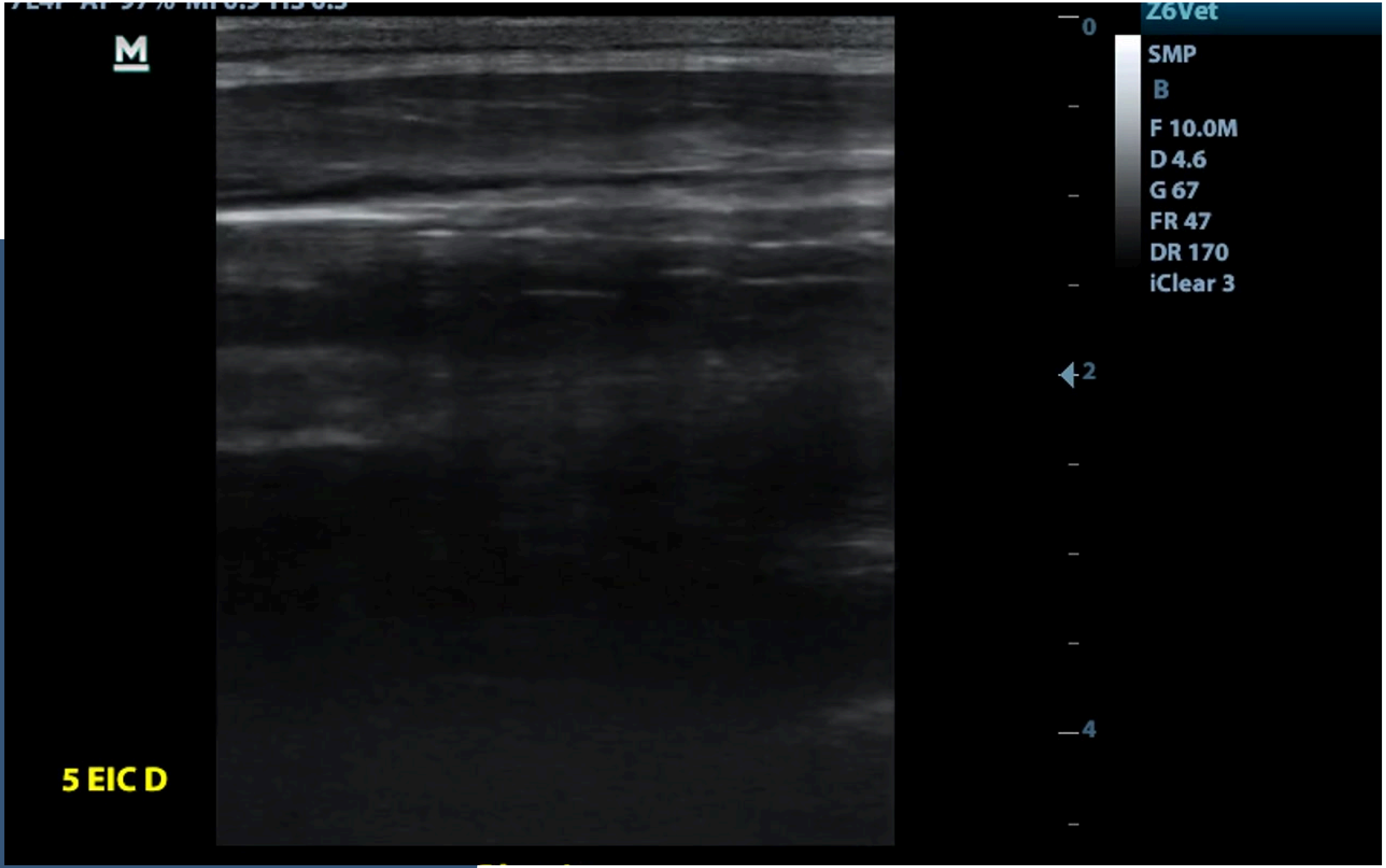

3: Vertical artifacts represent more than 2/3 of the length of the pleural line.

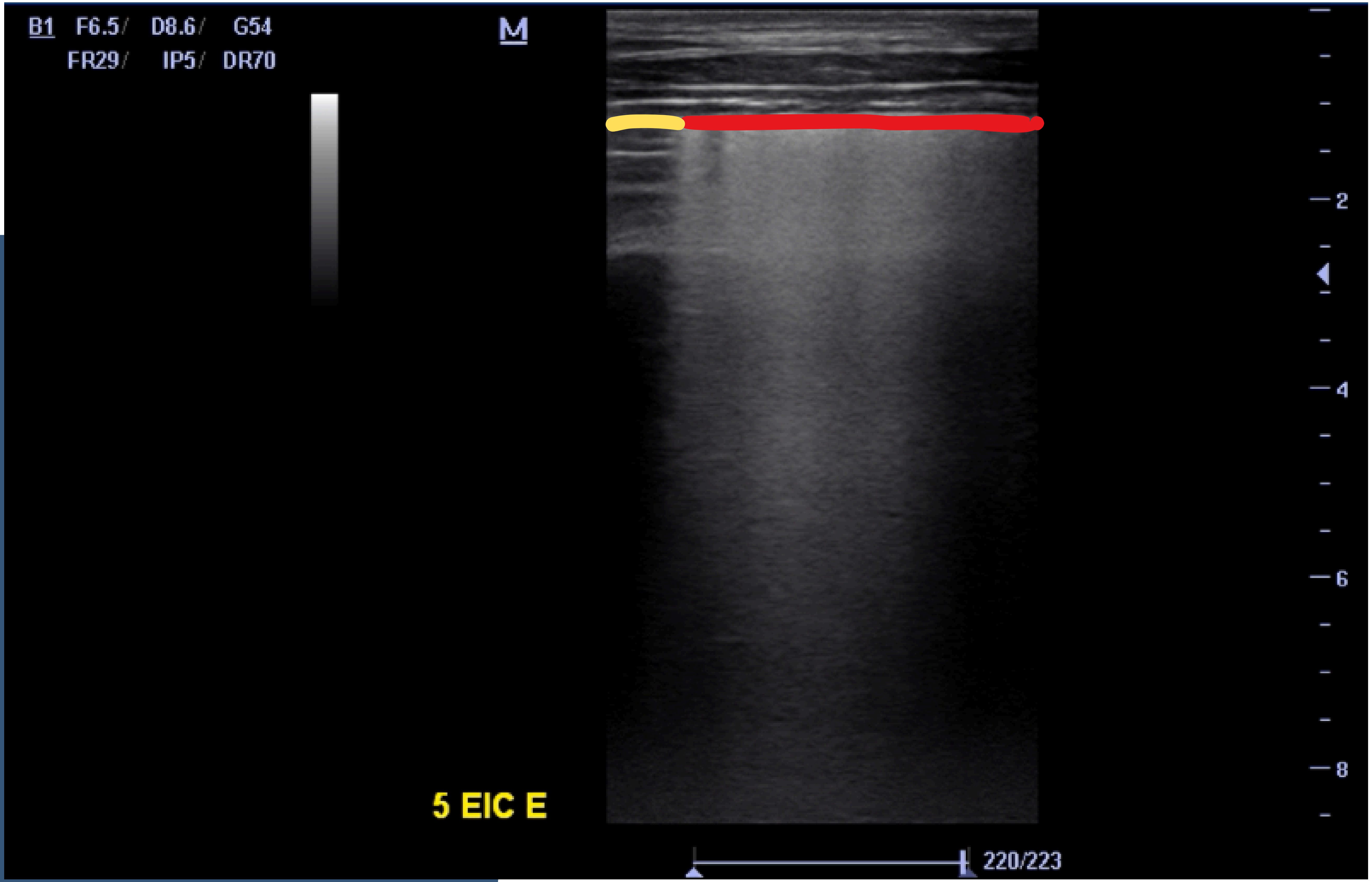

4: Presence of lung consolidation.

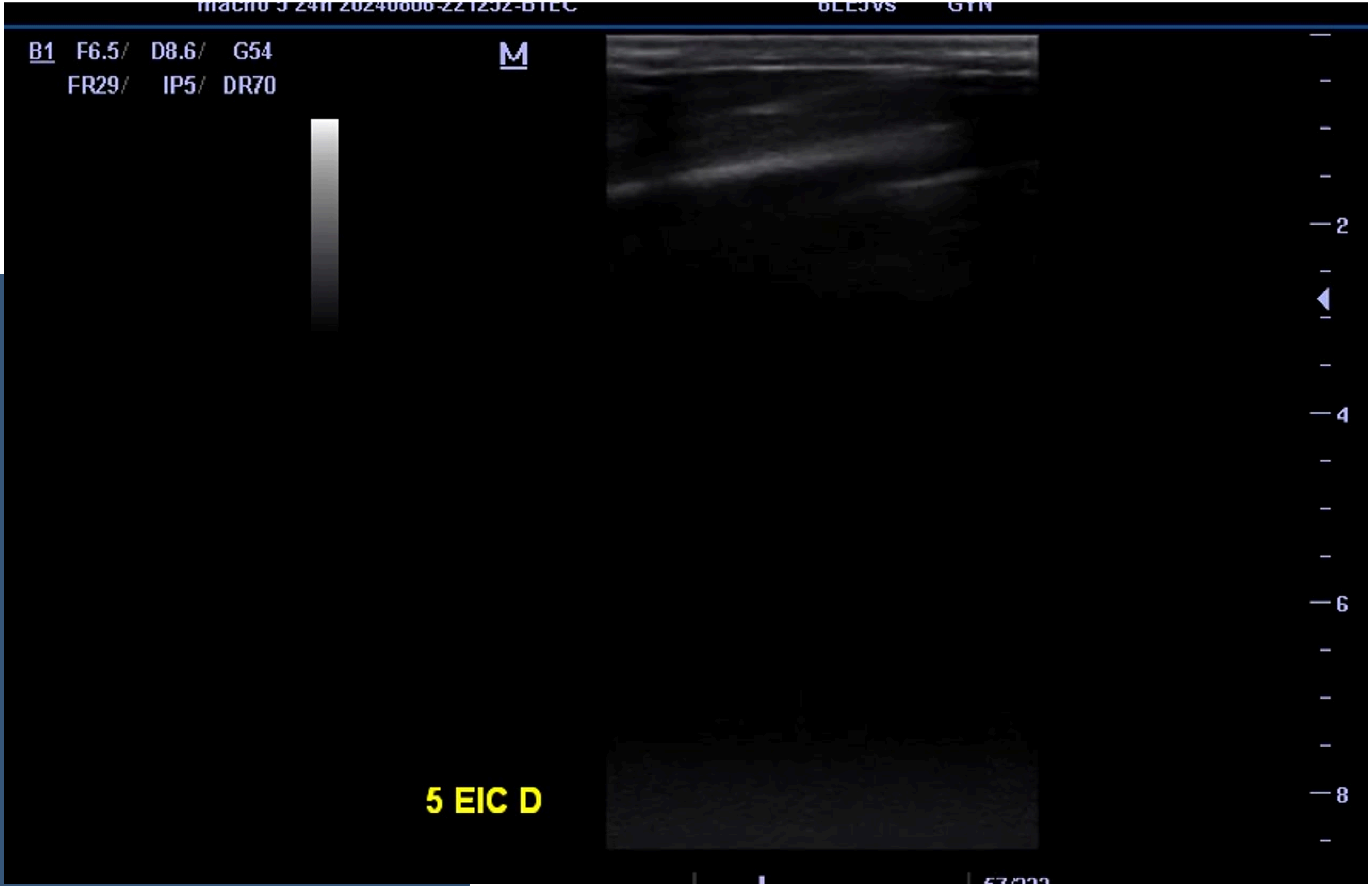

4: Presence of lung consolidation.

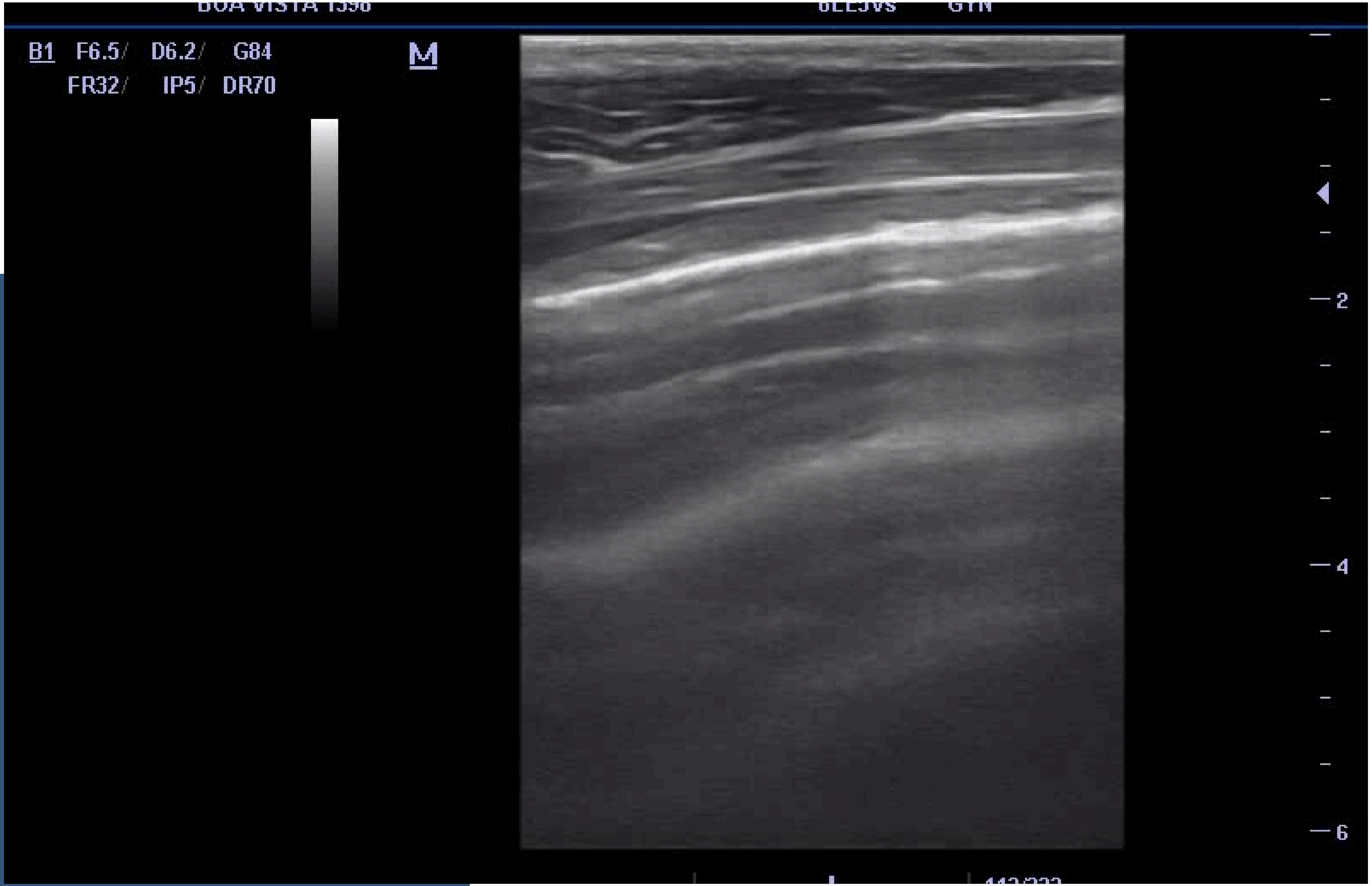

Thank you!

Your participation is very important.

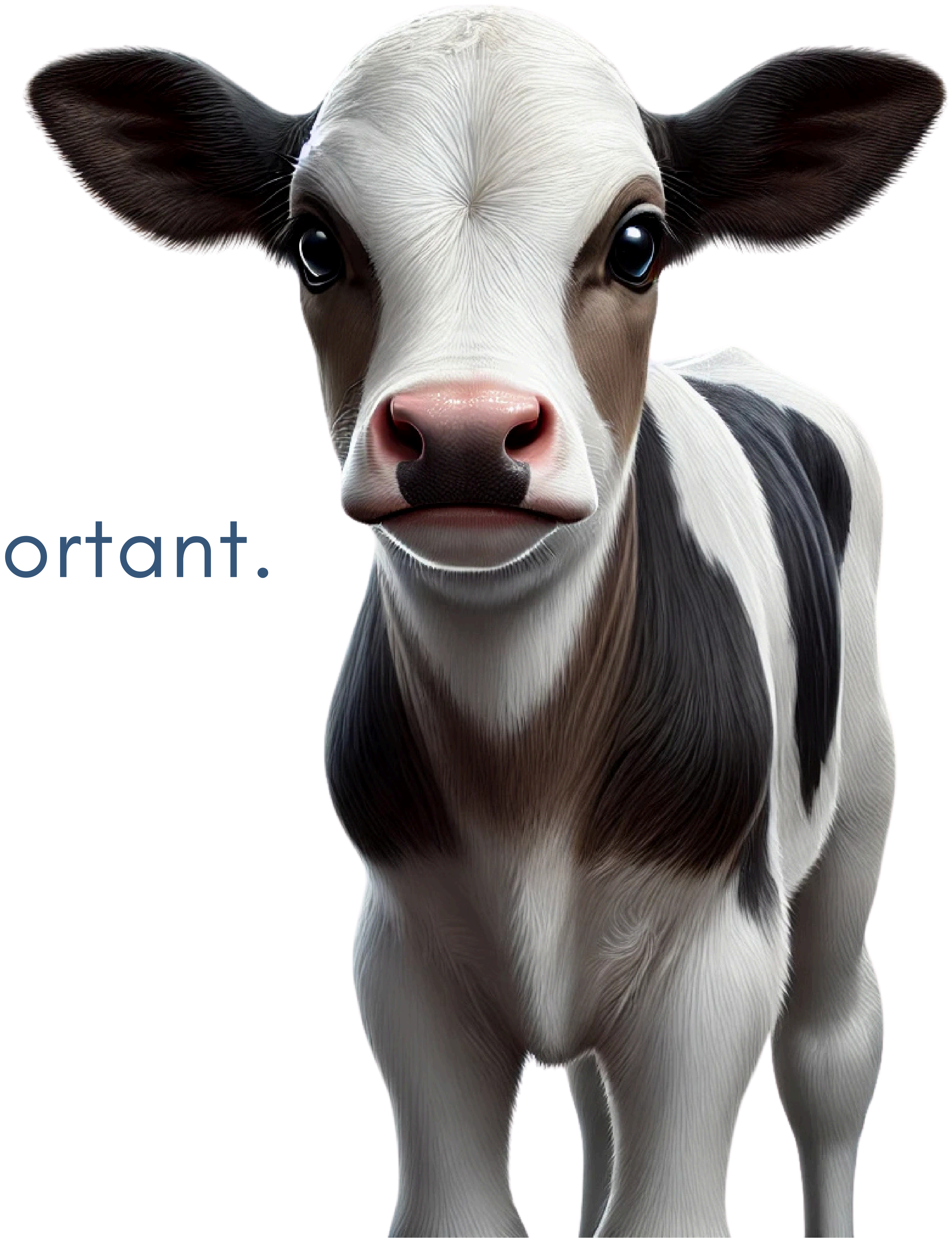

Supplement: aalag067_Supplemental_Files [file aalag067_supplemental_files.zip › supplementary_file_2_aalag067.pdf]
